# γ-Functional Iminiumthiolactones for the Single and Double Modification of Peptides

**DOI:** 10.1021/acs.bioconjchem.3c00424

**Published:** 2023-11-23

**Authors:** Stefan Mommer, Nina Warner, Caroline Lienert

**Affiliations:** Melville Laboratory for Polymer Synthesis, Department of Chemistry, University of Cambridge, Lensfield Road, CB2 1EW Cambridge, U.K.

## Abstract

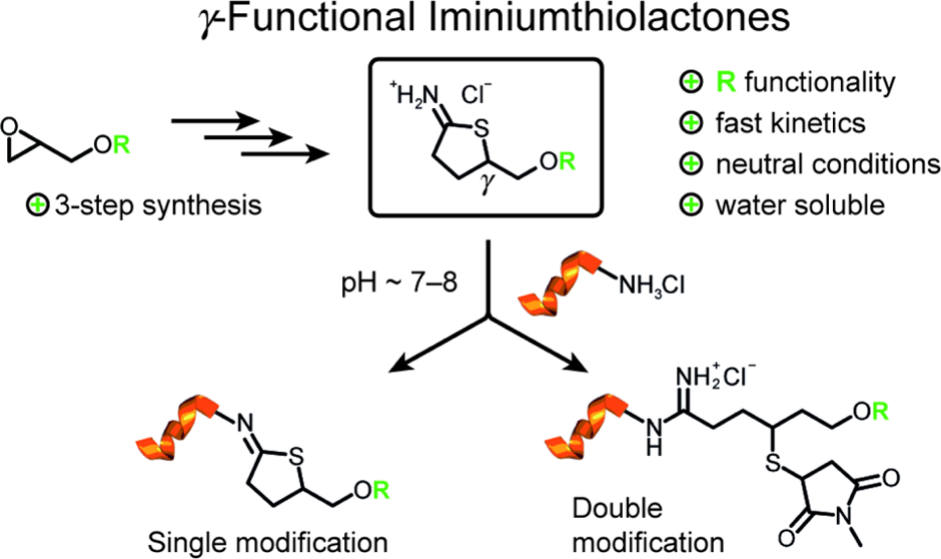

Thiolactones (TL) can be readily incorporated into polymeric materials and have been extensively used as a ligation strategy despite their limited reactivity toward amine-containing substrates. Comparatively, iminiumthiolactones (ITL) are much more reactive, yet to this day, only the nonsubstituted ITL known as Traut’s reagent is commercially available and used. In this work, we advance current TL/ITL chemistry by introducing reactive side groups to the ITL heterocycle in the γ-position, which can be orthogonally modified without affecting the ITL heterocycle itself. To study the reactivity of γ-functional ITLs, we subject one of our derivatives (γ-allyl-functional ITL **3b**) to model reactions with several peptides and a chosen protein (lysozyme C). Using mild reaction conditions, we successfully demonstrate that the γ-functional ITL exhibits orthogonal and enhanced reactivity in a single or double modification while introducing a new functional handle to the biological substrate. We believe that γ-functional ITLs will advance the original Traut chemistry and open promising opportunities for the bioconjugation of biological building blocks to existing functional molecules, polymers, and materials.

## Introduction

Bioconjugate materials are obtained through the conjugation of biological building blocks to polymers or networks and often combine the unique properties of both. While proteins provide biological function, such as enzymatic catalysis, cell signaling, or receptor binding, the conjugated polymer may improve the protein stability and solubility or promote controlled release of the protein or drug bound to it.^1,2^ Consequently, such bioconjugated materials have played an essential role in advancing biotechnology and medical applications, which has led to more effective therapies as well as diagnostics.^2,3^

To access bioconjugate materials, various click chemical reactions have been key, involving the reaction of synthetic groups with those present in proteins.^4−6^ In proteins, amino acids like cysteine, serine, or lysine are, therefore, attractive target groups, with lysine exhibiting both the highest nucleophilicity and average surface accessibility on larger peptides and proteins.^7,8^ Common electrophiles for amines are acyl chlorides, NHS esters, and perfluorinated phenyl esters; however, such active esters release leaving groups, which ultimately have to be removed from the reaction product.^9−11^ Electrophiles that undergo addition reactions (without forming byproducts) are, therefore, preferable, yet highly reactive reagents, such as isocyanates, find limited use in aqueous media where they would be rapidly hydrolyzed.

**Figure 1:**
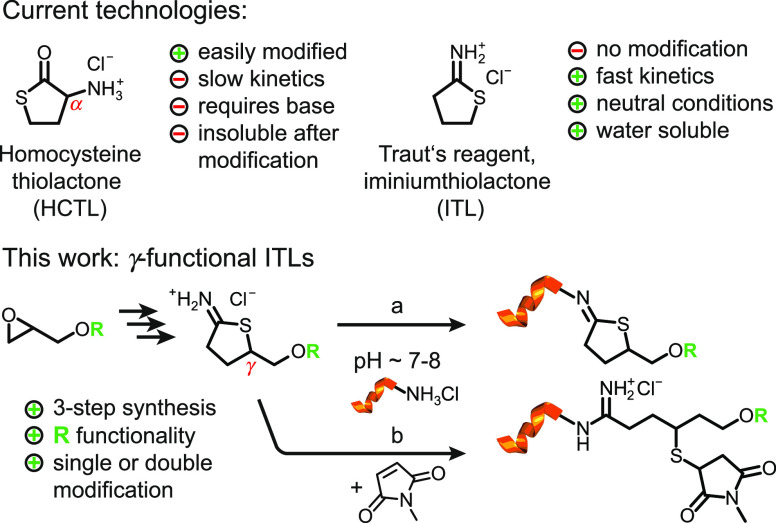
Current technologies: homocysteine thiolactone (HCTL) and Traut’s reagent (ITL), both commercially available. In this work: γ-functional ITLs are obtained through a concise three-step synthesis, allowing to introduction of an additional reactive handle (**R**) to the substrate of interest via single (a) or double modification (b).

In macromolecular engineering, γ-thiolactones (TL) have found widespread use as a ligation technique to and from polymeric building blocks.^12^ In a single modification, the heterocyclic carbonyl undergoes chemo- and regioselective amidation, while in the additional presence of a Michael acceptor (e.g., maleimide), a double modification takes place (ring opening, followed by a thiol–X reaction).^13,14^ The unique reactivity of TLs can be introduced through the commercially available homocysteine thiolactone (HCTL), which can be easily modified through the amine in the α-position (Figure 1). Hence, it has been extensively used for postpolymerization modifications,^15−17^ toward sequence-defined and functional polymers,^18−20^ surfaces,^21,22^ hydrogels,^23,24^ and bioconjugate materials.^25−27^

In one of our previous studies, we demonstrated how selected amino acids and the dipeptide carnosine could be attached to a TL derivative, forming polypeptides/polyelectrolytes in a facile one-pot procedure.^28^ While organic amines readily consume available TL,^29−31^ the reaction with peptidic substrates was only enabled in the presence of equivalent amounts of base.^28^ Recently, Jiang et al. introduced the pair of Ag(I) and 1,4-diazabicyclo[2.2.2]octane (DABCO) to successfully promote the bioconjugation of oligopeptides to TL.^32^ In another example, the polysaccharide pullulan was furnished with intact TLs to undergo peptide-triggered gelation.^23^ When the TL-functional pullulan was mixed and tethered with cell-signaling peptides or gelatin, hydrogels promoted cell growth and proliferation. Due to the inherently reduced reactivity of larger peptides and proteins, however, the amount of reacting thiolactones is hard to control, severely limiting their use in the field of bioconjugate materials.

Analogous to the TL heterocycle, γ-iminiumthiolactone (ITL), also known as Traut’s reagent, is a five-membered ring where the TL carbonyl oxygen is replaced by NH_2_^+^, thus representing an iminium group (Figure 1).^33^ With this small alteration comes a large increase in water solubility and reactivity toward primary amines at a physiologically compatible pH range of pH = 7–9.^34^ Since its invention in 1973, the Traut’s reagent has been continuously used to modify biological molecules (e.g., proteins, enzymes, ribosomes) and anchor them to surfaces,^35,36^ tags,^37,38^ or other (bio)macromolecules.^39,40^ Despite its popularity, Traut’s reagent can only be purchased as an unsubstituted five-membered heterocycle. ITL derivatives with substitutions on the heterocycle have only been reported twice by Carroll and co-workers.^41,42^ In the respective publications, the authors investigated the impact of various alkyl and phenyl substitutions on the reactivity and stability of the ITL derivatives. These modifications, however, did not introduce any additional reactive handle to the substrate, which represents a clear limitation to ITL ligation to date.

In this work, we introduce functional ITLs with reactive groups in the γ-position of the heterocycle, which can be orthogonally modified without affecting the ITL heterocycle. We demonstrate that γ-functional ITLs can be conveniently prepared in a concise three-step synthetic pathway using the appropriate glycidol precursor. Among the synthesized and characterized set of γ-functional ITLs, we studied the reactivity of a chosen example (allyl-functional ITL) toward model amines, peptides, and the protein lysozyme C. Both reaction pathways (single and double modification) that ITLs can undergo are explored, and precise control over the reaction trajectory is demonstrated. Overall, the presented synthetic strategy introduces a new reactive handle to the ITL heterocycle, which could enable its broader use in polymer and (bio)material sciences as highly efficient ligation for biological substrates.

## Results and Discussion

### Synthesis of γ-Functional ITL Derivatives

**Figure 2:**
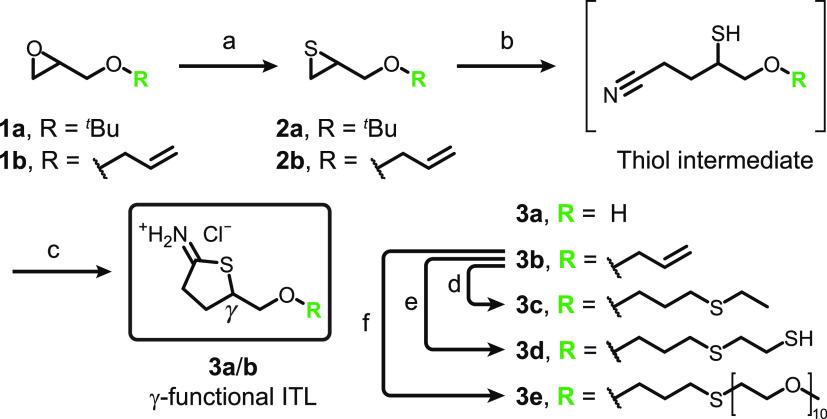
Synthetic pathway toward γ-functional ITLs: (a) KSCN, 25 °C, 2,3-butanediol; (b) MeCN, *n*-BuLi, −78 → 0 °C, THF; (c) EtOH/HCl_(conc)_ (1:1), 25 °C; (d) EtSH, *h*ν, 25 °C, MeCN; (e) 1,2-ethanedithiol, *h*ν, 25 °C; and (f) mPEG-SH, *h*ν, 25 °C.

The γ-functional ITL derivatives are retrosynthetically derived from functional glycidols following a concise three-step synthetic route (Figure 2). Functional glycidols are prominent reagents for anionic polymerization and epoxy resins and are, therefore, commercially available in a wide variety, rendering this synthetic route an attractive strategy.^43^

The first reaction involves the transformation of the functional glycidyl ether **1** to the corresponding thiirane **2** by the use of potassium thiocyanate (KSCN). Due to the exothermic nature of this reaction, the highly reactive thiiranes can polymerize to form undesired poly(episulfide)s. Overall, this was successfully avoided by using 2,3-butanediol as the solvent following a procedure by Endo et al.^44^ An initial attempt to use glycidol for this reaction, however, resulted in various side products and low thiirane yields (26%), presumably due to the interfering free alcohol group (Supporting Information, Section 1.3.1 and Figures S1 and S45). Consequently, *tert*-butyl glycidyl ether **1a** was used as a precursor, giving thiirane **2a** in 90% yield (Figures S2 and S3).

The subsequent synthetic steps are carried out in one pot and comprise a thiirane ring opening, followed by the final cyclization (Figure 2). As such, **2a** was alkylated using lithium acetonitrile to give the corresponding mercaptonitrile through the ring opening of the thiirane (Figure S6). This intermediate was then treated with concentrated hydrochloric acid to undergo *tert*-butyl deprotection and cyclization simultaneously, delivering γ-hydroxymethyl ITL **3a** in 83% yield.^41^

The molecular structure was verified by the use of ^1^H NMR spectroscopy (Figures S4 and S5). The respective spectra of **3a** revealed a characteristic singlet for the iminium proton signal at a chemical shift of δ = 12.21 ppm. In the previously published work by Goff et al., the authors assigned these protons to a broad triplet at δ = 7.40 ppm instead.^41^ During a recrystallization attempt of **3a**, we isolated an unknown precipitate, whose ^1^H NMR spectrum showed only this triplet at δ = 7.40 ppm (Figure S7). Further to this, the triplet exhibited a unique *J*-coupling constant of 50.9 Hz, which had been previously reported for the *J*_^14^NH_ coupling of ammonium ions.^45^ We, therefore, hypothesized that this triplet was caused by ammonium chloride, which could be produced through spontaneous hydrolysis of the iminium group (see the outlined mechanism in Supporting Information; Figure S46). Such hydrolysis reactions had been previously observed for imidates and amidines, too.^46,47^ A comparison between the Fourier-transform infrared (FTIR) spectra of the unknown precipitate and commercially available ammonium chloride confirmed our supposition, as both spectra were identical (Figure S8).

**Figure 3:**
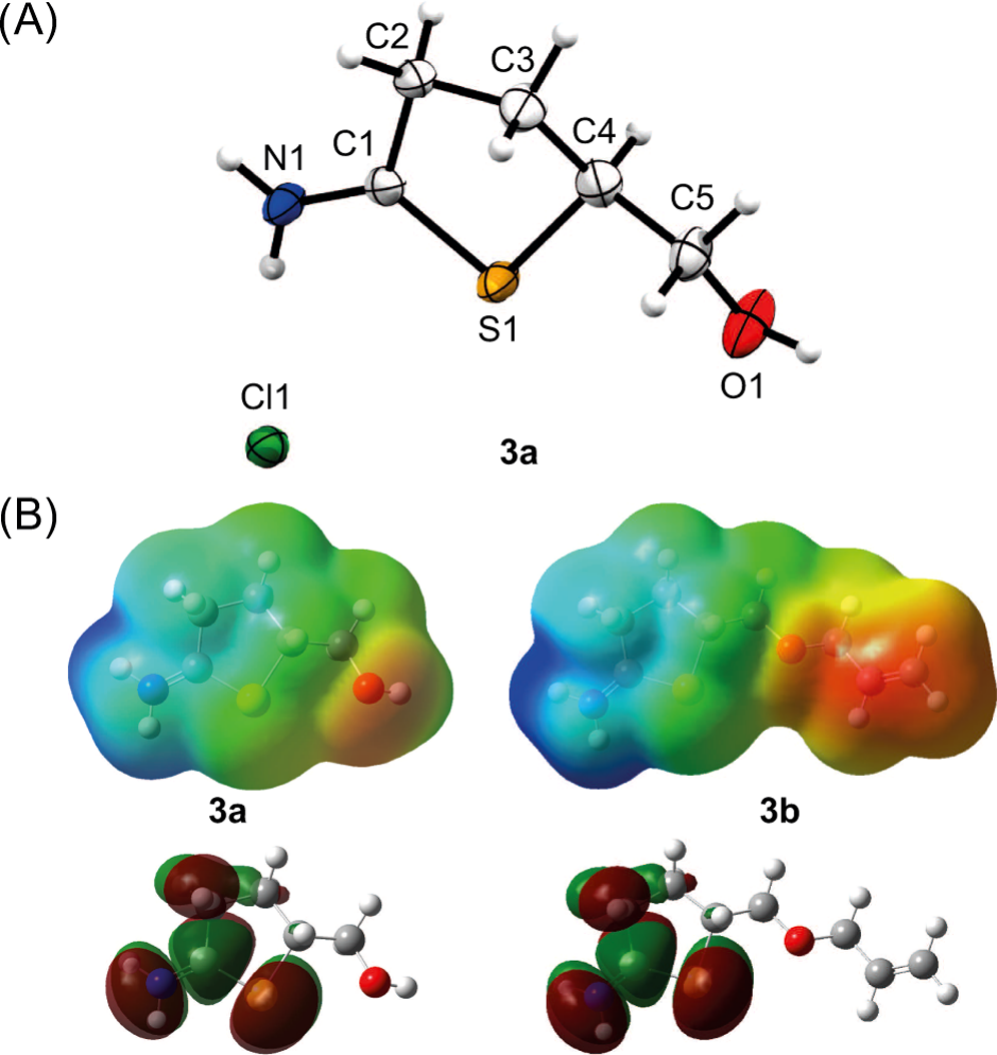
(A) Displacement ellipsoid plot of the asymmetric unit of **3a** showing the major structure of the (S)-isomer (color code: C – gray, H – white, O – red, N – blue, S – yellow, Cl – green). (B) DFT-calculated electron density mapping (top) and visualization of the lowest unoccupied molecular orbitals (LUMO) (bottom) of **3a** and **3b**.

Successful crystallization and single-crystal X-ray diffraction experiments confirmed the general structure of **3a** with respect to its configuration and protonation state (Figure 3A). The packaging unit consists of four chiral molecules in a 1/1 *R*/*S* ratio with the chloride anion stabilized by two =NH_2_^+^ and one hydroxyl group ^1^H NMR spectroscopy hydrogen bonding (Figures 3A, S53, and S54). Further information on data collection and refinement of the crystal structure is given as Supporting Information (Table S4).

Derivative **3b**, which carries an allyl group in the γ-position of the ITL heterocycle, was synthesized in an analogous manner. Starting from allyl glycidyl ether **1b**, allyl thiirane **2b** was obtained in 86% yield (Figure S9). The final allyl ITL **3b** was obtained in 79% yield by telescoping the ring opening of **2b** with subsequent acid-mediated cyclization (Figures S10–S14). Again, the intermediate thiol after the ring opening was successfully isolated for characterizational purposes (Figures S15 and S16).

To gain more information about the stability of our ITL derivatives, we studied their hydrolysis and pH stability in more detail. For this purpose, both **3a** and **3b** were dissolved in 10 mM PBS/D_2_O (0.1 M, pH = 7.4) and monitored over time using ^1^H NMR spectroscopy. The corresponding kinetics showed that over the course of 12 h, 33% hydrolysis occurred, leading to the corresponding thiolactone with little difference between **3a**/**3b** or varying counterions (Cl^–^ vs Br^–^) (Figure S47). The measurements also revealed that hydrolysis was effectively suppressed when the solution was stored at 2 °C. The hydrolysis product (thiolactone) was easily distinguishable from the original ITL, as the proton signal of the methylene group in the α-position of the heterocycle experienced a large upfield shift (Figure S48).

To test the pH stability, we dissolved **3a**/**3b** in D_2_O prior to the addition of small quantities of NaOD (for details, see Supporting Information). In the presence of this rather strong, basic nucleophile, both compounds underwent direct and irreversible ring opening to form an amide (Figure S49), which was confirmed by ^1^H NMR spectroscopy (Figures S50–S52; for further details, see Supporting Information). While the above findings point toward limited pH stability of **3a**/**3b**, they also suggest a possible reactivity toward functional alcohols. The latter would be in agreement with the original Traut’s reagent, which has been used to thiolate polysaccharides in the past.^48^ The reactivity of **3a**/**3b** toward functional alcohols, however, was deemed beyond the scope of this work and may become the substance of a future study.

With the γ-allyl ITL **3b** at hand, we sought to investigate whether the double bond could be orthogonally functionalized without interference with the reactive ITL heterocycle. Consequently, **3b** was subjected to a photoinitiated radical thiol–ene addition as a model reaction. Using ethanethiol, the functionalization was successfully carried out in MeCN to give compound **3c** (Figures S17–S21). Next, **3b** was furnished with a thiol group following previous reaction conditions in neat 1,2-ethanedithiol. The corresponding thiol-functionalized ITL **3d** was obtained in 53% yield (Figures S22–S25). To demonstrate the coupling of ITL **3b** with a polymeric residue, we attached a methoxylated PEG thiol (mPEG-SH) to the allyl double bond using similar conditions. As shown by mass spectrometry data, the PEG-functionalized ITL **3e** was successfully detected, along with the remains of the starting material mPEG-SH (Figure S26 and Tables S1–S3). On account of the near-equal affinity to the stationary phase, the separation of **3e** from mPEG-SH via chromatographic means proved to be challenging, and further optimizations are needed. Nonetheless, it was overall demonstrated that (i) γ-functional ITLs can be successfully sourced from glycidol derivatives and (ii) the allyl group of **3b** offers further opportunities for functionalization, while no adverse reactions with the heterocycle were observed.

### Reactivity & Model Reactions of γ-Allyl ITL

The reactivity of the ITL heterocycle was studied via calculations and model reactions. The ESP-mapped electron density surfaces of **3a** and **3b** are presented in Figure 3B (for the ground-state geometries, see Figures S55 and S56). Calculated at the DFT/B3LYP/6-311++G(2d,p) optimization level, the electron densities of **3a** and **3b** clearly reflect the chemical composition of the heterocycle. The iminium carbon is particularly affected by the neighboring iminium = NH_2_^+^, which exhibits the lowest electron density, thus making the carbon an ideal electrophile. This is in contrast with the corresponding thiolactone surrogates, where the areas of low electron density concentrate on the α-, β- and γ-carbons of the thiolactone ring (Figures S57 and S58). Depictions of the lowest unoccupied molecular orbitals (LUMO) of **3a** and **3b** reveal significant electron-accepting properties centered around the iminium carbon (Figure 3B). Additionally, the molecular orbitals are largely localized around the C–S bond and show an antibonding character. Looking at the orbital energy levels of **3a** and **3b**, both LUMOs are around 1.34 eV lower than the LUMO energy levels of their respective thiolactone surrogates (Figures S59 and S60). Overall, this suggests a superior electron-accepting character of ITLs, enhancing their reactivity toward nucleophiles.

**Figure 4:**
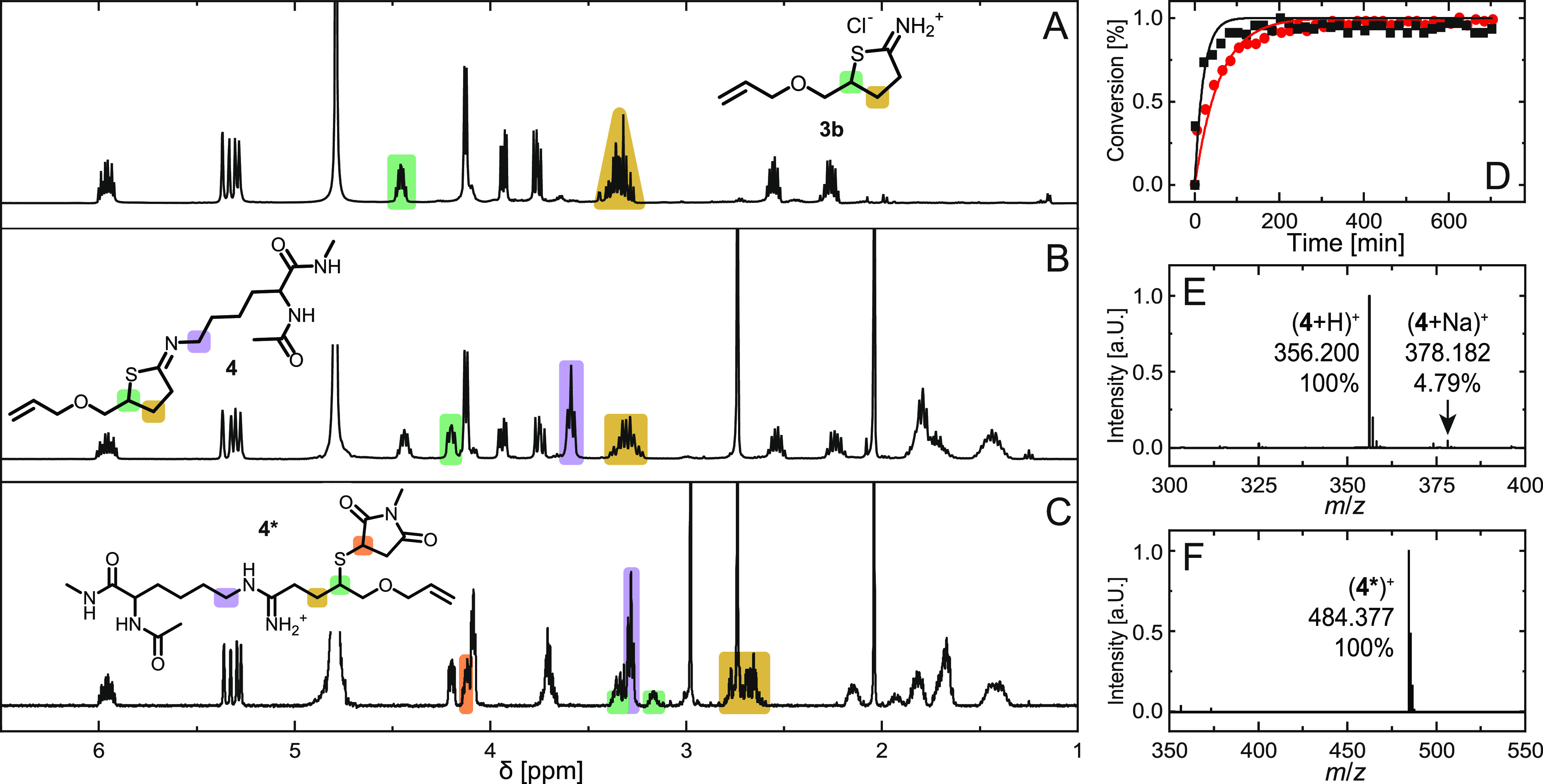
^1^NMR spectra of allyl-functional ITL **3b** (A), *N*_α_-acetyl l-lysine methyl-*N*-amide-modified ITL **4** (B), and 4* (C), recorded in DMSO-*d*_6_. (C) Kinetic data of the ITL single modification with *N*_α_-acetyl-l-lysine methyl-*N*-amide (black solid squares) and 2-aminoethanol (red solid circles). Lines are drawn to guide the eye. Supporting Informationmass spectra of compounds **4** (E) and 4* (F).

**Figure 5:**
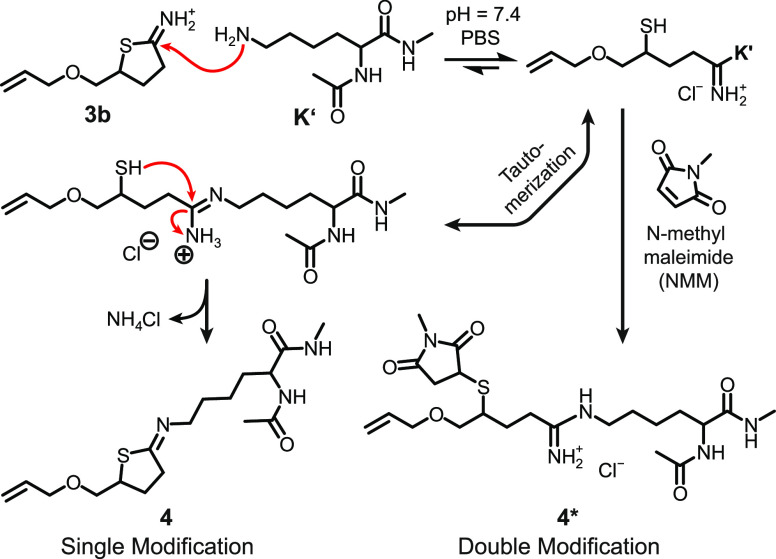
Model reactions of γ-allyl ITL **3b**. Single modification: **3b** is ring-opened by *N*_α_-acetyl-l-lysine methyl-*N*-amide (**K**′) and recyclized under the elimination of ammonium chloride to give *N*-substituted **4**. Double modification: **3b** is ring-opened by **K**′, and the released thiol is trapped by *N*-methylmaleimide to give 4*.

The model reactions were conducted with *N*_α_-acetyl-l-lysine methyl-*N*-amide (**K**′) as a substrate. Methylamide amino acids have been used as model compounds for site-specific modification as well as computational calculations to simulate lysine incorporation in proteins via the two adjacent peptide bonds.^49−51^ The model reactions were carried out at a concentration of 0.1 M in phosphate-buffered saline (PBS, pH = 7.4) and monitored through ^1^H NMR spectroscopy (Figure 4). Regarding starting compound **3b**, ^1^H NMR signals of the methine proton at the stereocenter (δ = 4.56 ppm, green), as well as the adjacent methylene group (δ = 3.34 ppm, yellow), are indicative of chemical modifications on the heterocycle (Figure 4A). Reacting **3b** with **K**′ resulted in an upfield shift of the methine proton to δ = 4.11 ppm (green), while the signal of the β-methylene group remained unchanged (Figure 4B). The latter finding pointed toward the formation of the cyclized neutral product **4** as opposed to a ring-opened thiol-containing species that was initially anticipated. The existence of the **K**′-substituted adduct **4** was confirmed by mass spectrometry displaying singly charged ions (**4** + H)^+^ and (**4** + Na)^+^ (Figure 4E). It, therefore, appears that the aminolysis of **3b** is followed by a nucleophilic attack of the liberated thiol on the amidinium species (Figure 5). Subsequent elimination of ammonium chloride yields the recyclized product with an intact neutrally charged *N*-substituted iminothiolactone structure. Similar observations have been made with the original Traut’s reagent by others.^52^ Complementary to **K**′, **3b** is converted with monoamino ethanol (MEA, an organic amine) to deliver hydroxyethyl-substituted **5**. A comparison of both model reactions revealed slightly slower reaction kinetics when MEA was used (Figure 4D). Detailed ^1^H NMR assignments for both **4** and **5** are found in Supporting Information (Figures S27–S31 and S40–S44).

When **K**′ is reacted with **3b** in the presence of *N*-methylmaleimide (NMM), a different reaction trajectory unfolds, and compound 4* is formed (Figures 4C and 5). This conversion was witnessed by the methine signal of **3b** shifting upfield and splitting into two multiplets at δ = 3.27 and 3.09 ppm (green). Further, the ring opening of **3b** caused an upfield shift of the β-methylene proton signal to δ = 2.73 and 2.53 ppm (yellow). Successful attachment of **K**′ was reflected by the triplet signal of amidinium-adjacent protons at δ = 3.20 ppm (purple). The thiol–ene addition was characterized via the multiplet at δ = 4.04 ppm (orange), which represents the chiral maleimide proton close to the sulfur (Figure 4C). Detailed ^1^H NMR assignments for 4* are found in Supporting Information (Figures S32–S39). Finally, unequivocal proof of product 4* was obtained through mass spectrometry, which shows the singly charged species (4*)^+^ with a characteristic monoisotopic mass of *m*/*z* = 484.377 Da (Figure 4F).

Conclusively, the γ-substituted ITL derivative **3b** is well capable of undergoing chemo- and regioselective modification depending on the present substrates, while the allyl functionality remains unaffected and stays intact. Similar to the thiolactones but with enhanced reactivity, a single or double modification strategy can be chosen, offering fast and efficient reaction kinetics. In the single modification, the thiol that is released through the ring opening of the ITL heterocycle cyclizes and thus gets reprotected to form a cyclic species. As opposed to thiolactones, this behavior can pose a succinct advantage for systems where a permanently liberated thiol would cause adverse reactions.

### Amine-Specific Modification of Peptides

Next, the reactivity of **3b** is investigated toward various peptidic substrates. Besides **K**′, we chose the hexapeptide KFRGDS and the pentapeptide GRGDS. This way, we could probe the lysine and glycine reactivity with minimal changes in the overall peptide structure. Both peptides comprise the characteristic RGD sequence that enables the adhesion of cells to a foreign substrate via transmembrane integrin binding.^53,54^ The covalent attachment of RGD sequences in low amounts toward networks, surfaces, and polymers is a vital condition to facilitate cell–matrix interactions of biohybrid materials. As a third candidate, we chose to investigate the reactivity of KLVFF toward **3b**, representing a rather hydrophobic peptide. The peptide sequence KLVFF is a short fragment of the amyloid β-peptide (Aβ) that promotes Aβ–Aβ interactions, which can result in the formation of amyloid fibrils.^55^ As peptides or protein modifications are usually carried out at lower concentrations, we adjusted accordingly (1 mM) and used reactant ratios of 1:1.1:1.1 (**3b**:nucleophile:NMM). The reactions were carried out in a vial, where regular injections into our HPLC system yielded corresponding kinetic data of the reactions. As reaction media, we used 10 mM PBS (pH = 7.4) or a sodium phosphate buffer (NaPi, pH = 8.0). All products were further characterized by mass spectrometry.

**Figure 6:**
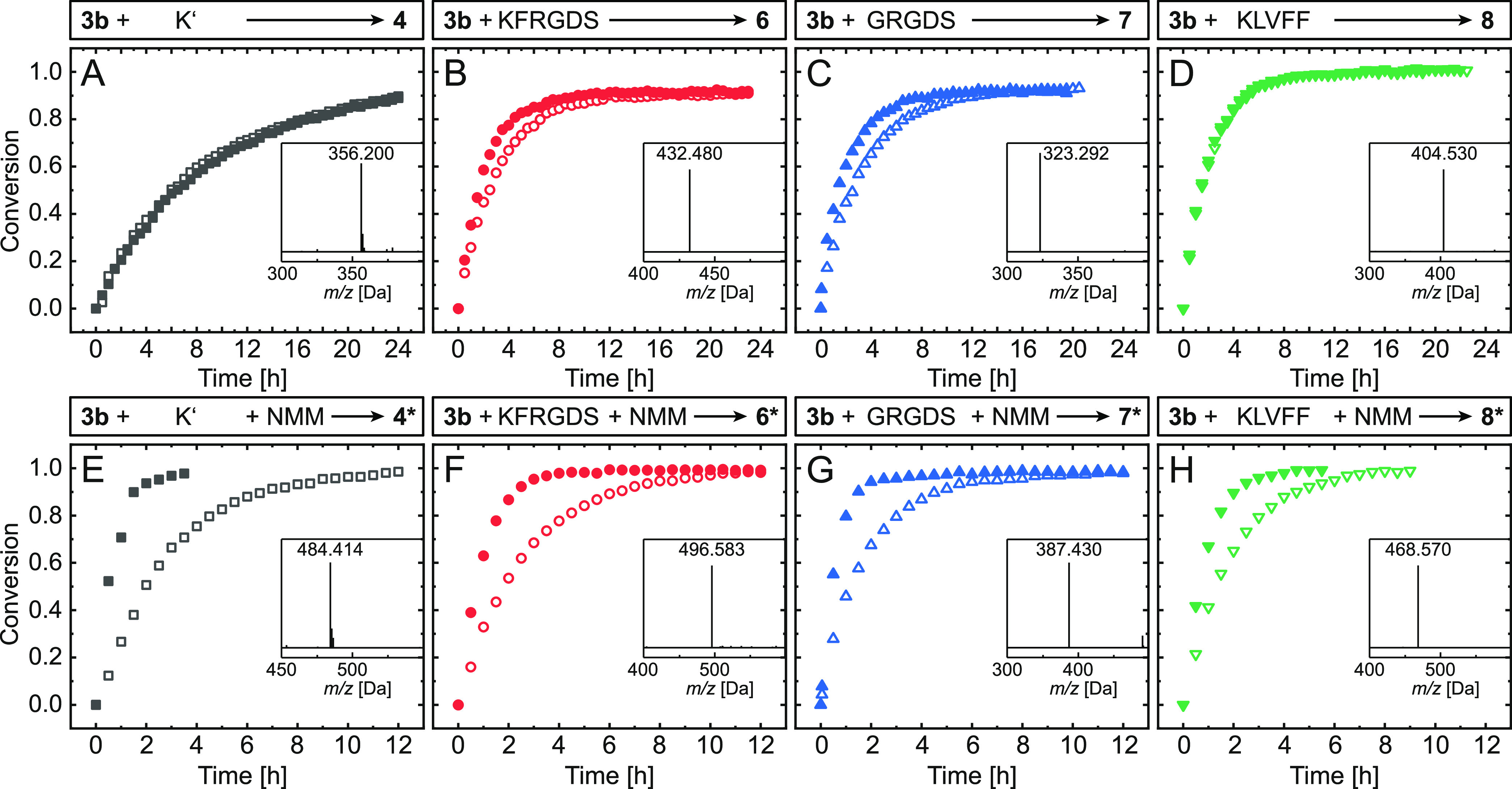
Kinetic data on the reactions of **3b** and various peptidic substrates. Single modifications giving adducts (A) **4**, (B) **6**, (C) **7**, and (D) **8**, as well as double modifications yielding products (E) 4*, (F) 6*, (G) 7*, and (H) 8*. Reactions performed in NaPi (pH = 8.0) and PBS (pH = 7.4) are represented by the data points with full and hollow symbols, respectively. Inset graphs show the corresponding monoisotopic masses of the product species.

Figure 6A–D shows corresponding graphs for the single modification of **3b**. Surprisingly, the reaction of **K**′ with **3b** displays the slowest overall kinetics when compared to the other single modifications (Figures 6A and S61–S63). The conversion reaches 89% over the course of 24 h, showing little difference between the two buffer conditions. Compared to the model reaction at a concentration of 0.1 M (84% conversion after 1 h), slower kinetics are expected due to reduced reactant concentrations. Surprisingly, single modifications involving peptides proceeded faster. The reaction of **3b** with KFRGDS toward adduct **6** (Figure 6B) reaches 78 and 67% conversion after 4 h in NaPi and PBS, respectively, reaching a plateau at 91% (Figures S64–S66). Mass spectrometry shows the doubly charged species (**6** + 2H)^2+^ = 432.480 Da. When **3b** is converted with GRGDS to form adduct **7**, a similar reaction rate is observed (Figures 6C, S67, and S68). After 4 h, 78% conversion is reached in NaPi, while the reaction in PBS shows 65%, both plateauing at approximately 91%. The mass spectrum shows the singly charged species (**7** + H)^+^ = 645.500 Da and doubly charged species (**7** + 2H)^2+^ = 323.292 Da (Figure 6C inset graph, Figure S69). Single modification of **3b** with KLVFF to deliver product **8** reaches 85% conversion within 4 h of reaction time (Figures 6D, S70, and S71). The conversion saturates at 98%, and the influence of pH on the reaction kinetics is negligible. The product species is detected as singly and double charged species (**8** + H)^+^ = 807.690 Da and (**8** + 2H)^2+^ = 404.530 Da (Figure 6D inset graph, Figure S72). Overall, the influence of the buffer on the kinetics of the single modification remains limited, and the recyclization of the ring-opened intermediate appears to be the rate-determining step.

The double modifications proceed in the presence of NMM as a thiol trapping agent. Transformation of **3b** to 4* is completed within 4 h at a slightly basic pH (NaPi, Figures 6E and S73). The reaction in PBS reaches 75% conversion at an equal duration (Figure S74). Double modification using KFRGDS toward 6* reaches 97 and 78% conversion within the first 4 h using NaPi and PBS, respectively (Figures 6F, S76, and S77). Adduct 6* was confirmed by mass spectrometry as a doubly charged species (6* + 2H)^2+^ = 496.583 Da (Figure 6F inset graph, Figure S78). When GRGDS is reacted with **3b** to give 7*, 4 h reaction time results in conversions of 97 and 87% in NaPi and PBS, respectively (Figures 6G, S79, and S80). The mass spectrum reveals the correct monoisotopic mass for the doubly charged species (7* + H)^2+^ = 387.430 Da (Figure 6G inset graph, Figure S81). Finally, modification of **3b** with peptide KLVFF to deliver 8* is completed within 4 h in NaPi (97%, Figures 6H and S82). The kinetic data for PBS show slightly slower conversion, reaching 88% after 4 h of reaction time (Figure S83). Product formation was confirmed via mass spectrometry displaying the species (8* + H)^2+^ = 468.570 Da (Figures 6H and S84). As opposed to the single modification, the usage of the slightly basic NaPi buffer significantly enhances the overall reaction kinetics. In the presence of NMM, the previously rate-limiting recyclization is successfully suppressed, and the much faster thiol-maleimide addition takes place.

It is important to note that throughout all HPLC kinetics, hydrolysis of **3b** or any of the formed products was not observed (up to 24 h). This is despite the reactant concentration being reduced 100-fold when compared to the performed model reactions (1 mM vs 0.1 M). In addition, adverse reactions of the ITL heterocycle with potentially reactive side groups, such as guanidines (arginine), alcohols (serine), or acids (aspartic acid, C-terminus) did not occur, being in line with what has been reported for the original Traut’s reagent under similar reaction conditions.^56^ The HPLC traces and mass spectra, therefore, indicated that both single and double modifications of the ITL heterocycles selectively yielded stable products.

In conclusion, both the single and double modification of **3b** could be successfully carried out using near-stoichiometric amounts of various peptides at low concentrations and benign reaction conditions to selectively yield the hydrolytically stable adducts **4**, **6**-**8** and 4*, 6*-8*. The presented kinetic data show that the functionalization in the γ-position of the heterocycle does not compromise the reactivity of **3b**, confirming the highly efficient nature of the ITL ligation.

### Modification of Lysozyme C

Finally, we wanted to probe the capacity of our allyl-functionalized ITL derivative **3b** to react with a more complex substrate, such as a protein. As proteins possess higher molecular weights, their reactivity is often reduced. Additionally, potential reactive groups might be buried inside the tertiary protein structure and therefore are less accessible to react with the chosen reagent. As a model protein, we chose hen-egg white lysozyme, also known as lysozyme C. Lysozymes are immunologically important, as they provide defense against bacteria by damaging their cell walls.^57^ Further to this, lysozyme C has been well characterized. Out of its 129 amino acid residues, six are lysines located at the periphery, amounting to a molar mass of 14,305 Da. Moreover, lysozyme C is thermally stable up to 72 °C and maintains a large activity range (pH = 7–9) with an isoelectric point of pI = 11.35.^58^ It is, therefore, the ideal candidate for our modification, and the following conditions were applied for its modification with **3b**: The protein concentration was set to 50 μM, and NaPi buffer was used as the reaction medium. The reaction was started by the addition of 1 equiv **3b** for the single modification and 1 equiv of both **3b** and NMM for the double modification. After 24 h, samples were frozen to halt the reaction prior to their analysis by mass spectrometry. The MaxEnt algorithm was used for statistical deconvolution of the combined ion series.

**Figure 7:**
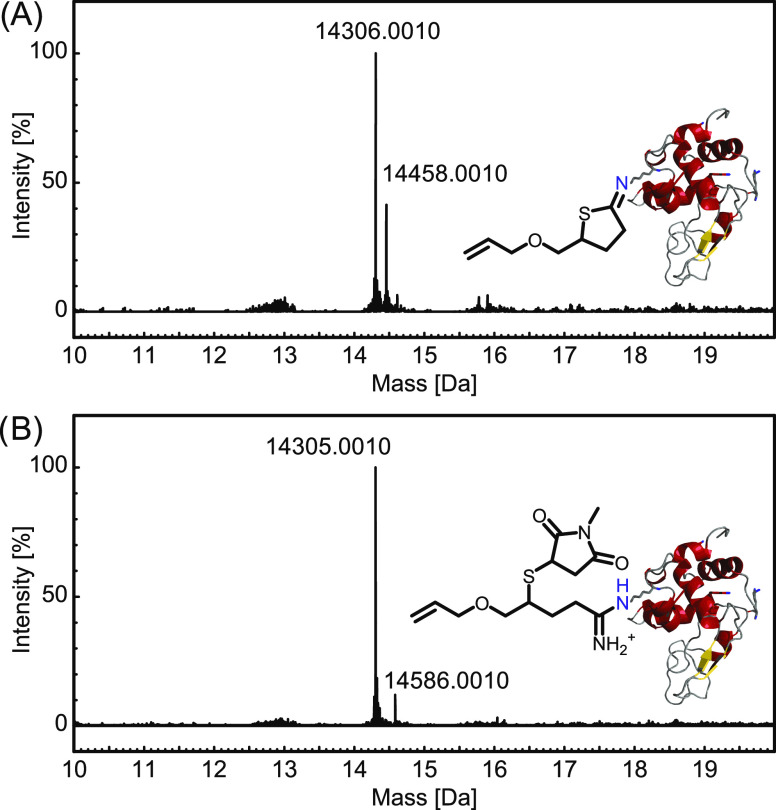
MaxEnt deconvoluted mass spectra showing (A) the single modification of lysozyme C and (B) the double modification of lysozyme C using **3b** and NMM.

After the reaction of lysozyme C and 1 equiv of **3b**, the major component shown in the mass spectrum is represented by the remains of the nonmodified lysozyme C with a mass of 14,306 Da (Figure 7A). A second peak was observed with approximately 40% intensity and a mass of 14,458 Da. The mass difference of Δ = 152 Da corresponds to the addition of one molecule of **3b** (Figure 7A), which confirms the successful modification of our model protein. When one equivalent of both **3b** and NMM is used to favor the double modification of lysozyme C, a different mass peak appears (Figure 7B). The peak with an approximate intensity of 13% and a mass of 14,586 Da corresponds to a mass difference of Δ = 281 Da and can be assigned to a modified species that underwent exactly one double modification. At the presented stoichiometric ratios, no higher degrees of substitution were observed within the 24 h reaction time, yet the overall conversions remain limited. To reach higher conversions, each respective modification reaction was attempted using higher quantities of **3b** and NMM (10, 20, 30, and 50 equiv, Tables S6–S13 and Figures S85–S92). Using excess of the reagents resulted in higher conversions but also higher degrees of substitution, with mono-, di-, tri-, and tetra-substituted products. Overall, the reaction conditions can be adjusted depending on the needs of a given system, and therefore, **3b** shows good potential to be used as a chemical anchor and/or ligation chemistry to combine synthetic and biological building blocks while introducing another reaction handle to the substrate. Further implementation of this ligation strategy into polymeric systems to enable the facile synthesis of biohybrid materials is ongoing.

## Conclusions

In this article, we advanced current TL/ITL ligations by introducing functional groups in the γ-position of iminiumthiolactone derivatives. Compared to many conventional TLs, ITLs exhibited innate water solubility and increased reactivity toward amine-containing substrates; however, substitutions on the heterocycle have been scarcely explored. We synthesized various γ-functional ITL derivatives and performed orthogonal modifications on the double bond of **3b**. These functionalizations did not interfere with the ITL heterocycle. The allyl-functionalized ITL **3b** was chosen to study model reactions and reaction kinetics. Together with the lysine derivative **K**′, **3b** underwent single or double modification (in the presence of NMM), forming either a recyclized *N*-substituted ITL or a positively charged amidinium species. Both pathways were precisely monitored, and signal changes were fully assigned by the use of ^1^H NMR spectroscopy. Next, **K**′ and three different peptides were separately converted with **3b** in two different buffers at a 10-fold decreased concentration. Using these benign reaction conditions, the single modification was rate-limited by the recyclization step, while double modifications were much faster, and a slightly basic environment accelerated the reaction kinetics further (NaPi buffer, pH = 8.0). Finally, using **3b**, single and double modifications on the protein lysozyme C were performed at concentrations of 50 μM. Despite limited conversions, only singly substituted species were detected, confirming the cleanliness of this strategy. Additionally, using higher quantities of the reagents increased both conversion and degree of modification of the protein. In all performed model reactions using **3b**, the allyl group was maintained and therefore introduced an additional reactive handle to the substrate. As such, we believe that using γ-functional ITLs as a ligation strategy may find wider application in the fields of bioconjugate, polymer, and material sciences and aim to further explore γ-functional ITLs in these areas.

## Supporting Information Available

Experimental part; synthetic procedures; NMR spectra; NMR kinetic measurements (thiirane syntheses, model reactions); hydrolysis and pH stability; crystallographic data; computational calculations; HPLC kinetic measurements including elugrams and mass spectrometric analyses, and additional data and mass spectra on the protein modification (PDF)
