# Supplementary material for: γ-Functional Iminiumthiolactones for the Single and Double Modification of Peptides
Source: Bioconjug Chem. 2023 Nov 23;34(12):2302–10. doi: 10.1021/acs.bioconjchem.3c00424 (PMC10739594; doi:10.1021/acs.bioconjchem.3c00424)
Supplement: Supplementary file 1 — bc3c00424_si_001.pdf [file bc3c00424_si_001.pdf]

Supporting Information

γ-Functional Iminiumthiolactones for the Single and Double Modification of Peptides

Stefan Mommer,[†,‡]* Nina Warner[†] and Caroline Lienert[†]

[†] Melville Laboratory for Polymer Synthesis, Department of Chemistry, University of Cambridge, Lensfield Road, CB2 1EW Cambridge (UK).

[‡] Present address: Macromolecular Engineering Laboratory, ETH Zurich, Sonneggstrasse 3, 8092 Zurich (Switzerland).

* Corresponding author. E-mail: smommer@ethz.ch

Table of contents

1 Experimental part 2
1.1 Materials 2
1.2 Measurements 2
1.3 Synthesis 3
2 NMR kinetic measurements 33
2.1 Thiirane syntheses 33
2.2 Model reaction kinetics 33
2.3 Hydrolysis and pH stability of ITLs 33
3 Crystallographic data of 3a 38
4 Calculations 41
5 HPLC kinetic measurements 46
6 Protein modification 56
7 References 62

1 Experimental part

1.1 Materials

Glycidol (96%, Sigma-Aldrich), tert-butyl glycidyl ether (99%, Sigma-Aldrich), Allyl glycidyl ether (≥99%, Sigma-Aldrich), potassium thiocyanate (reagent grade, Alfa Aesar), 2,3-butanediol (98%, Acros Organics), magnesium sulfate – dry (reagent grade, Alfa Aesar), n-butyl lithium (n-BuLi, 1.6 M in hexane, Acros Organics), ethanethiol (97%, Sigma-Aldrich), 1,2-ethanedithiol (>95%, Sigma-Aldrich), Irgacure D2959 (98%, Sigma-Aldrich), ethanolamine (≥99%, Sigma-Aldrich), Nα-Acetyl-L-lysine methyl amide (ChemCruz) were used without further purification. Unless otherwise indicated, all solvents were purchased from commercial sources and were used without further purification.

1.2 Measurements

^{1}H and ^{13}C NMR spectra were recorded on a Bruker DPX-400 FT NMR spectrometer (400 MHz and 100 MHz, respectively) and are reported as follows: chemical shift δ (ppm) (multiplicity, coupling constant *J* (Hz), number of protons, assignment). $CDCl_3$ (δ_H = 7.26 ppm, δ_C = 77.0 ppm), dimethylsulfoxide (DMSO-d_6, δ_H = 2.50 ppm, δ_C = 39.5 ppm) and D_2O (δ_H = 4.79 ppm) were used as an internal standard. Chemical shifts are reported in ppm to the nearest 0.01 ppm for ^{1}H and the nearest 0.1 ppm for ^{13}C.

Molecular weights (M_n and M_w) and dispersity values (M_w/M_n) were determined by size exclusion chromatography (SEC). SEC analyses were carried out with water as eluent. SEC with water (HPLC grade, VWR) as eluent was performed using a Shimadzu LC system equipped with a diode-array detector (SPD-M20A DAD), a refractive index detector (RI, Wyatt Optilab Rex) and a multi-angle laser light scattering detector (MALLS, Wyatt Dawn Helios Two). The eluent contained 0.1M $NaNO_3$ (98%, Breckland Scientific Supplies) and 0.01M NaN_3 (≥99%, Sigma Aldrich). One precolumn (50x8 mm) and four PSS Suprema gel columns (300x8 mm) were applied at a flow rate of 1.0 mL·min^{-1} at 25 °C. The diameter of the gel particles measured 10 µm, the nominal pore widths were 30, 100 and twice 3000 Å.

Infrared spectra were measured on a Spectrum 100 FT-IR spectrometer (PerkinElmer) using an ATR Sampling Accessory unit (Perkin Elmer). Absorbance maxima are reported in wavenumbers (cm^{-1}) and only selected intensities are reported.

HRMS/ESI mass spectra were recorded on a Xevo G2-S ASAP spectrometer (Waters) and/or on a Thermo Scientific LTQ Orbitrap XL spectrometer.

High Pressure Liquid Chromatography (HPLC) was performed on a Varian 940-LC Series to purify and collect water-soluble species by using a Phenomenex C18 Kinetic-Evo column with a 5 micron pore size, a 110 Å particle size and with the dimensions 150 x 21.2 mm. A gradient

from 5% acetonitrile 95% water (+ 0.1% TFA) to 100% acetonitrile was run at a flow rate of 10 mL/min over 20 min.

All peptide sequences were synthesized using solid-phase methodology (FMOC, tBu, MBHA resin) on an automated microwave peptide synthesizer (Liberty, CEM). Crude peptides were cleaved from the resin with a mixture of 95% trifluoroacetic acid, 2.5% triisopropyl silane and 2.5% water and left to shake for 2.5 h. Following cleavage, the crude peptides were precipitated and washed with cold diethyl ether (DEE), then left to dry under vacuum overnight. The crude peptides were then purified by high pressure liquid chromatography (HPLC) using a Phenomenex C18 Kinetic-Evo column with a 5 micron pore size, a 110 Å particle size and with the dimensions 150 x 21.2 mm. A gradient from 5% acetonitrile 95% water to 100% acetonitrile was run with 0.1% ammonium hydroxide and 20 mM ammonium formate to prevent aggregation.

1.3 Synthesis

1.3.1 2-hydroxymethylthiirane

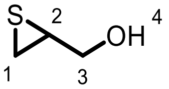

Potassium thiocyanate (14.7 g, 151 mmol) was added to a stirred solution of glycidol (5.00 mL, 75.4 mmol) in 2,3-butanediol (15.1 mL, c = 5 M) at room temperature. The reaction mixture was stirred at maximum 25 °C for 1.5 h. Then, the reaction mixture was extracted with pentane (20 mL) and the layers were separated. Next, water (30 mL) was added to the remaining organic mixture (2,3-butanediol phase) and extracted with CH_2Cl_2 (3 x 20 mL). The combined organic extracts were dried ($MgSO_4$) and the solvent was evaporated under reduced pressure. Purification by flash column chromatography on silica gel using CH_2Cl_2 as eluent gave 2-hydroxymethylthiirane (1.78 g, 26%) as colorless liquid. Spectroscopic data was consistent with those reported in literature.[1] R_F (CH_2Cl_2) 0.35. ^{1}H NMR (400 MHz, Chloroform-*d*) δ 3.91 (dd, J = 11.9, 4.2 Hz, 1H, H3), 3.65 (dd, J = 11.9, 5.1 Hz, 1H, H3), 3.20 (dtd, J = 6.4, 5.3, 4.2 Hz, 1H, H2), 2.48 (dd, J = 6.4, 1.2 Hz, 1H, H1), 2.33 (dd, J = 5.5, 1.2 Hz, 1H, H1), 2.04 (br. s, 1H, H4).

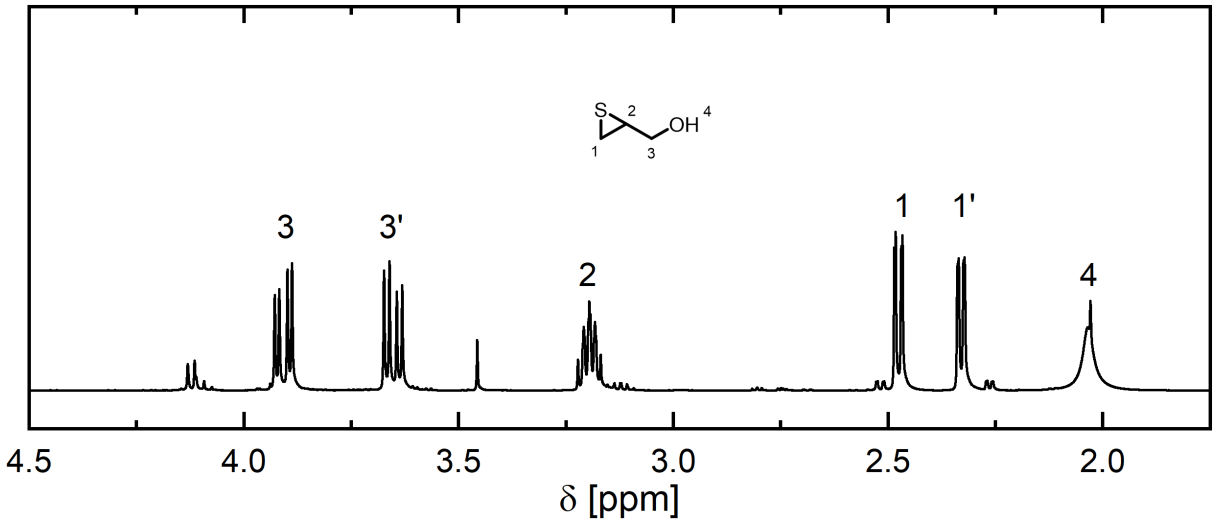

Figure S1. ^{1}H NMR spectrum of 2-hydroxymethylthiirane. Recorded in chloroform-d.

1.3.2 2-(tert-butoxymethyl)thiirane (**2a**)

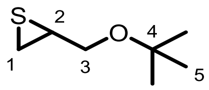

2a

Potassium thiocyanate (14.9 g, 154 mmol) was added to a stirred solution of tert-butyl glycidyl ether **1a** (10.0 mL, 76.8 mmol) in 2,3-butanediol (22 mL, c = 5 M) at room temperature. The reaction mixture was stirred at room temperature for 5 h. Then, the reaction mixture was extracted with pentane (50 mL) and the layers were separated. Next, water (50 mL) was added to the remaining organic mixture and extracted with pentane (2 x 50 mL). The combined organic extracts were dried ($MgSO_4$) and the solvent was evaporated reduced pressure. Purification by flash column chromatography on silica gel using CH_2Cl_2 as eluent gave thiirane **2a** (10.1 g, 90%) as colorless liquid. Spectroscopic data was consistent with those reported in literature.[2] R_F (CH_2Cl_2) 0.43. ^{1}H NMR (400 MHz, Chloroform-*d*) δ 3.66 (ddd, *J* = 9.9, 5.3, 1H, H3), 3.23 (dd, *J* = 9.9, 7.2 Hz, 1H, H3), 3.03 (ddt, *J* = 7.2, 6.0, 5.3 Hz, 1H, H2), 2.53 (dt, *J* = 6.0 Hz, 1H, H1), 2.21 (dd, *J* = 5.3, 1H, H1), 1.20 (s, 9H, H5). ^{13}C NMR (101 MHz, $CDCl_3$) δ 73.7 (C4), 67.2 (C3), 33.5 (C2), 27.7 (C5), 24.7 (C1).

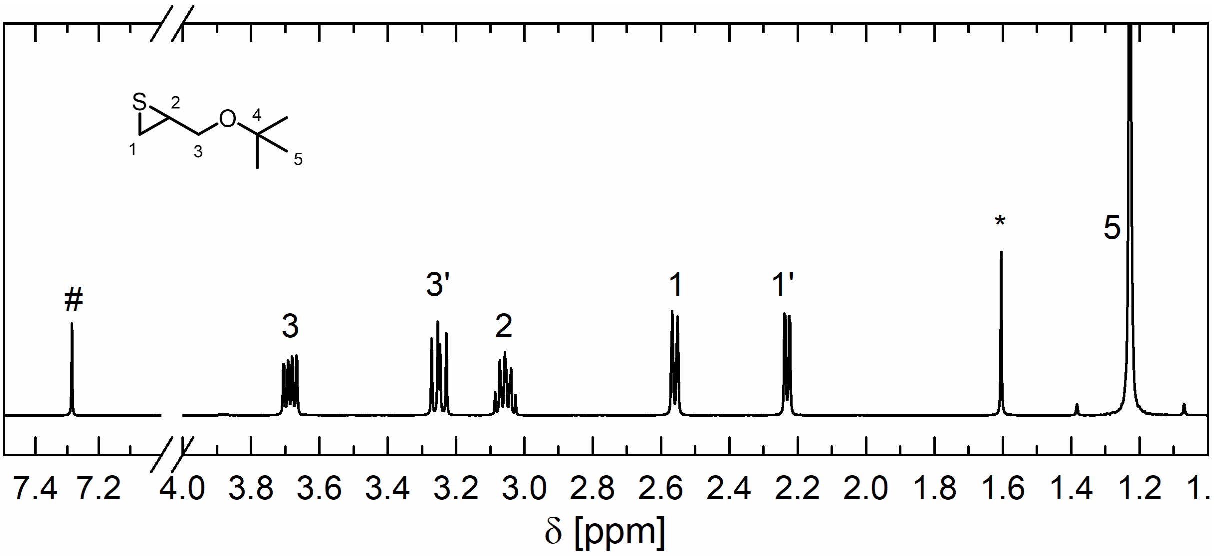

Figure S2. ^{1}H NMR spectrum of thiirane **2a**. Residual solvent peaks: chloroform-d (#), D_2O (*).

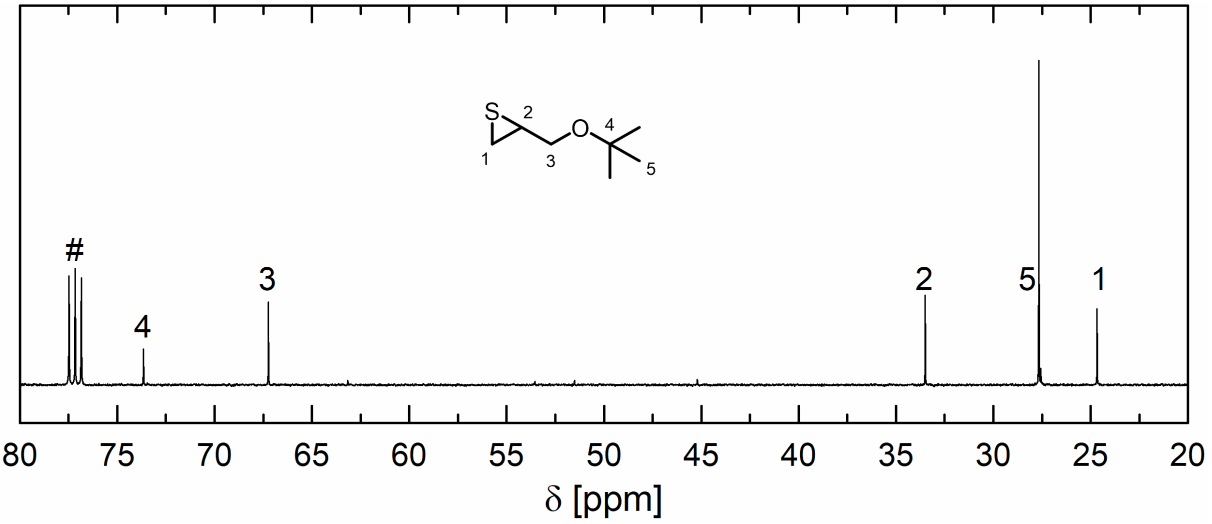

Figure S3. ^{13}C NMR spectrum of thiirane **2a**. Recorded in chloroform-d (#).

1.3.3 5-(hydroxymethyl)dihydrothiophen-2(3H)-iminium chloride (**3a**)

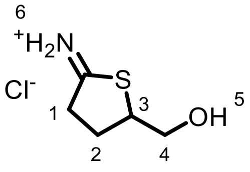

3a

Acetonitrile (0.75 mL, 14.4 mmol) was added dropwise to a stirred solution of *n*-BuLi (1.6 M in Hexane, 9.40 mL, 15.0 mmol) in dry THF (20 mL) at -78 °C under N_2. The reaction mixture was stirred at -78 °C for 15 min. Next, a solution of thiirane **2a** (2.00 g, 13.7 mmol) in dry THF (7.3 mL, c_{total} = 0.5 M) was slowly added (16 mL/h) to the white suspension. The reaction mixture was allowed to warm to -10 °C and stirred for 1 h. After evaporation of organic solvents under reduced pressure, the reaction mixture was quenched with a 1:1 EtOH-$HCl_{(conc)}$ mixture

(20 mL) and stirred at room temperature for 4 h. Again, the solvent was evaporated under reduced pressure to give iminium thiolactone **3a** (1.91 g, 83%) as an amber solid.

^{1}H NMR (400 MHz, DMSO-d_6) δ 12.20 (br. s, 2H, H6), 5.48 (br. S, 1H, H5), 4.20 (qd, J = 6.9, 5.2 Hz, 1H, H3), 3.75 (dd, J = 11.3, 5.1 Hz, 1H, H4), 3.57 (dd, J = 11.4, 7.3 Hz, 1H, H4), 3.36 – 3.18 (m, 2H, H1), 2.38 – 2.26 (m, 1H, H2), 2.15 – 2.03 (m, 1H, H2). IR (ATR) 3366, 2893, 2855, 1612, 1528,1485, 1432, 1407, 1317, 1276, 1256, 1103, 1060, 1039, 1019, 968, 928, 839, 684. HRMS (ESI) m/z for $C_5H_{10}NOS^+$ $(M)^+$ 132.0.

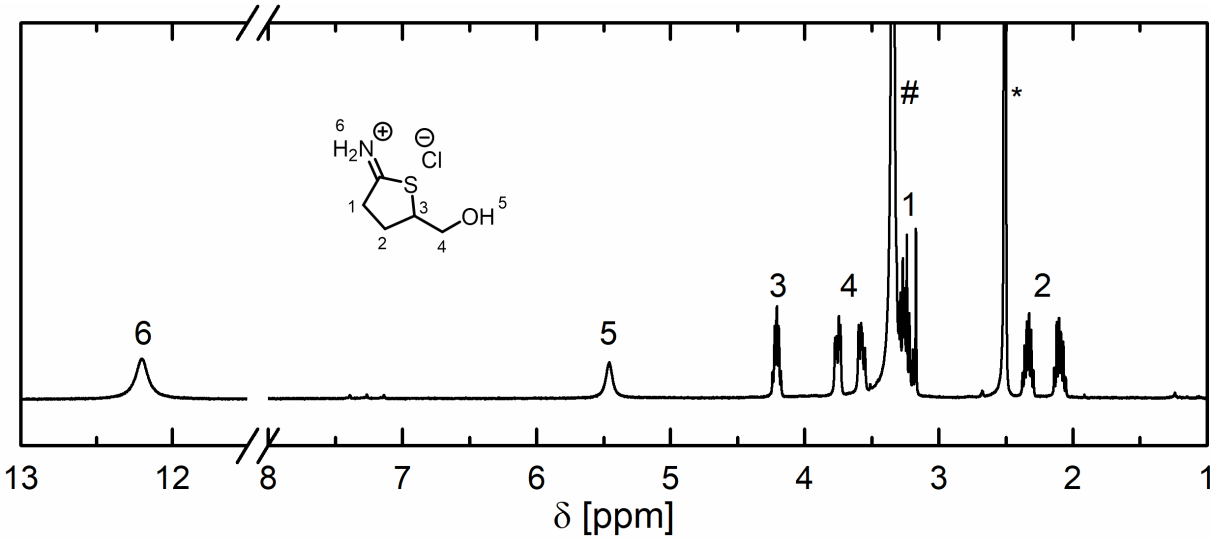

Figure S4. ^{1}H NMR spectrum of thiirane **3a**. Residual solvent peaks: DMSO-d_6 (*), D_2O (#).

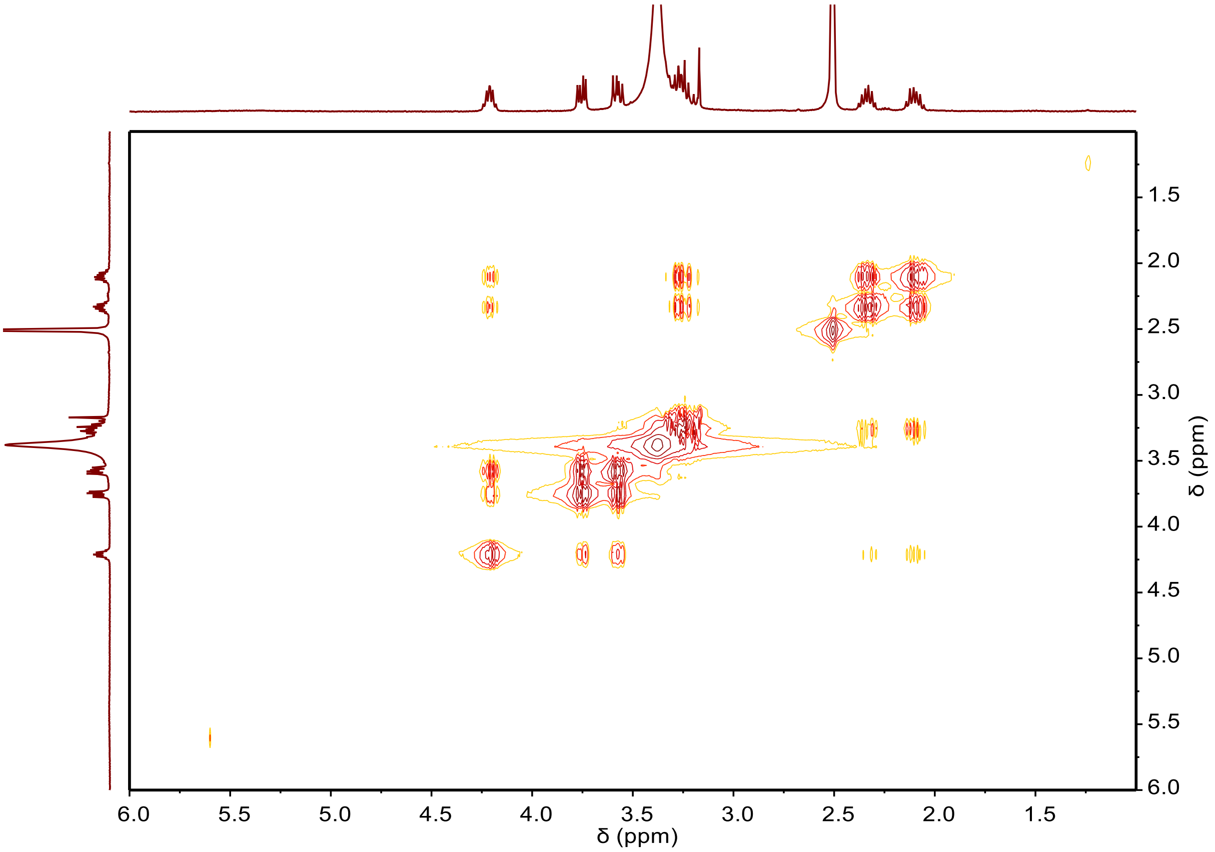

Figure S5. ^{1}H,^{1}H-COSY NMR spectrum of **3a**. Recorded in DMSO-d_6.

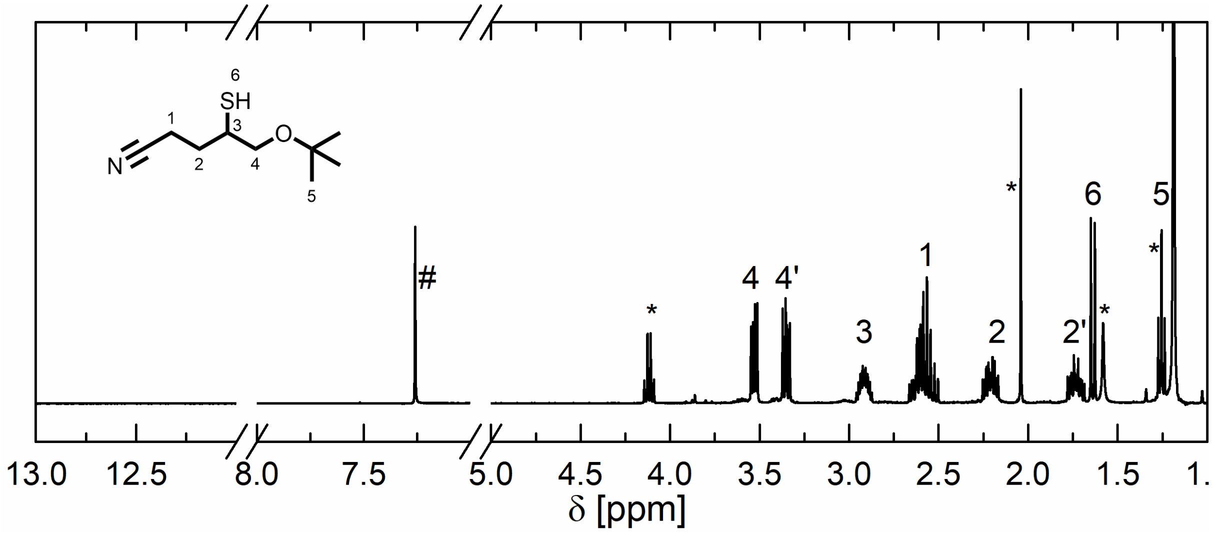

Figure S6. ^{1}H NMR spectrum of the thiol intermediate before its acidic cyclization to **3a**. Residual solvent peaks: chloroform-d (#), ethyl acetate (*). A small sample of the thiol intermediate was taken after the ring-opening reaction (prior the acidic quenching step) and extracted between $HCl_{(aq)}$ and ethyl acetate to confirm its structure.

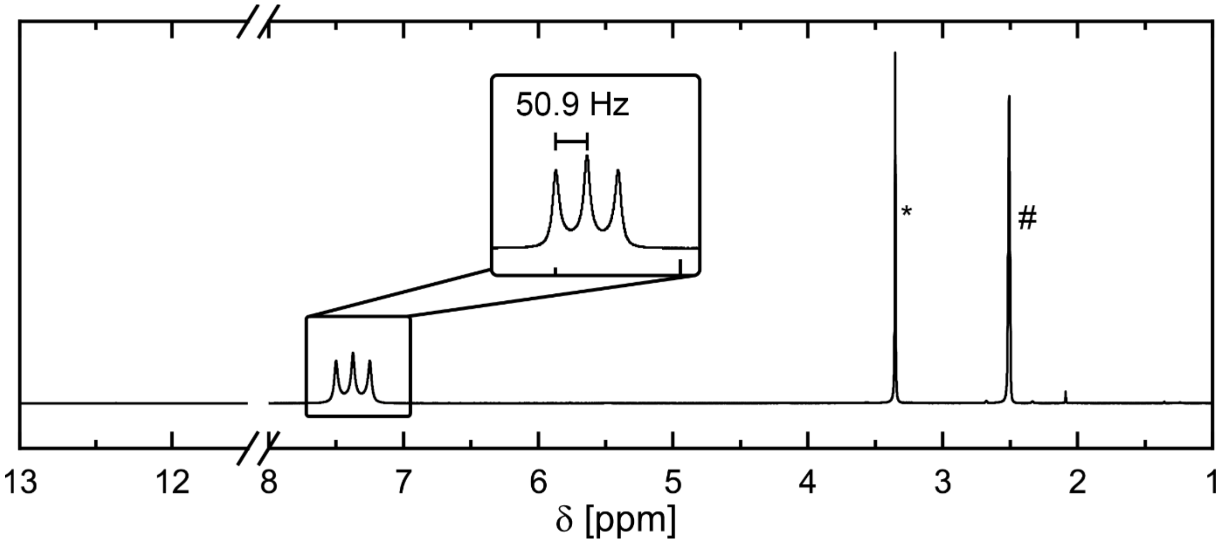

Figure S7. ^{1}H NMR spectrum of ammonium chloride, which precipitated during an attempt to recrystallize **3a**. Residual solvent peaks: DMSO-d_6 (#), H_2O (*). The spectrum shows a spin(1) triplet of the salt with a characteristic coupling constant $^{1}J_{14N\text{-}1H}$ = 50.9 Hz.

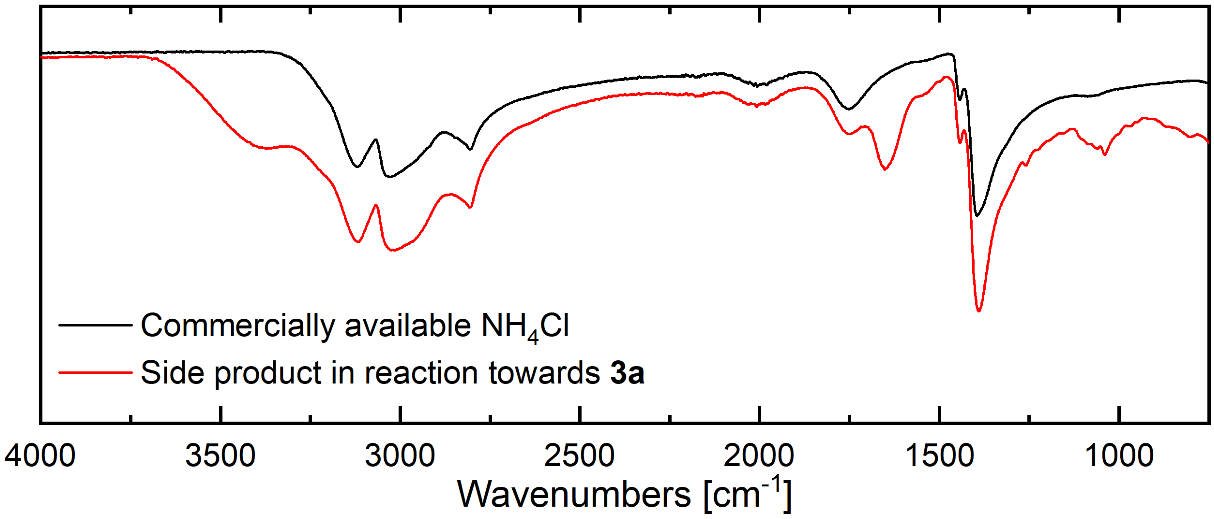

Figure S8. FTIR spectrum of commercially available ammonium chloride (black line) and the side product that can occur during synthesis of **3a** (red line). The two spectra largely overlap and show the same bands further providing evidence that ammonium chloride may be eliminated during the synthesis of iminiumthiolactone **3a**.

1.3.4 2-((allyloxy)methyl)thiirane (**2b**)

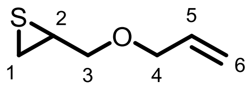

2b

Potassium thiocyanate (33.0 g, 340 mmol) was added to a stirred solution of allyl glycidyl ether **1b** (20.0 mL, 169 mmol) in 2,3-butanediol (34.0 mL, 5.0 M) at room temperature. The reaction mixture was stirred at room temperature for 8 h. Next, pentane (100 mL) was added, and the layers were separated. Then, water (100 mL) was added to the 2,3-butanediol layer and the mixture was extracted with pentane (2 x 100 mL). Combined organic layers were dried ($MgSO_4$), filtered and evaporated under reduced pressure to give thiirane **2b** (19.7 g, 86%) as a colorless liquid. Spectroscopic data was consistent with those reported in literature.[3]

^{1}H NMR (400 MHz, Chloroform-d) δ 5.92 (ddt, *J* = 17.2, 10.4, 5.7 Hz, 1H, H5), 5.29 (dq, *J* = 17.2, 1.6 Hz, 1H, H6), 5.20 (dq, *J* = 10.4, 1.6 Hz, 1H, H6), 4.05 (dt, *J* = 5.7, 1.5 Hz, 2H, H4), 3.65 (dd, *J* = 10.6, 5.7 Hz, 1H, H3), 3.45 (dd, *J* = 10.6, 6.8 Hz, 1H, H3), 3.09 (ddd, *J* = 12.1, 6.6, 5.6 Hz, 1H, H2), 2.53 (d, *J* = 6.2 Hz, 1H, H1), 2.22 (dd, J = 5.6, 1.3 Hz, 1H, H1).

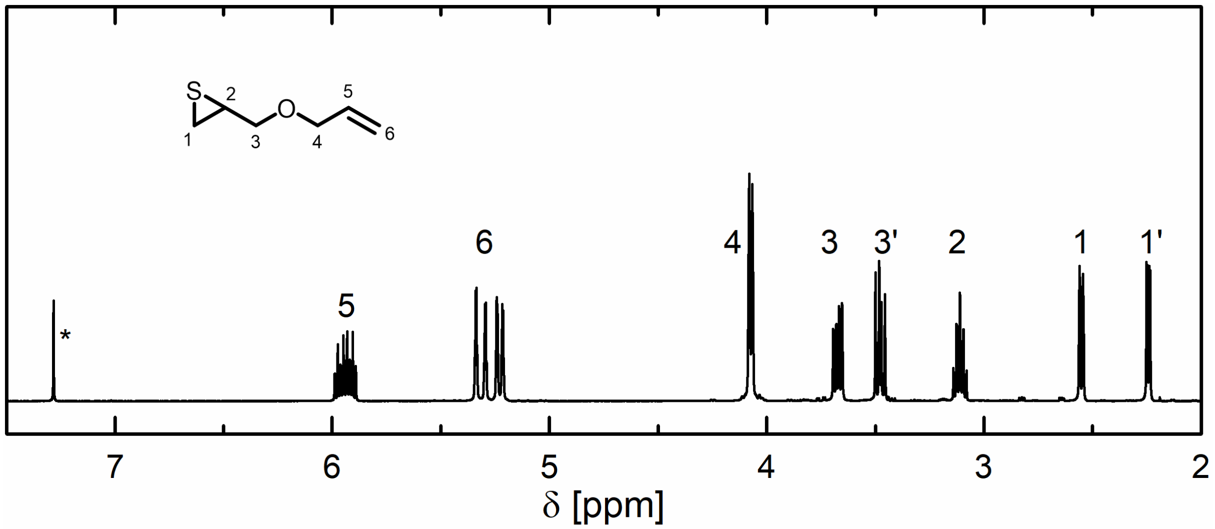

Figure S9. ^{1}H NMR spectrum of thiirane **2b**. Recorded in chloroform-d (*).

1.3.5 5-((allyloxy)methyl)dihydrothiophen-2(3H)-iminium chloride (**3b**)

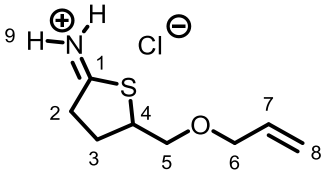

3b

Acetonitrile (0.75 mL, 14.4 mmol) was added dropwise to a stirred solution of *n*-BuLi (1.6 M in Hexane, 9.40 mL, 15.0 mmol) in dry THF (20 mL) at -78 °C under N_2. The reaction mixture was stirred at -78 °C for 15 min. Next, a solution of thiirane **2b** (1.78 g, 13.7 mmol) in dry THF (7.3 mL, c_{total} = 0.5 M) was slowly added (16 mL/h) to the white suspension. The reaction mixture was allowed to warm to -10 °C and stirred for 1 h. After evaporation of organic solvents under reduced pressure, the reaction mixture was quenched with a 1:1 EtOH-$HCl_{(conc)}$ mixture (20 mL) and stirred at room temperature for 4 h. Again, the solvent was evaporated under reduced pressure and the residue re-dissolved in dry acetonitrile (6.0 mL). The resulting suspension was centrifuged/filtered, and the residual solvent evaporated under reduced pressure to give iminium thiolactone **3b** (2.24 g, 79%) as an amber viscous liquid.

^{1}H NMR (500 MHz, D_2O) δ 5.96 (ddt, *J* = 17.3, 10.4, 5.9 Hz, 1H, H7), 5.35 (dq, *J* = 17.2, 1.6 Hz, 1H, H8), 5.30 (dq, *J* = 10.4, 1.3 Hz, 1H, H8), 4.51 – 4.41 (m, 1H, H4), 4.13 (dd, *J* = 5.9, 1.5 Hz, 2H, H6), 3.93 (dd, *J* = 10.8, 4.8 Hz, 1H, H5), 3.76 (dd, *J* = 10.8, 7.8 Hz, 1H, H5), 3.43 – 3.25 (m, 2H, H2), 2.61 – 2.51 (m, 1H, H3), 2.31 – 2.21 (m, 1H, H3). ^{13}C NMR (126 MHz, D_2O) δ 205.3 (C1), 133.5 (C7), 118.6 (C8), 72.1 (C6), 71.0 (C5), 55.8 (C4), 39.2 (C2), 29.5 (C3).

^{1}H NMR (400 MHz, DMSO-d_6) δ 12.54 (s, 2H, H9), 5.88 (ddd, *J* = 17.3, 10.5, 5.3 Hz, 1H, H7), 5.26 (dd, *J* = 17.3, 1.9 Hz, 1H, H8), 5.17 (dd, *J* = 10.4, 1.7 Hz, 1H, H8), 4.37 – 4.26 (m, 1H, H4), 4.01 (d, *J* = 5.4 Hz, 2H, H6), 3.76 (dd, *J* = 10.2, 4.9 Hz, 1H, H5), 3.57 (dd, *J* = 10.2, 7.8 Hz, 1H, H5), 3.36 – 3.16 (m, 2H, H2), 2.40 – 2.32 (m, 1H, H3), 2.13 – 2.01 (m, 1H, H3).

IR (ATR) 3395 (NH_2), 2852, 1627 (C=N), 1534, 1454, 1423, 1354, 1295, 1266, 1087, 994, 925, 879, 682. HRMS (ESI) *m/z* for $C_8H_{14}NOS^+$ $(M)^+$.

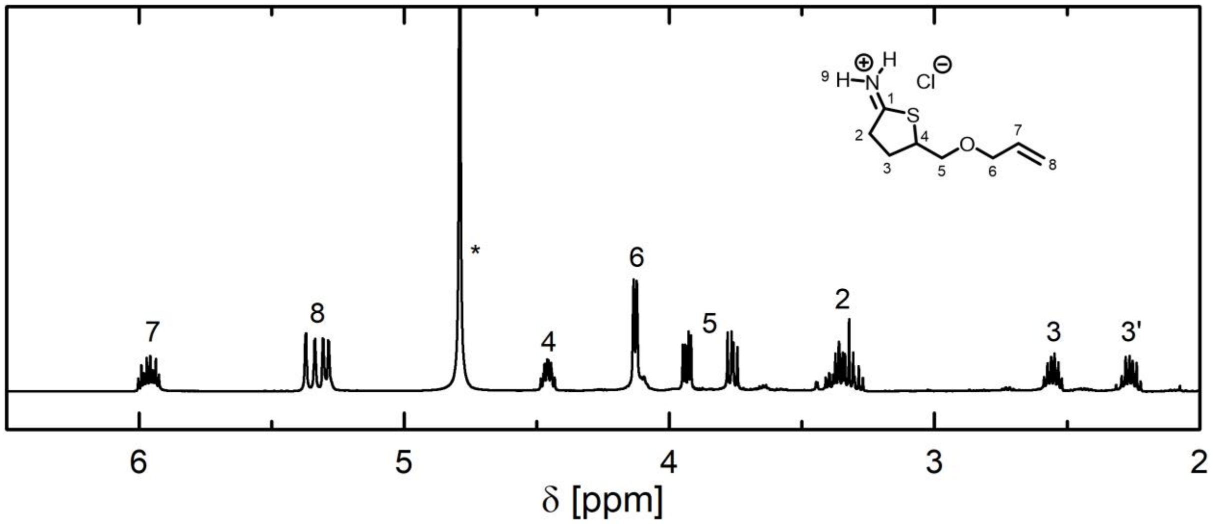

Figure S10 ^1H NMR spectrum of Iminium thiolactone **3b**. Residual solvent peaks: D_2O (*).

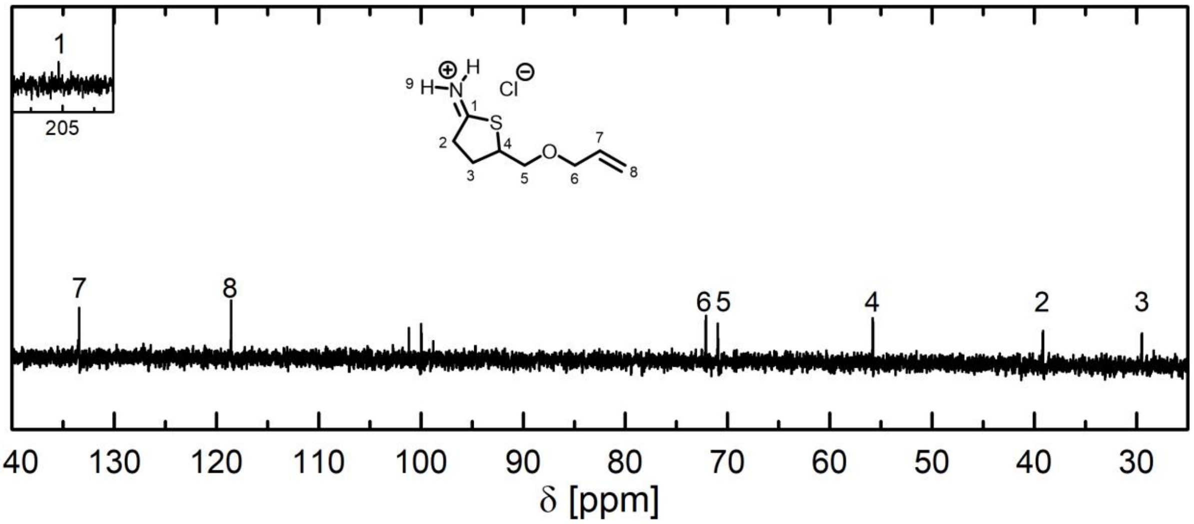

Figure S11 ^{13}C NMR spectrum of Iminium thiolactone **3b**. Recorded in D_2O.

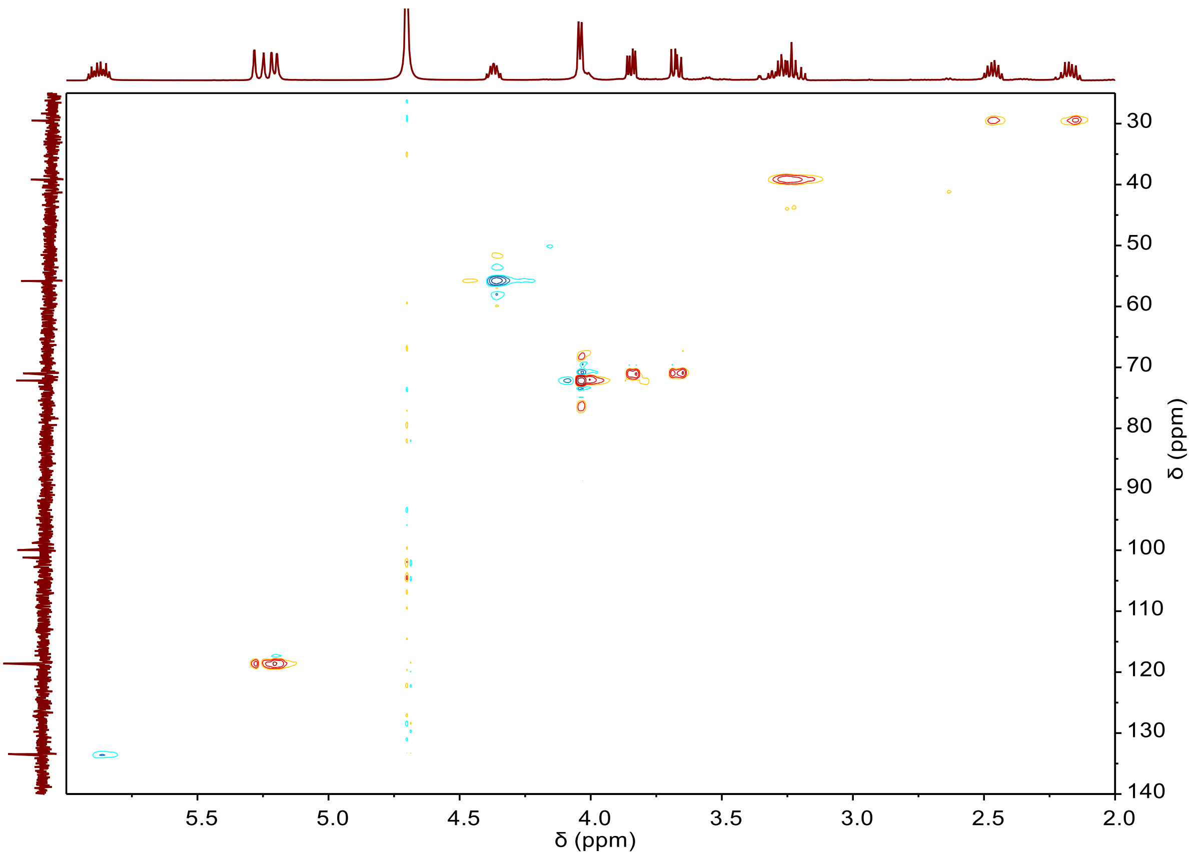

Figure S12. ^{1}H,^{13}C-HSQC NMR spectrum of **3a**. Recorded in D_2O.

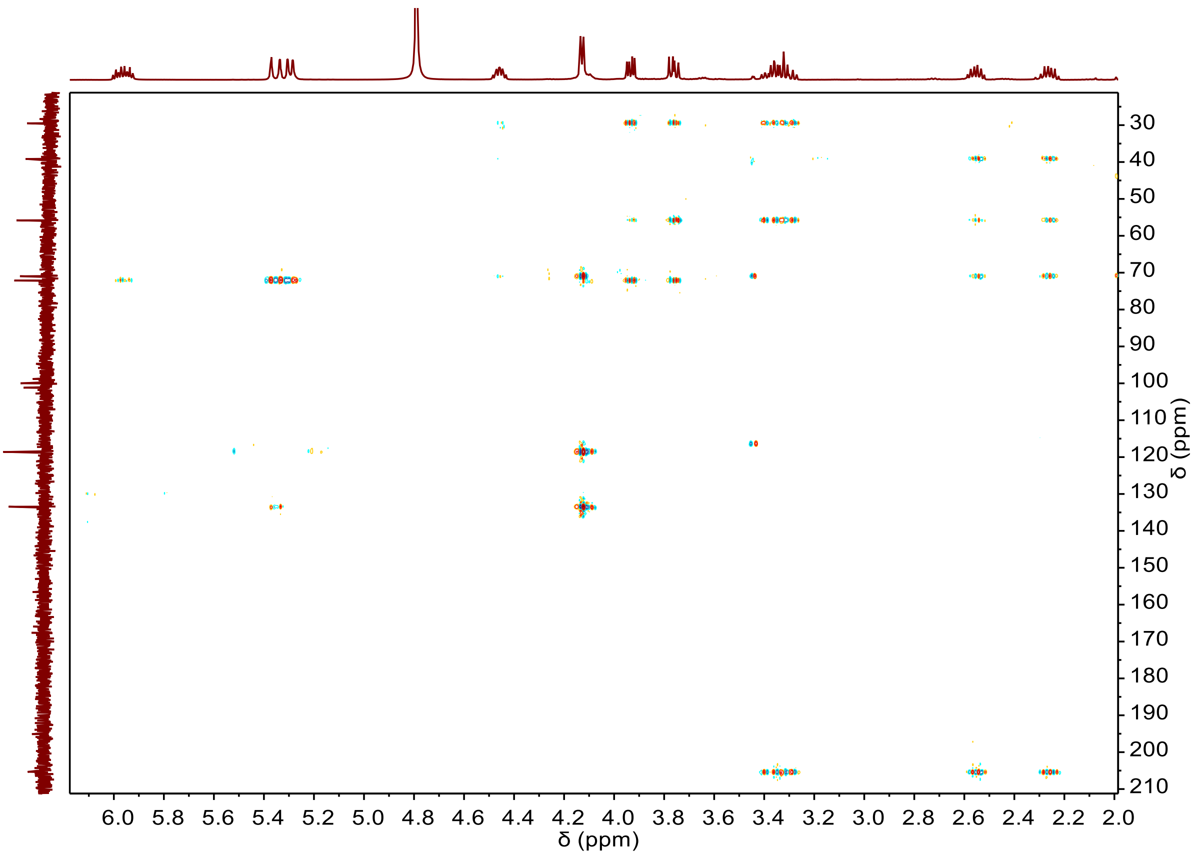

Figure S13. ^{1}H,^{13}C-HMBC NMR spectrum of **3a**. Recorded in D_2O.

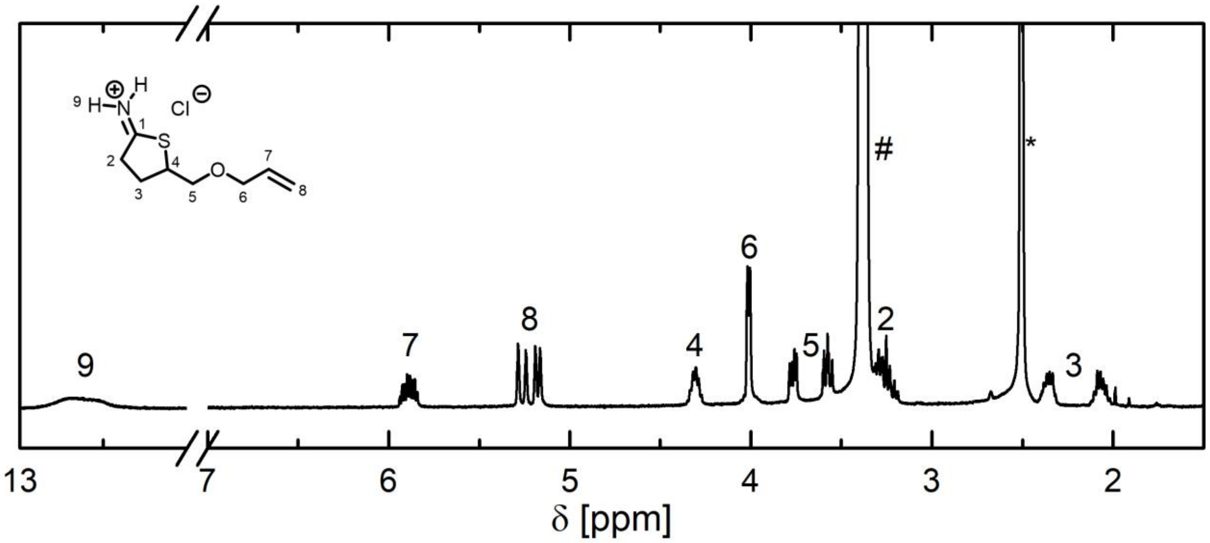

Figure S14. ^{1}H NMR spectrum of Iminium thiolactone **3b**. Residual solvent peaks: DMSO-d_6 (*), D_2O (#).

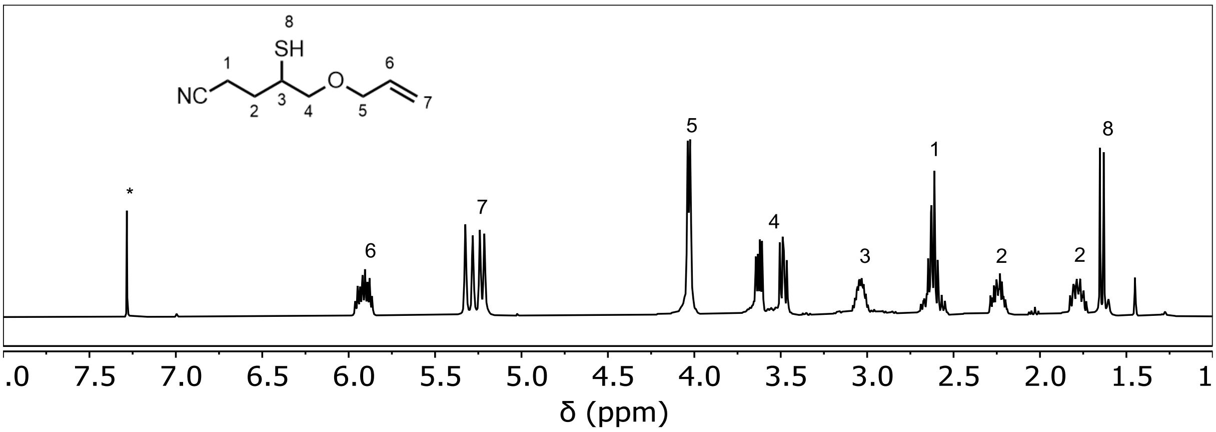

Figure S15. ^{1}H NMR spectrum of the thiol intermediate before its acidic cyclization to **3b**. Residual solvent peaks: chloroform-d (*). A small sample of the thiol intermediate was taken after the ring-opening reaction (prior the acidic quenching step) and extracted between $HCl_{(aq)}$ and ethyl acetate to confirm its structure.

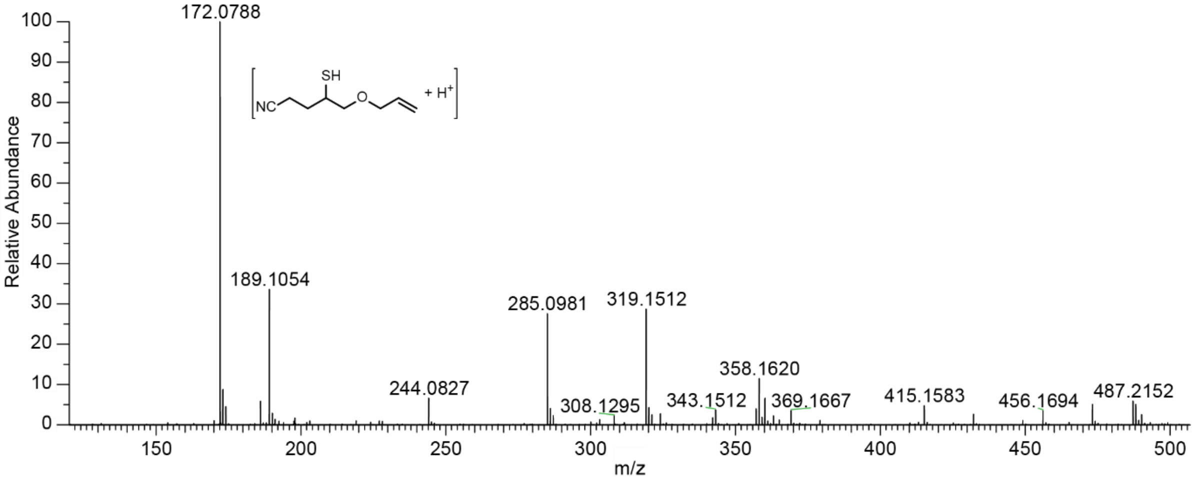

Figure S16. Mass spectrum of the thiol intermediate towards **3b**. Calculated m/z for $[M+H]^+$ = 172.08; found 172.0788.

1.3.6 5-((3-(ethylthio)propoxy)methyl)dihydrothiophen-2(3H)-iminium chloride (**3c**)

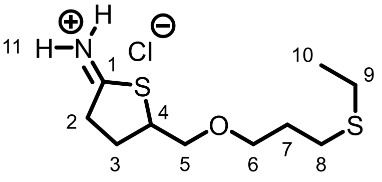

3c

Ethanethiol (296 µL, 4.00 mmol) and 2-hydroxy-4′-(2-hydroxyethoxy)-2-methylpropiophenone (18.0 mg, 0.08 mmol) were added to a filtered stirred solution of **3b** (208 mg, 1.00 mmol) in dry MeCN (1.0 mL, 1 M) at room temperature under N_2 in the dark. After degassing via three freeze-pump-thaw cycles, the reaction mixture was stirred at room temperature and under UV irradiation (350 nm) for 8 h. Next, the solvent was evaporated under reduced pressure and the crude product was separated between CH_2Cl_2 (1.0 mL) and water (1.0 mL). The aqueous layer was lyophilized to give thioether **3c** (41.4 mg, 15%) as a slightly yellow viscous oil. ^{1}H NMR (400 MHz, D_2O) δ 4.53 – 4.42 (m, 1H, H4), 3.92 (dd, *J* = 10.9, 4.7 Hz, 1H, H5), 3.75 (dd, *J* = 10.9, 7.8 Hz, 1H, H5), 3.70 (td, *J* = 6.2, 3.4 Hz, 2H, H6), 3.45 – 3.24 (m, 2H, H2), 2.66 (t, *J* = 7.2 Hz, 2H, H8), 2.63 – 2.48 (m, 3H, H9 and H3), 2.33 – 2.19 (m, 1H, H3), 1.94 – 1.85 (m, 2H, H7), 1.25 (t, *J* = 7.4 Hz, 3H, H10). ^{13}C NMR (101 MHz, D_2O) δ 205.4 (C1), 71.6 (C5), 69.8 (C6), 55.9 (C4), 39.2 (C2), 29.5 (C3), 28.4 (C7), 27.2 (C8), 25.2 (C9), 13.8 (C10). IR (ATR) 3387 (NH), 2929, 2871, 2812, 2156, 1704, 1607 ($C{=}NH_2$), 1536, 1454, 1423, 1375, 1264, 1116, 974, 879. HRMS (ESI) *m/z* for $C_{10}H_{20}NOS_2^+$ $(M + H)^+$ 234.0984.

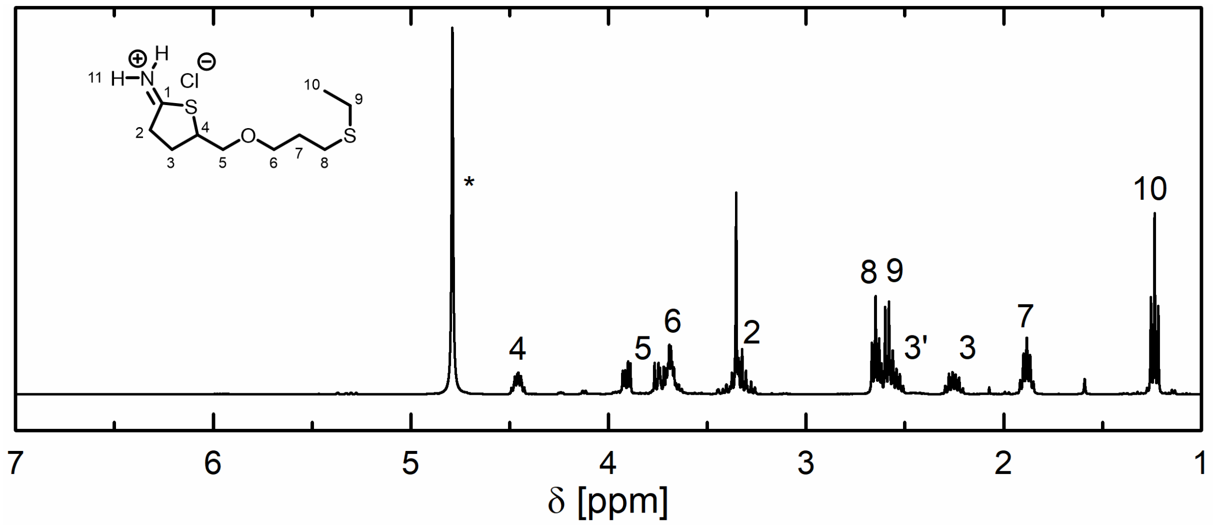

Figure S17. ^{1}H NMR spectrum of iminium thiolactone **3c**. Recorded in D_2O (*).

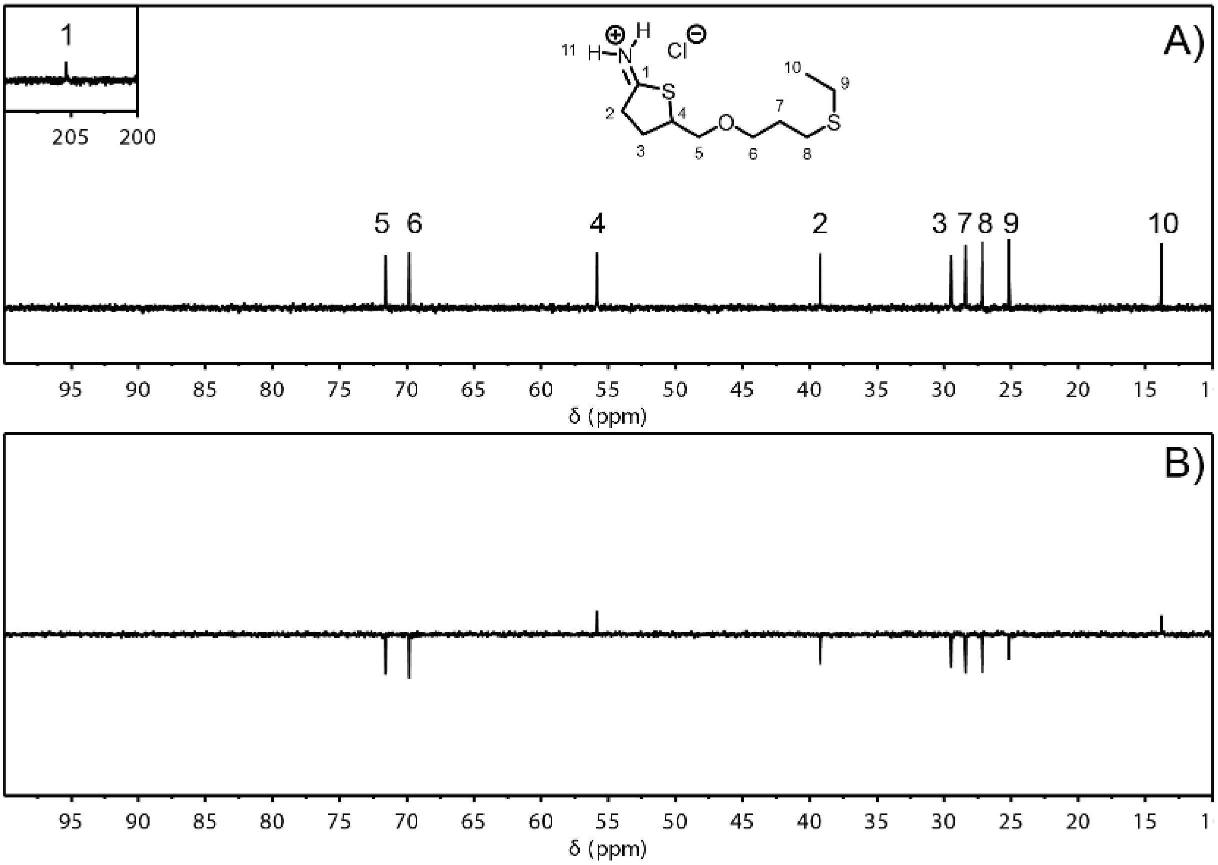

Figure S18. A) ^{13}C NMR spectrum and B) DEPT135 spectrum of iminium thiolactone **3c**. Recorded in D_2O.

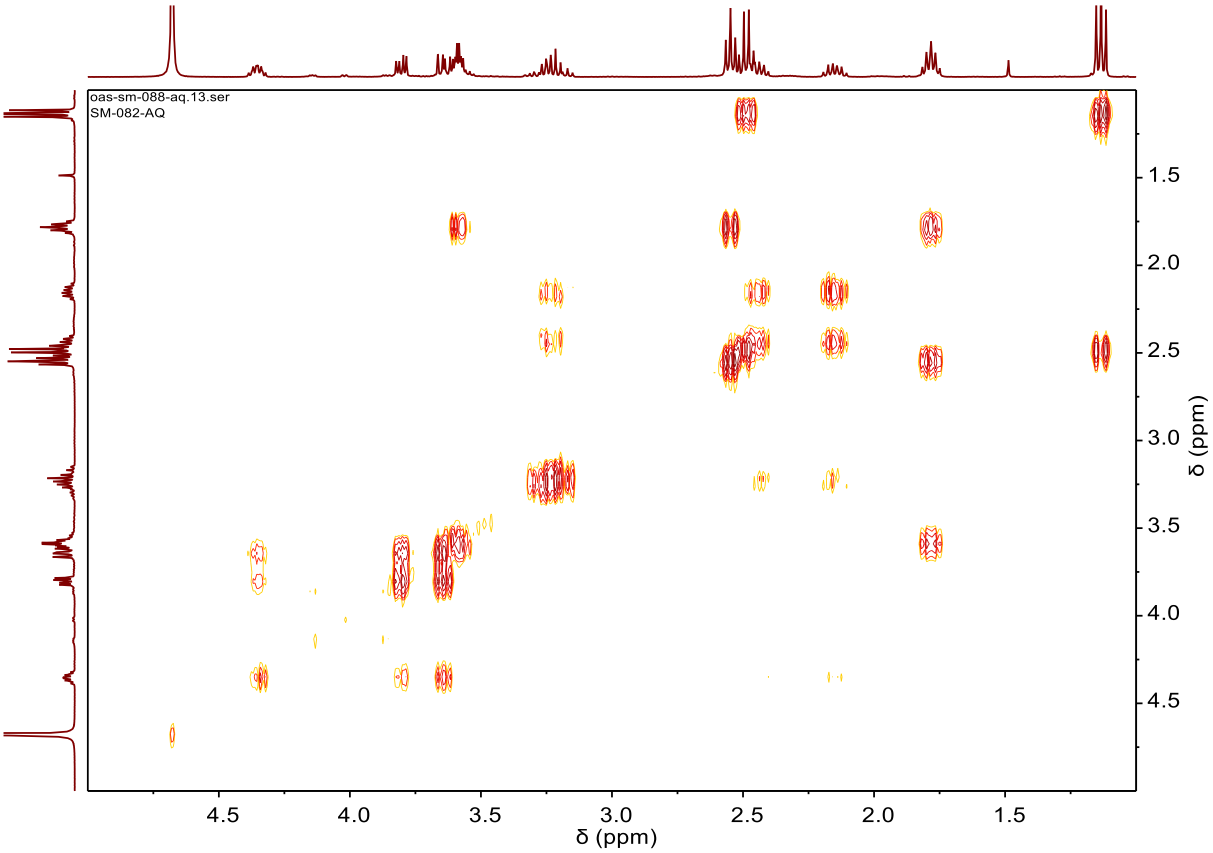

Figure S19. ^{1}H,^{1}H-COSY NMR spectrum of **3c**. Recorded in D_2O.

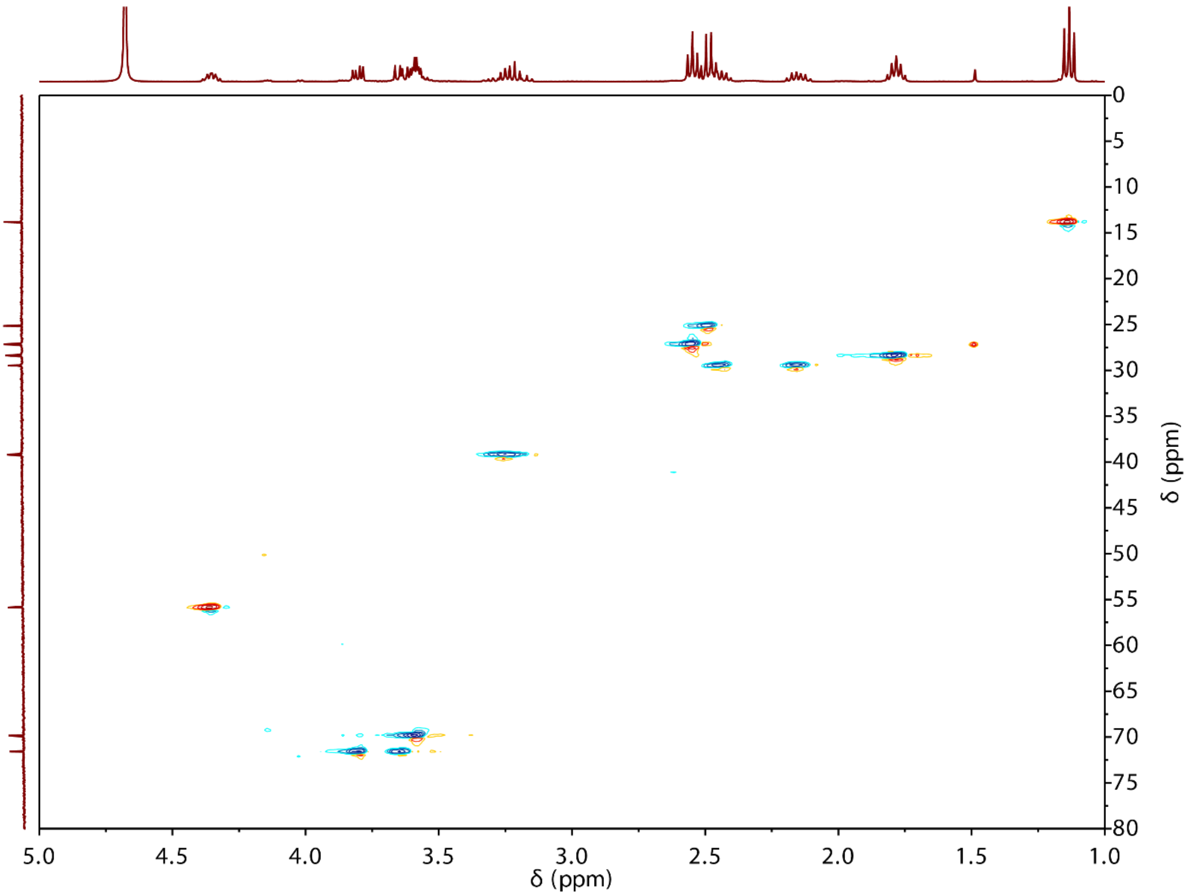

Figure S20. $^{1}H,^{13}C$-HSQC NMR spectrum of **3c**. Recorded in D_2O.

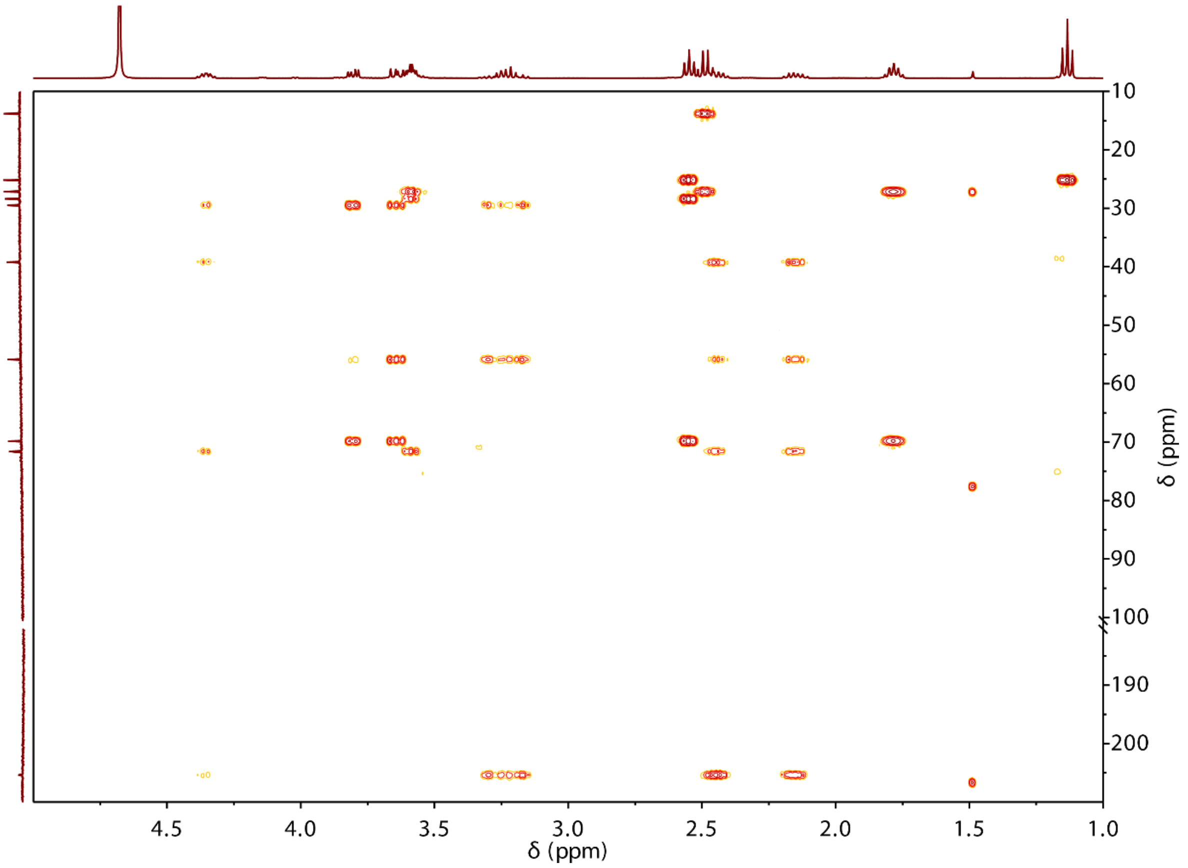

Figure S21. $^{1}H,^{13}C$-HMBC NMR spectrum of **3c**. Recorded in D_2O.

1.3.7 5-((3-((2-mercaptoethyl)thio)propoxy)methyl)dihydrothiophen-2(3H)-iminium chloride (**3d**)

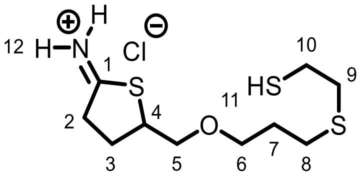

3d

2-hydroxy-2-methylpropiophenone (37.4 μL, 0.246 mmol) was added to a filtered stirred solution of **3b** (510 mg, 2.46 mmol) in 1,2-ethanedithiol (2.46 mL, 24.6 mmol, c = 1 M) at room temperature under N_2 in the dark. After degassing via bubbling with N_2 for 30 min, the reaction mixture was stirred at room temperature and under UV irradiation (350 nm) for 24 h. Next, 1,2-ethanedithiol was evaporated under reduced pressure and the crude product was separated between Et_2O (10 mL) and water (10 mL). The aqueous layer was extracted two more times with Et_2O (10 mL) before being lyophilized to give thiol **3d** (390 mg, 53%) as a slightly yellow viscous oil. ^{1}H NMR (500 MHz, D_2O) δ 4.50 – 4.43 (m, 1H, H4), 3.92 (dd, *J* = 10.8, 4.7 Hz, 1H, H5), 3.75 (dd, *J* = 10.9, 7.8 Hz, 1H, H5), 3.73 – 3.67 (m, 2H, H6), 3.43 – 3.26 (m, 2H, H2), 2.85 – 2.75 (m, 4H, H9/10), 2.67 (t, *J* = 7.2 Hz, 3H, H8/11), 2.60 – 2.51 (m, 1H, H3), 2.30 – 2.22 (m, 1H, H3), 1.89 (p, *J* = 7.2, 6.1 Hz, 2H, H7). ^{13}C NMR (126 MHz, D_2O) δ 205.3, 71.6, 69.7, 55.9, 39.2, 35.0, 29.5, 28.5, 27.5, 23.7. IR (ATR) 3388 (NH), 2921, 2867, 2547, 1699, 1630 ($C{=}NH_2$), 1535, 1417, 1372, 1294, 1266, 1211, 1115, 878, 683. HRMS (ESI) *m/z* for $C_{10}H_{20}NOS_3^+$ (M + H)$^+$ 266.0704.

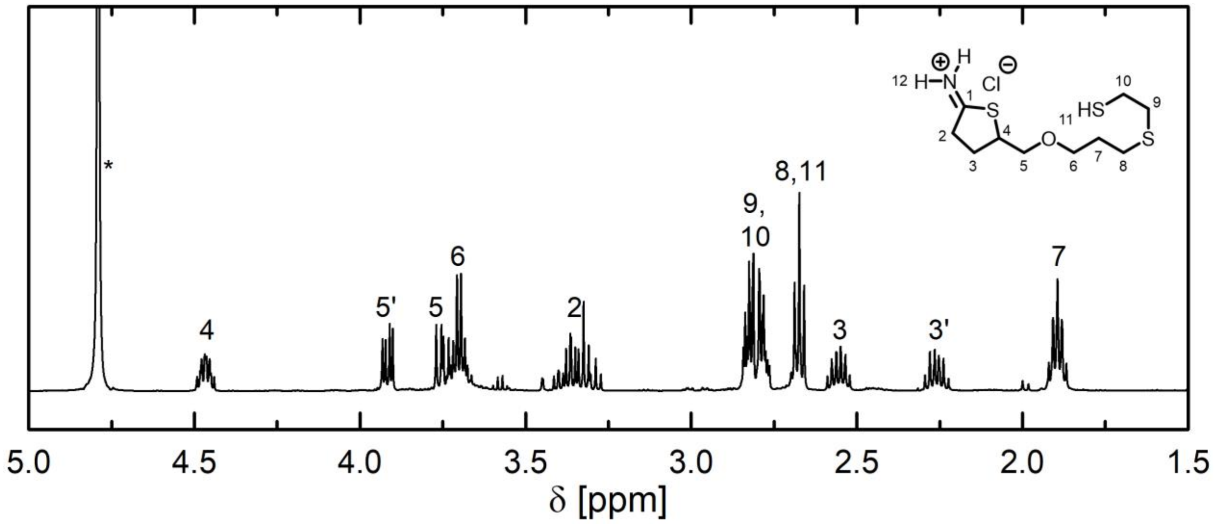

Figure S22. ^{1}H NMR spectrum of ITL Thiol **3d**. Recorded in D_2O (*).

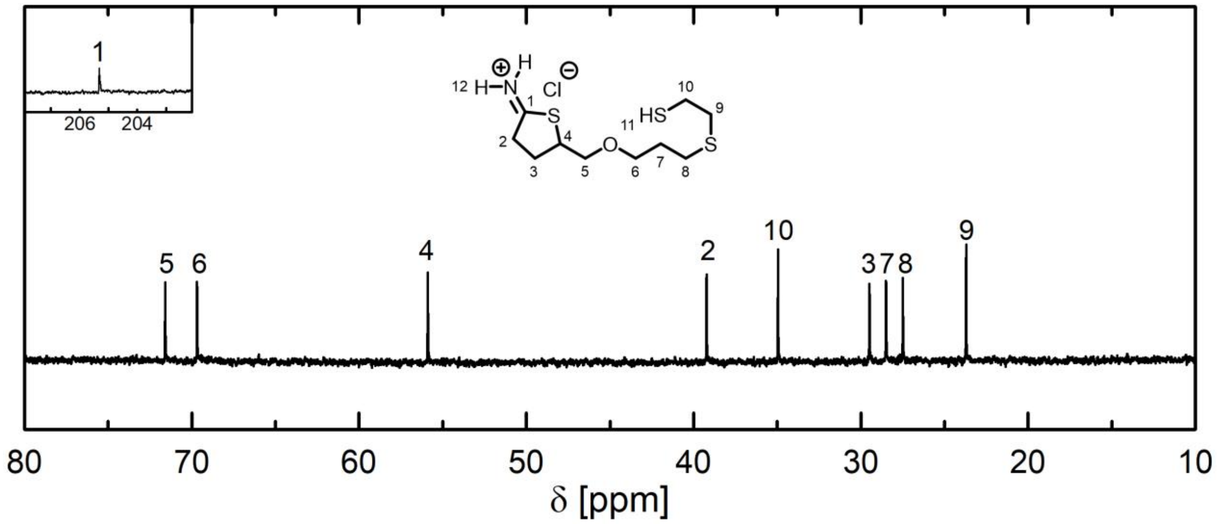

Figure S23. ^{13}C NMR spectrum of ITL Thiol **3d**. Recorded in D_2O.

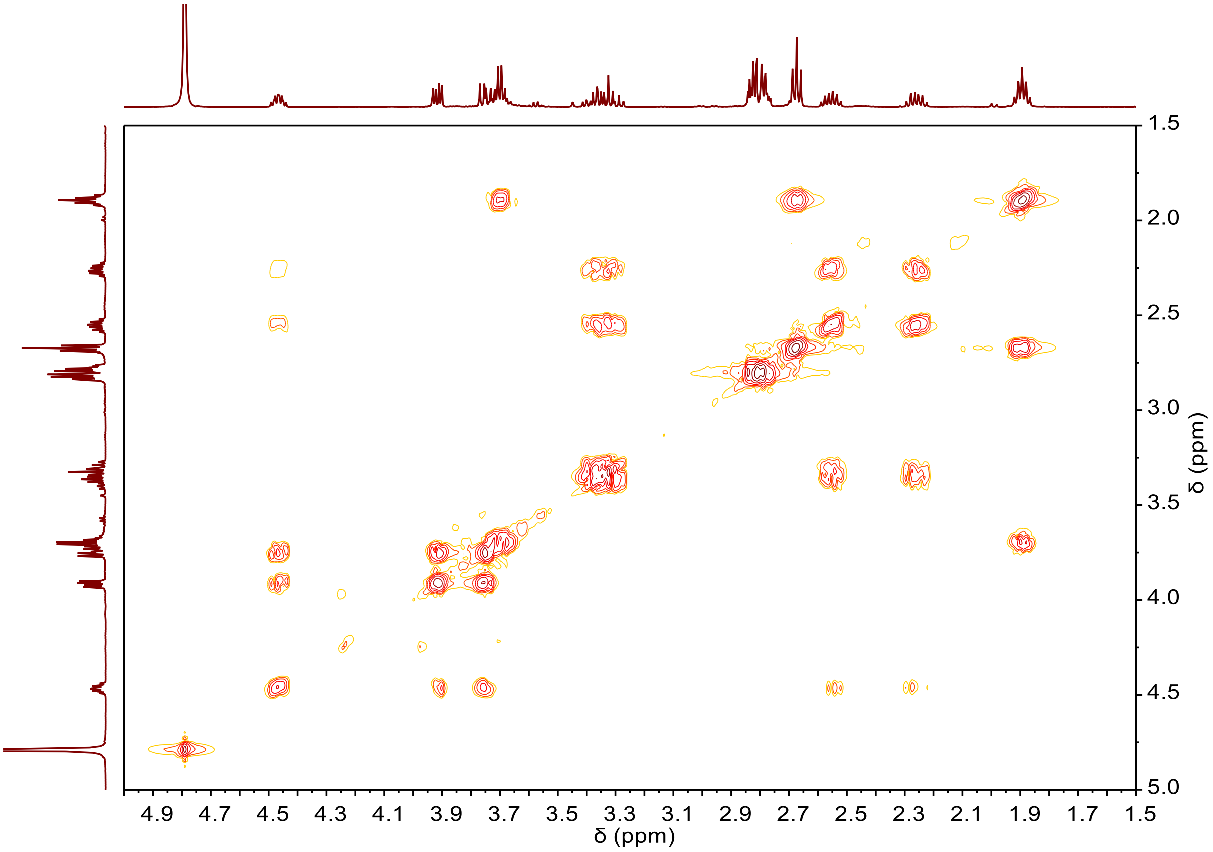

Figure S24. $^{1}H,^{1}H$-COSY NMR spectrum of **3d**. Recorded in D_2O.

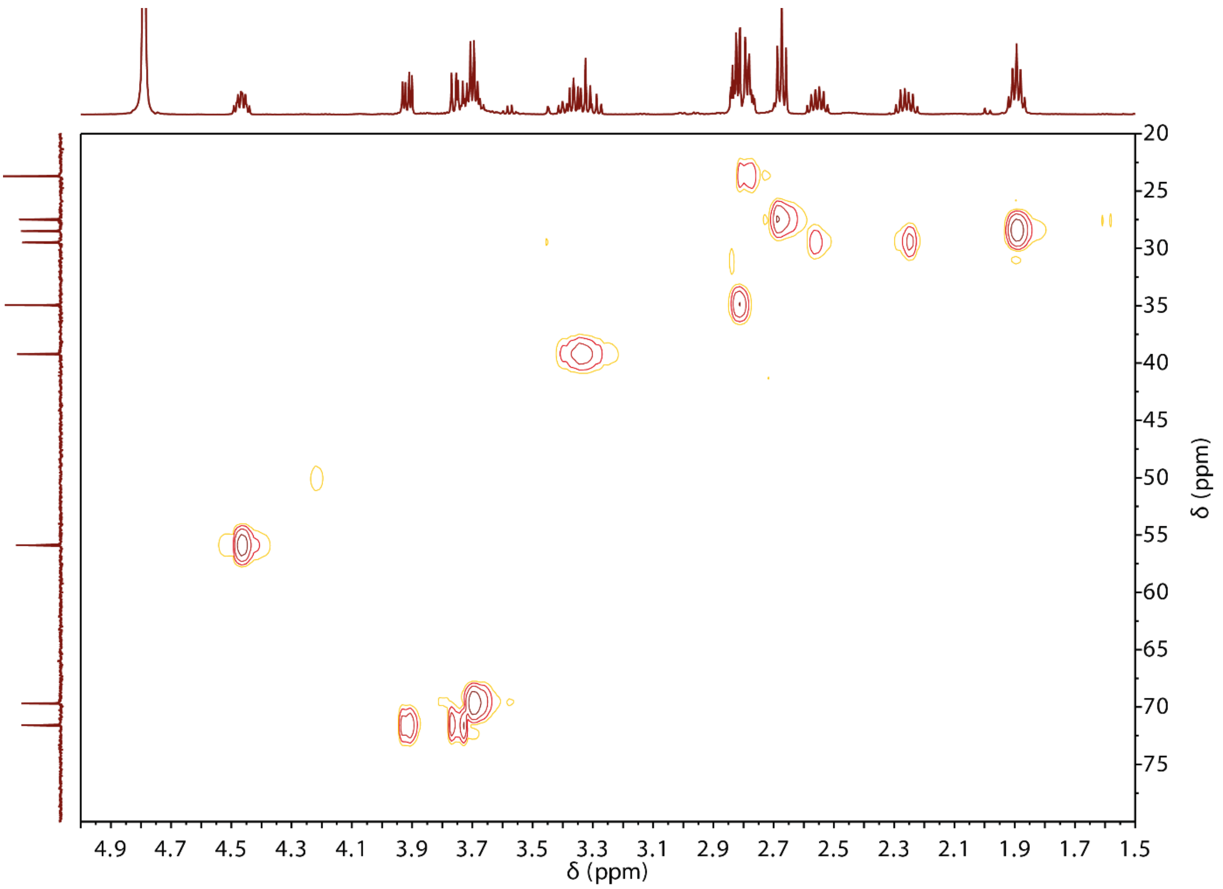

Figure S25. $^{1}H,^{13}C$-HSQC NMR spectrum of **3d**. Recorded in D_2O.

1.3.8 PEG(10)-ITL (**3e**)

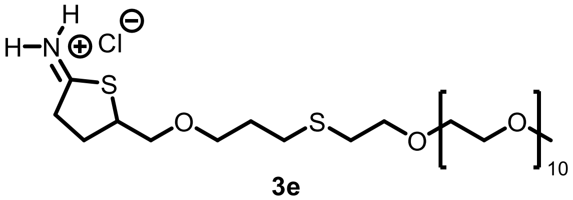

3e

2-hydroxy-4′-(2-hydroxyethoxy)-2-methylpropiophenone (2.04 mg, 9.09E-3 mmol) was added to a stirred bulk mixture of **3b** (28.2 mg, 0.136 mmol) and mPEG(10)-SH (50.0 mg, 9.09E-2 mmol) at room temperature under N_2 in the dark. The reaction mixture was stirred at room temperature and under UV irradiation (250 nm) for 24 h. The crude product was purified *via* semi-preparative HPLC using a MeOH/H_2O (+ 0.1% TFA) solvent mixture to afford a mixture of **3e** and residual mPEG(10)-SH as a viscous oil.

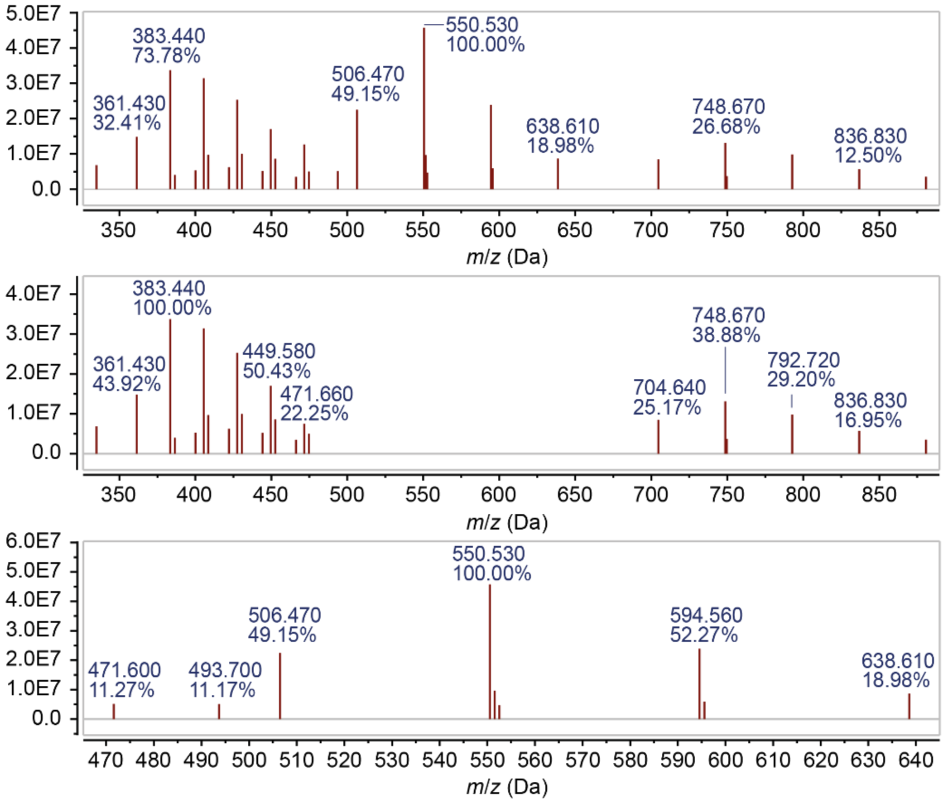

Figure S26. Mass spectrometry of the product mixture (top), PEG-ITL **3e** (middle) and residual mPEG(10)-SH (bottom).

Table S1. Mass spectrometry data of PEG-ITL **3e**. Reported peaks describe masses of $[M + H]^+$ (+ 1.007 g/mol).

N(PEG)	*m/z* (calculated)	*m/z* (found)	Intensity (a.u.)
10	704.410	704.640	8.505E6
11	748.440	748.670	1.310E7
12	792.470	792.720	9.800E6
13	836.500	836.830	5.611E6
14	880.530	880.800	3.526E6

Table S2. Mass spectrometry data of PEG-ITL **3e**. Reported peaks describe masses of $[M + H + NH_4]^{2+}$ (+ 19.046 g/mol).

N(PEG)	*m/z* (calculated)	*m/z* (found)	Intensity (a.u.)
10	361.215	361.430	1.478E7
11	383.230	383.440	3.366E7
12	405.245	405.490	3.135E7
13	427.260	427.530	2.530E7
14	449.275	449.580	1.698E7
15	471.290	471.636	1.259E7
16	493.305	493.700	0.513E7

Table S3. Mass spectrometry data of residual mPEG(10)-SH. Reported peaks describe masses of $[M + NH_4]^+$ (+ 18.039 g/mol).

N(PEG)	*m/z* (calculated)	*m/z* (found)	Intensity (a.u.)
9	506.330	506.470	2.215E7
10	550.360	550.530	4.579E7
11	594.390	594.560	2.402E7
12	636.420	638.610	8.581E6

1.3.9 (E)-2-acetamido-6-((5-((allyloxy)methyl)dihydrothiophen-2(3H)-ylidene)amino)-N-methylhexanamide (4)

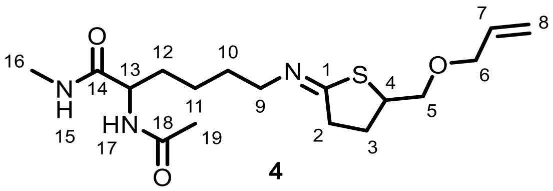

4

Nα-Acetyl-L-lysine methyl amide (55.2 mg, 0.27 mmol) was added to a stirred solution of **3b** (55.7 mg, 0.27 mmol) in PBS (5.4 mL, [**3b**] = 0.05 M) at room temperature. The reaction mixture was stirred at room temperature for 20 h. Next, the solvent was evaporated under reduced pressure to give the crude product. Purification by flash column chromatography on silica gel using CH_2Cl_2 / 10% MeOH as eluent gave product **4** (25.1 mg, 26%) as a white solid. R_f (9:1 CH_2Cl_2-MeOH) 0.50. Mp 130 °C. ^{1}H NMR (400 MHz, DMSO-d_6) δ 7.94 (d, *J* = 8.1 Hz, 1H, H17), 7.81 (q, *J* = 4.5 Hz, 1H, H15), 5.87 (ddt, *J* = 16.9, 10.5, 5.3 Hz, 1H, H7), 5.25 (dd, *J* = 17.3, 1.9 Hz, 1H, H8), 5.15 (dd, *J* = 10.5, 1.7 Hz, 1H, H8), 4.13 (td, *J* = 8.4, 5.4 Hz, 1H, H13), 3.98 (dd, *J* = 5.5, 1.5 Hz, 2H, H6), 3.96 – 3.86 (m, 1H, H4), 3.64 (dd, *J* = 9.8, 5.9 Hz, 1H, H5), 3.44 (dd, *J* = 9.8, 7.7 Hz, 1H, H5), 3.03 (t, *J* = 6.9 Hz, 2H, H9), 2.70 – 2.55 (m, 2H, H2), 2.55 (d, *J* = 4.6 Hz, 3H, H16), 2.16 – 2.06 (m, 1H, H3), 1.83 (s, 3H, H19), 1.80 – 1.69 (m, 1H, H3), 1.68 – 1.41 (m, 4H, H10/12), 1.38 – 1.19 (m, 2H, H11). ^{13}C NMR (101 MHz, DMSO-d_6) δ 172.2 (C14), 169.9 (C1), 169.2 (C18), 135.0 (C7), 116.6 (C8), 72.7 (C5), 71.1 (C6), 56.8 (C9), 52.6 (C13), 49.2 (C4), 37.2 (C2), 32.0 (C12), 29.7 (C10), 28.6 (C3), 25.5 (C16), 23.6 (C11), 22.6 (C19). IR (ATR) 3292 (NH), 3118, 2925, 2864, 1633 (C=N), 1548, 1454, 1426, 1377, 1356, 1285, 1252, 1097, 993, 944, 812, 787, 720. HRMS (ESI) *m/z* for $C_{17}H_{29}N_3O_3S$ $(M + H)^+$ 356.2004.

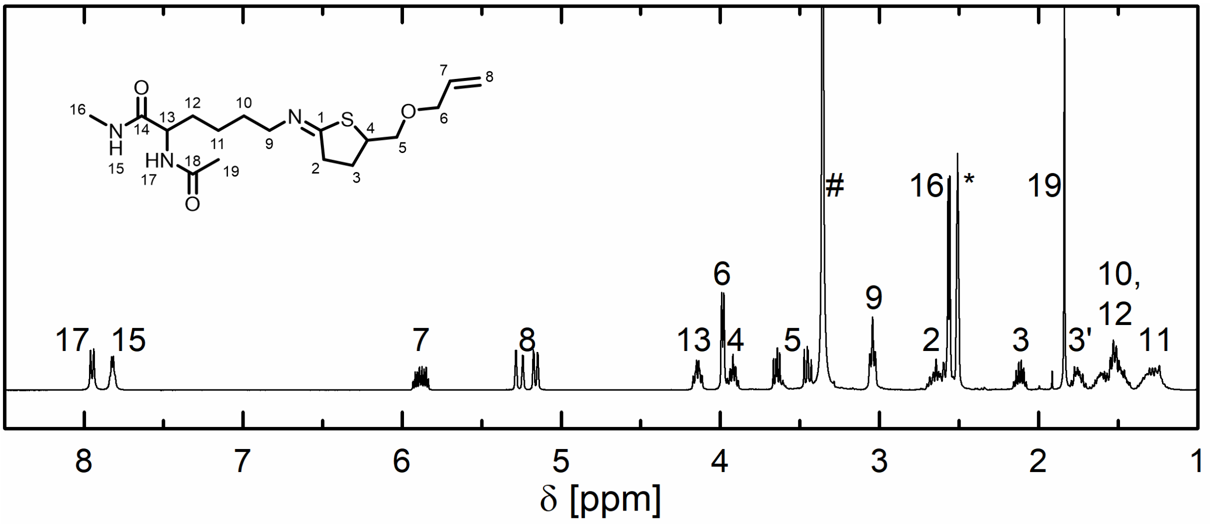

Figure S27. ^{1}H NMR spectrum of adduct **4**. Residual solvent peaks: DMSO-d_6 (*), D_2O (#).

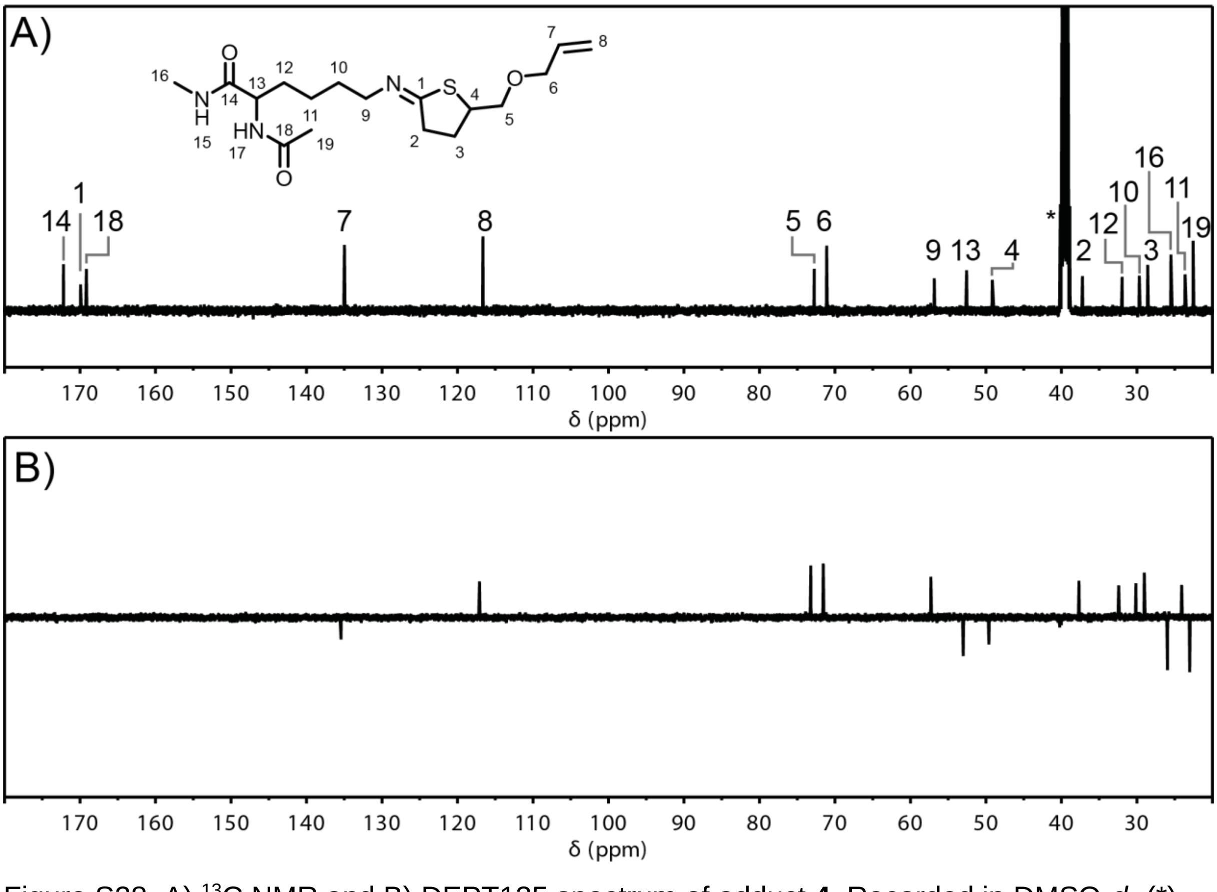

Figure S28. A) ^{13}C NMR and B) DEPT135 spectrum of adduct **4**. Recorded in DMSO-d_6 (*).

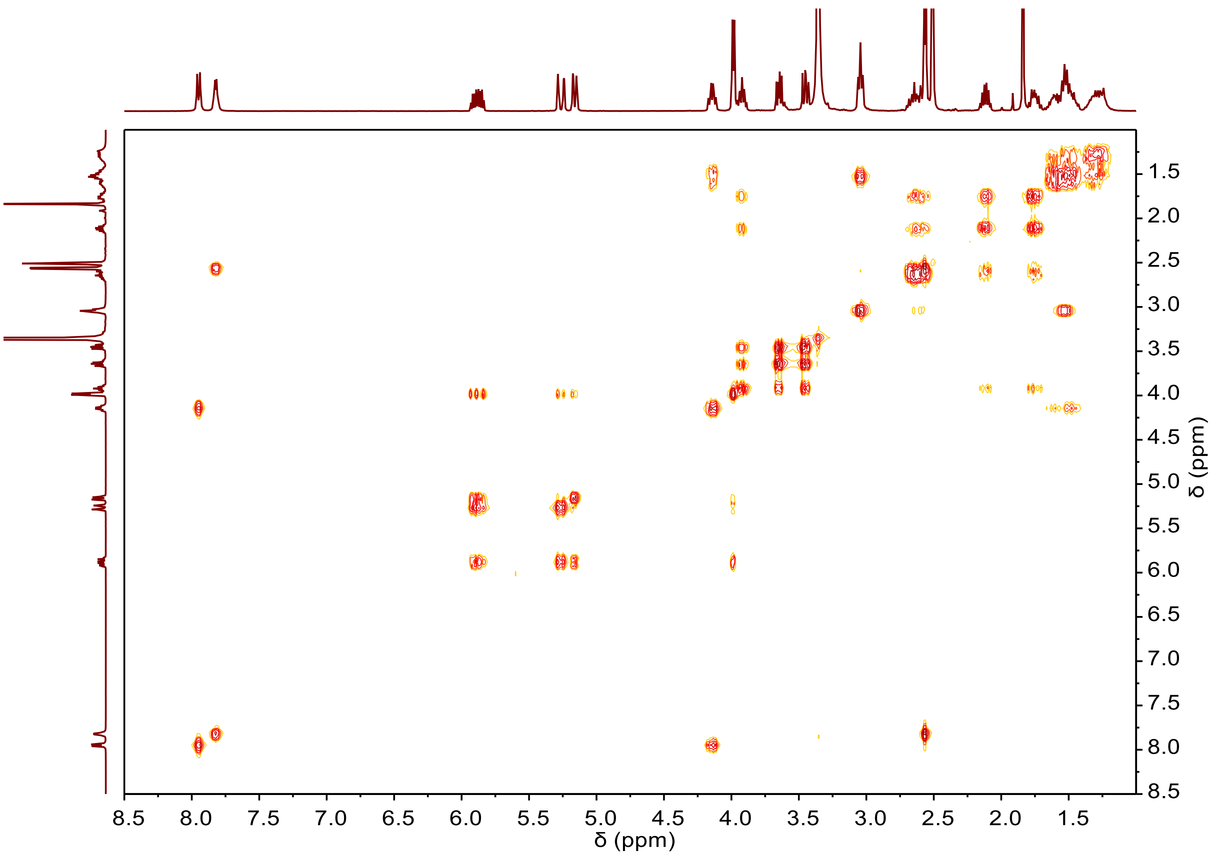

Figure S29. $^{1}H,^{1}H$-COSY NMR spectrum of **4**. Recorded in DMSO-d_6.

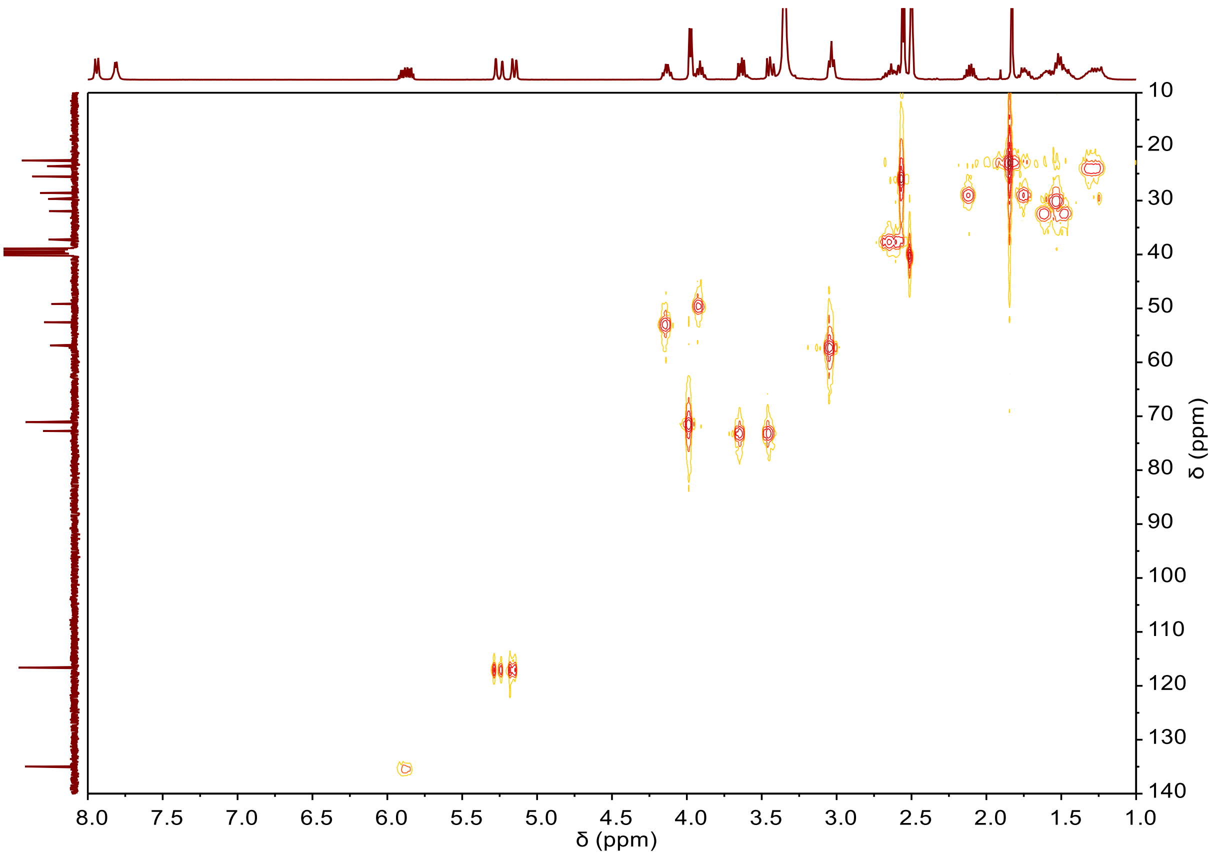

Figure S30. $^{1}H,^{13}C$-HSQC NMR spectrum of **4**. Recorded in DMSO-d_6.

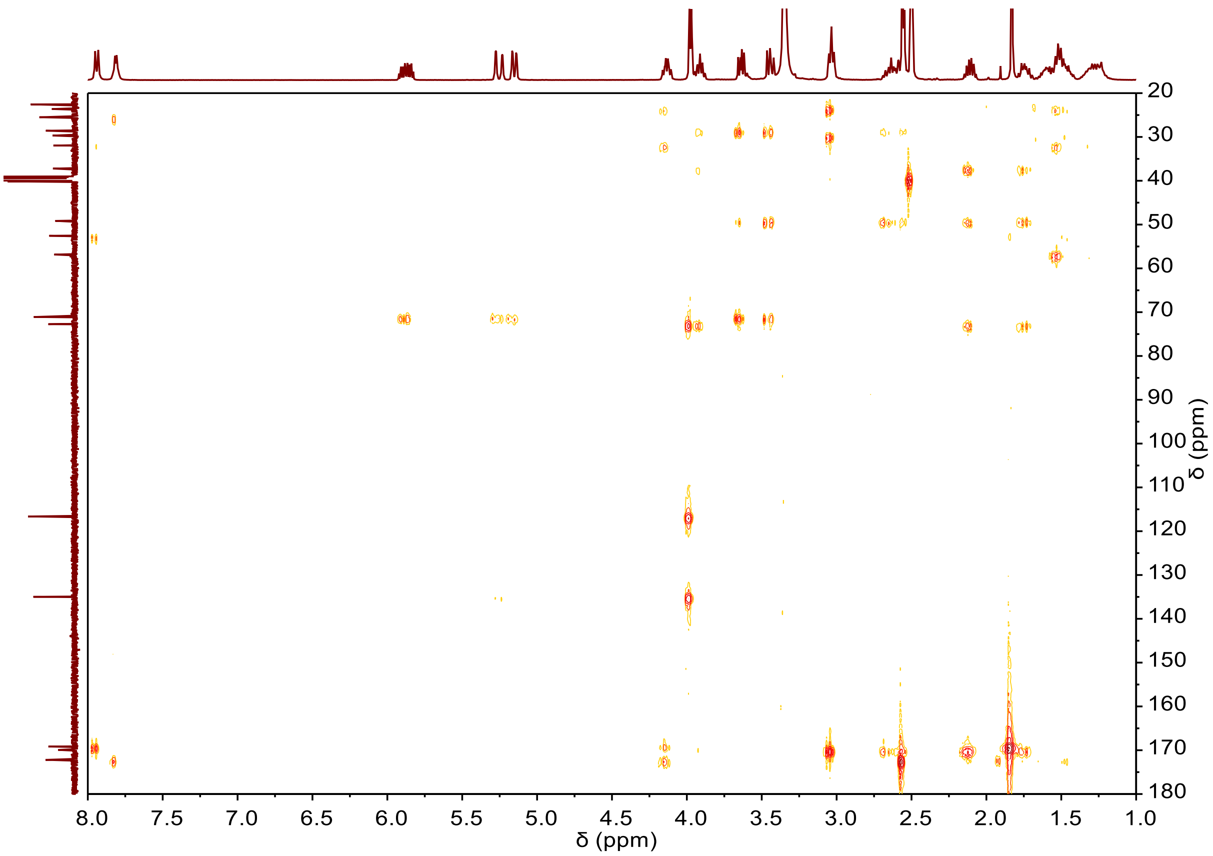

Figure S31. ^{1}H,^{13}C-HMBC NMR spectrum of **4**. Recorded in DMSO-d_6.

1.3.10 1-((5-acetamido-6-(methylamino)-6-oxohexyl)amino)-5-(allyloxy)-4-((1-methyl-2,5-dioxopyrrolidin-3-yl)thio)pentan-1-iminium (**4***)

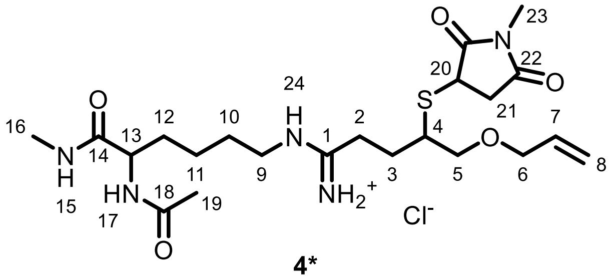

4*

A solution of N$_\alpha$-acetyl-L-lysine methyl-N-amide (**K'**) (96.9 mg, 0.481 mmol) in PBS (0.814 mL, 10 mM, pH = 7.4) was added to a filtered (0.2 µm, Nylon) and stirred solution of **3b** (100 mg, 0.481 mmol) and N-methyl maleimide (NMM) (58.8 mg, 0.530 mmol) in PBS (4.00 mL, [**3b**]$_{TOTAL}$ = 0.01 M) at room temperature. The reaction mixture was stirred at room temperature for 2 h. Purification by semi-preparative HPLC using a gradient of 10% to 75% MeOH in H_2O (+ 0.1% TFA) over 20 min gave product **4*** (76.0 mg, 30%) as a waxy solid. ^{1}H NMR (500 MHz, DMSO-d_6) δ 9.42 – 9.27 (m, 1H, H24), 9.04 – 8.97 (m, 1H, H25), 8.60 – 8.52 (m, 1H, H25), 7.98 (d, *J* = 8.1 Hz, 1H, H17), 7.85 (d, *J* = 4.7 Hz, 1H, H15), 5.94 – 5.84 (m, 1H, H7), 5.31 – 5.22 (m, 1H, H8), 5.21 – 5.12 (m, 1H, H8'), 4.16 (td, *J* = 8.5, 5.5 Hz, 1H, H13), 4.12 – 4.04 (m, 1H, H20), 4.02 – 3.94 (m, 2H, H6), 3.67 – 3.58, 3.53 – 3.43 (m, 2H, H5), 3.40 – 3.28, 3.21 – 3.08 (m, 1H, H4), 3.23 – 3.14 (m, 1H, H21), 3.14 (s, 2H, H9), 2.84 (s, 3H, H23), 2.56 (d, *J* = 4.5 Hz, 3H,

H16), 2.62 – 2.40 (m, 2H, H2), 2.57 – 2.50, 2.48 – 2.40 (m, 1H, H21), 2.13 – 1.96, 1.82 – 1.74, 1.72 – 1.61 (m, 2H, H3), 1.84 (s, 3H, H19), 1.71 – 1.58 (m, 1H, H12), 1.58 – 1.38 (m, 3H, H12 and H10), 1.37 – 1.16 (m, 2H, H11k). ^{13}C NMR (126 MHz, DMSO-d_6) δ 177.9, 177.7, 177.3, 177.2, 175.7, 175.6, 175.5 (C22), 172.5 (C18), 169.7 (C14), 167.3, 167.2, 167.0 (C1), 135.4, 135.3 (C7), 117.2 (C8), 73.3, 72.9 (C5), 71.6, 71.6 (C6), 64.5, 64.4 (C5), 52.7 (C13), 47.4, 47.1, 44.9, 44.5 (C4), 42.2 (C9), 39.0, 38.9 (C20), 37.4, 37.2, 36.5, 36.4 (C21), 32.0 (C12), 30.8, 30.7 (C2), 29.6, 29.1, 28.8 (C3), 27.2 (C10), 26.0 (C16), 25.2 (C23), 23.1, 23.0 (C11 and C19). HRMS (ESI) *m/z* for $C_{22}H_{38}N_5O_5S^+$ $(M)^+$ 484.3771.

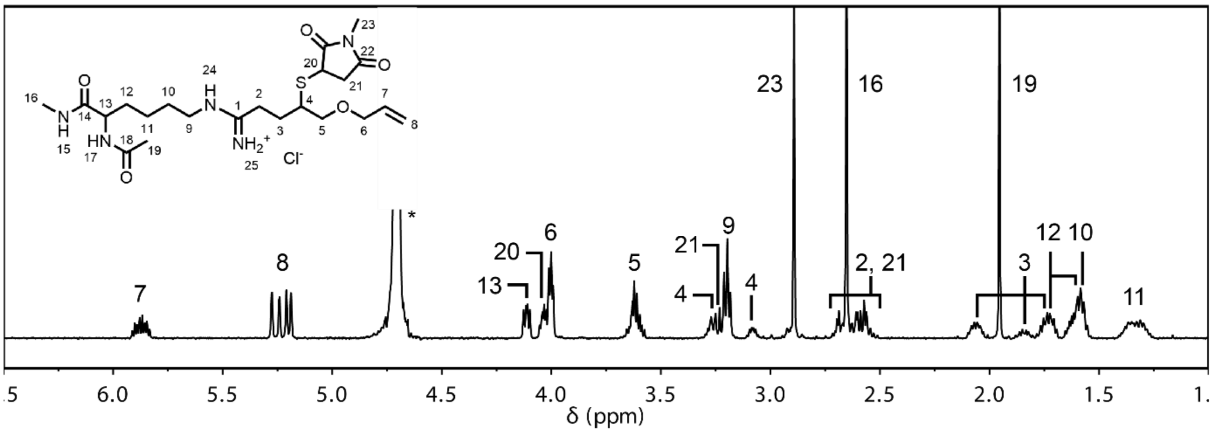

Figure S32. ^{1}H NMR spectrum of **4***. Recorded in D_2O (*).

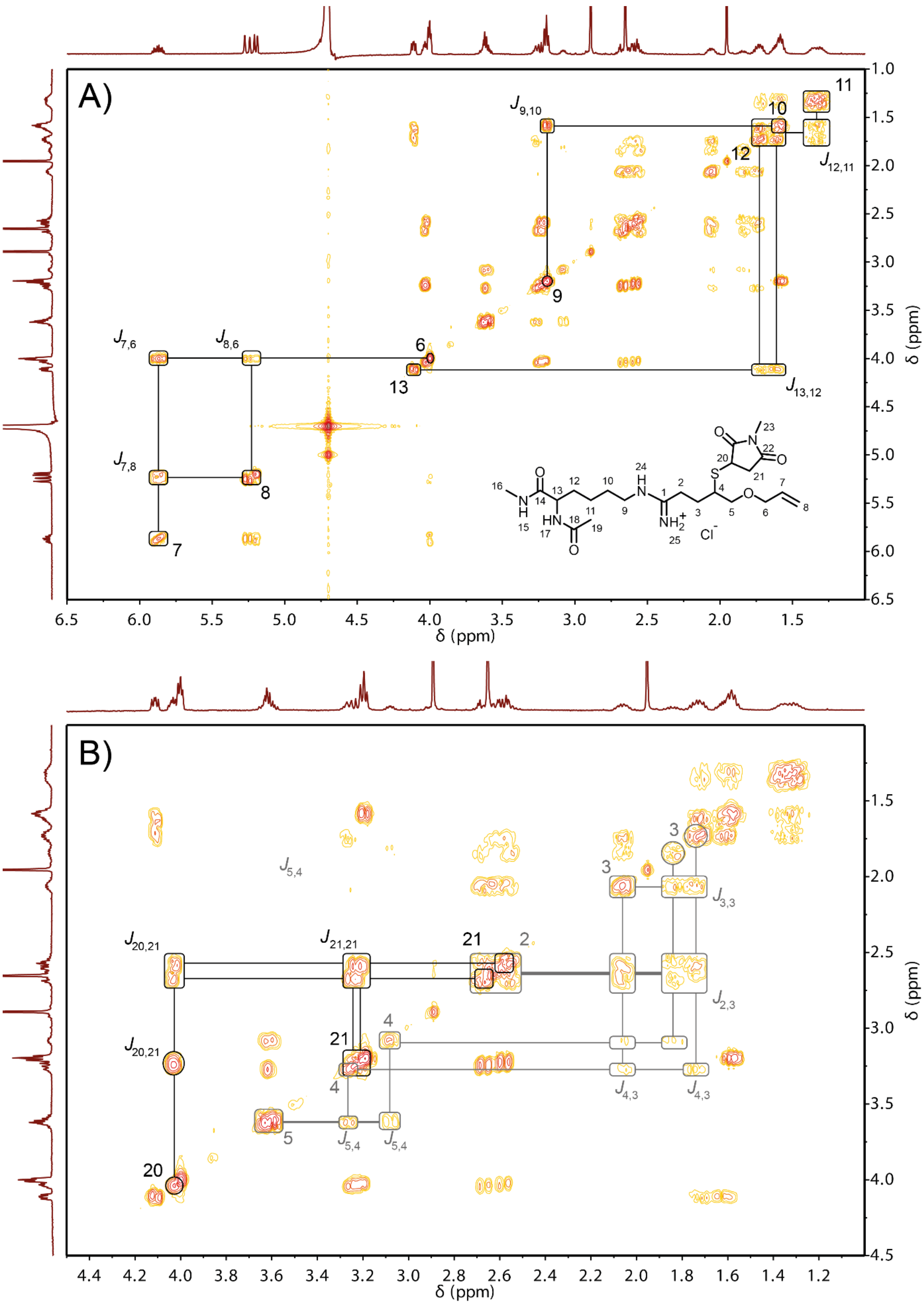

Figure S33. A) Full and B) magnified $^{1}H,^{1}H$-COSY spectrum of **4*** with detailed assignment. Recorded in D_2O.

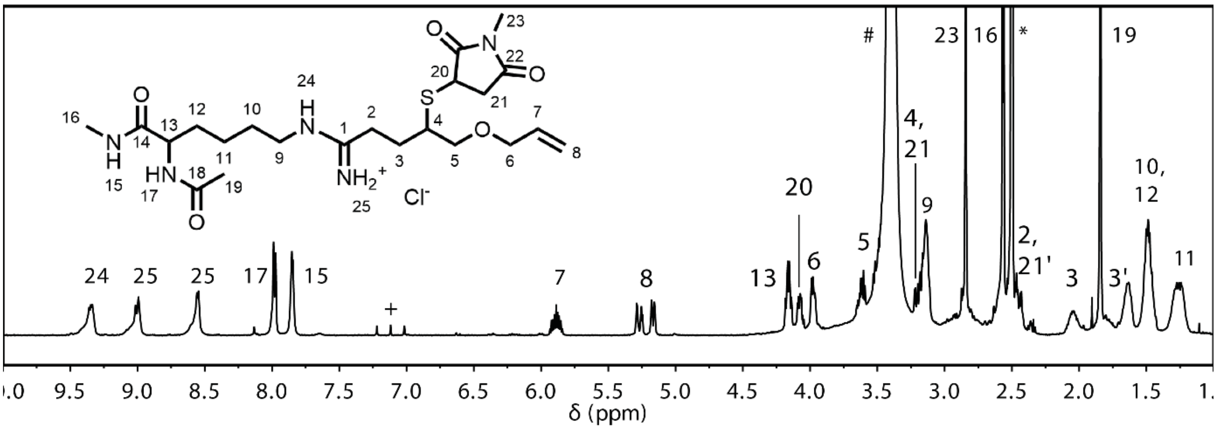

Figure S34. ^{1}H NMR spectrum of adduct **4***. Recorded in DMSO-d_6. Residual peaks: D_2O (#), NH_4Cl (+).

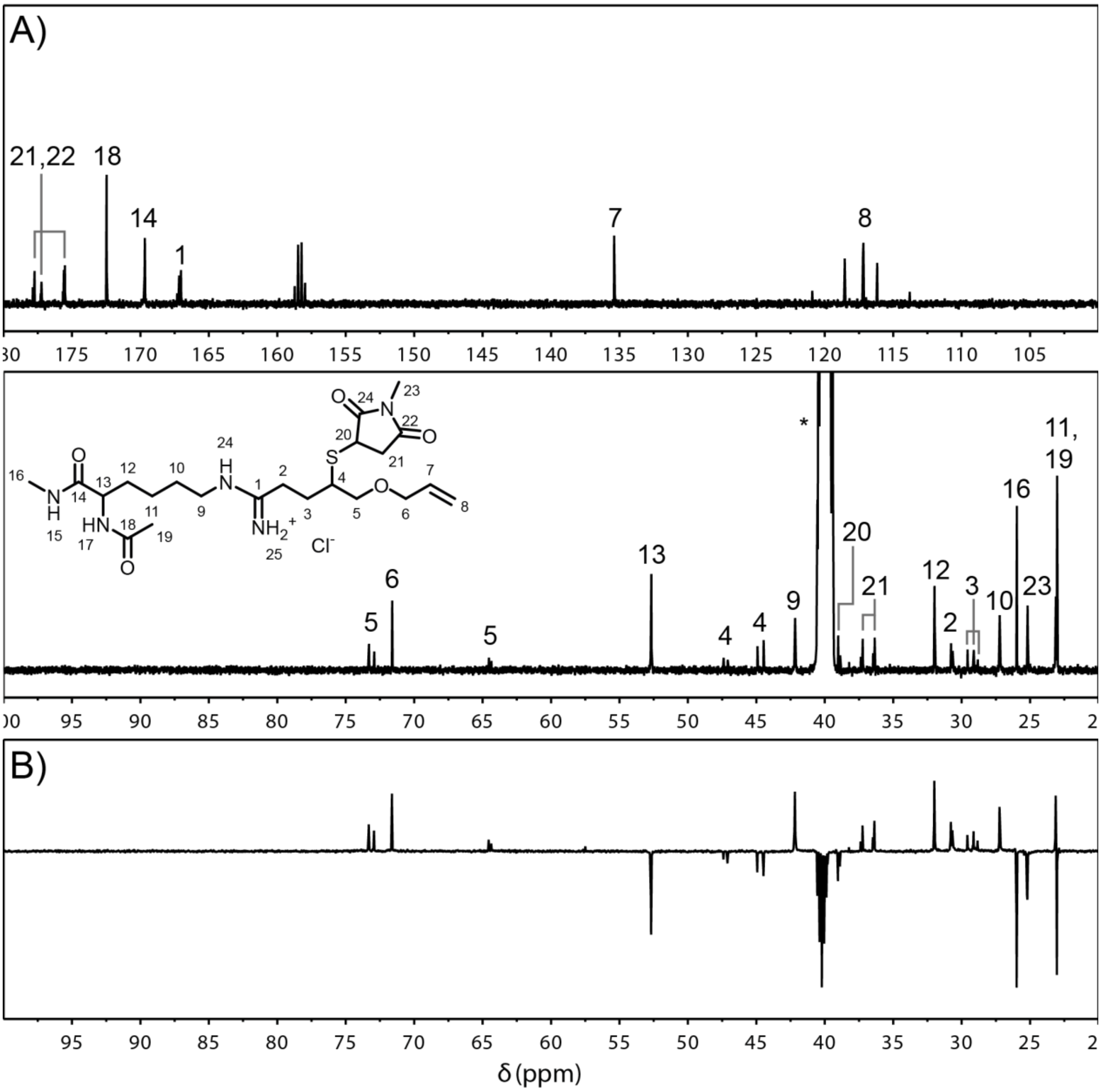

Figure S35. A) ^{13}C and B) DEPT135 NMR spectrum of adduct **4***. Recorded in DMSO-d_6 (*).

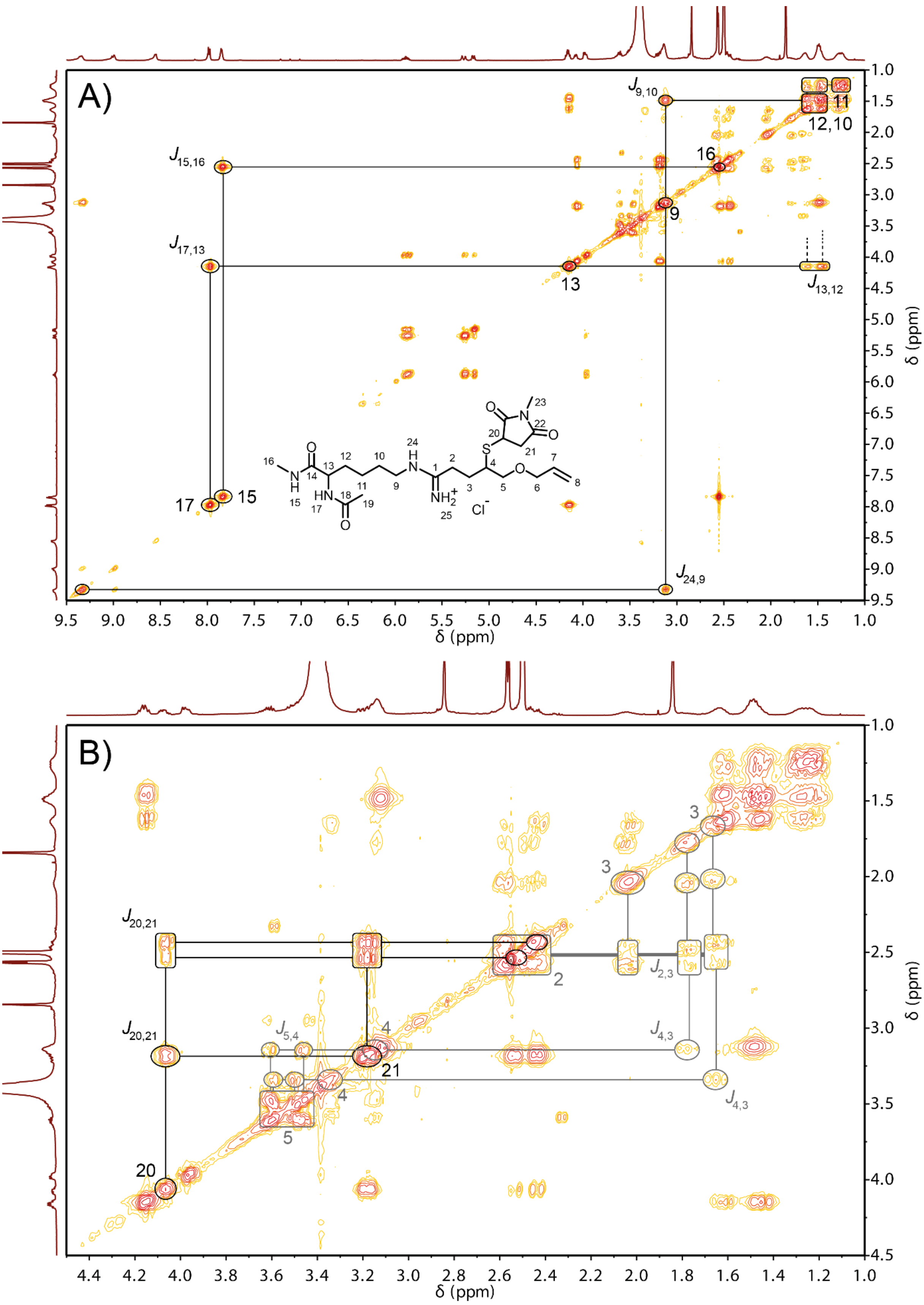

Figure S36. A) Full and B) magnified $^{1}H,^{1}H$-COSY spectrum of **4*** with detailed assignment. Recorded in DMSO-d_6.

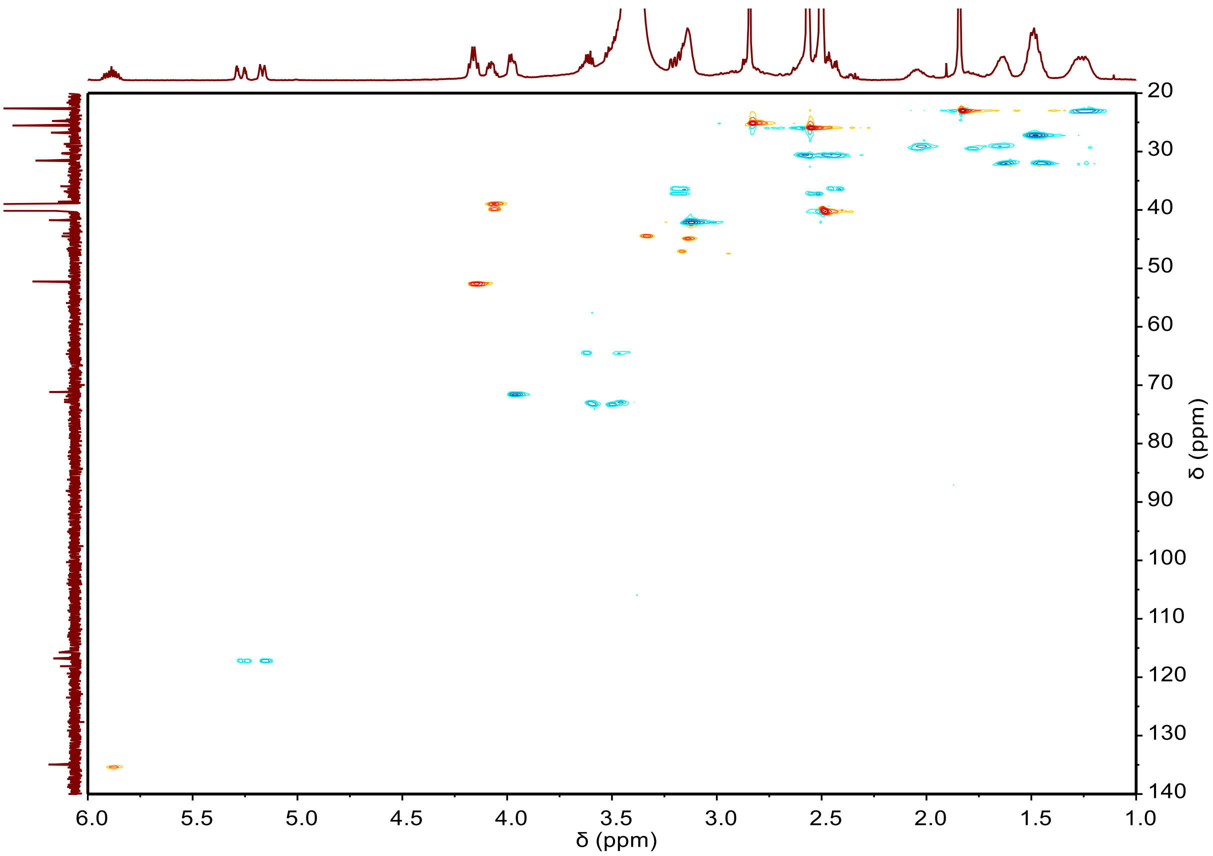

Figure S37. $^{1}H,^{13}C$-HSQC NMR spectrum of **4***. Recorded in DMSO-d_6.

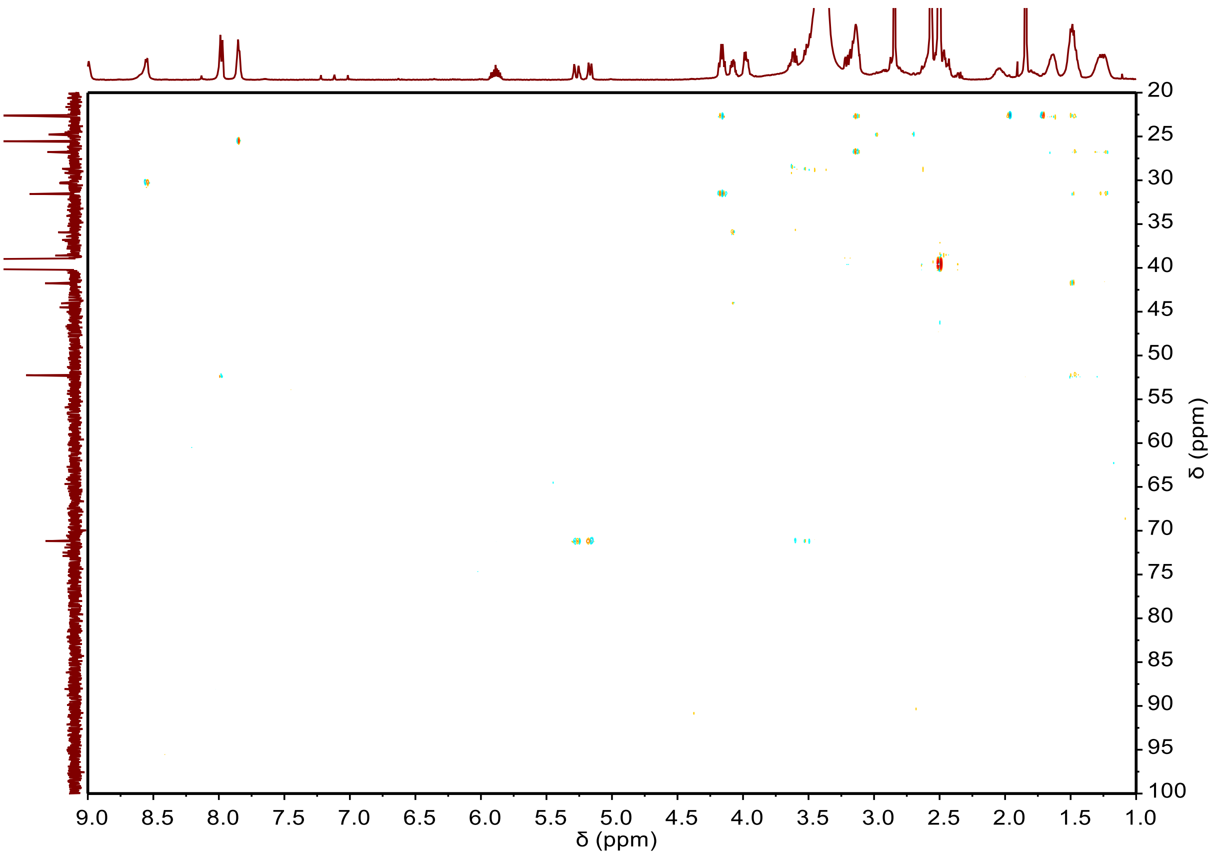

Figure S38. $^{1}H,^{13}C$-HMBC NMR spectrum of **4***, magnification 20-100 ppm. Recorded in DMSO-d_6.

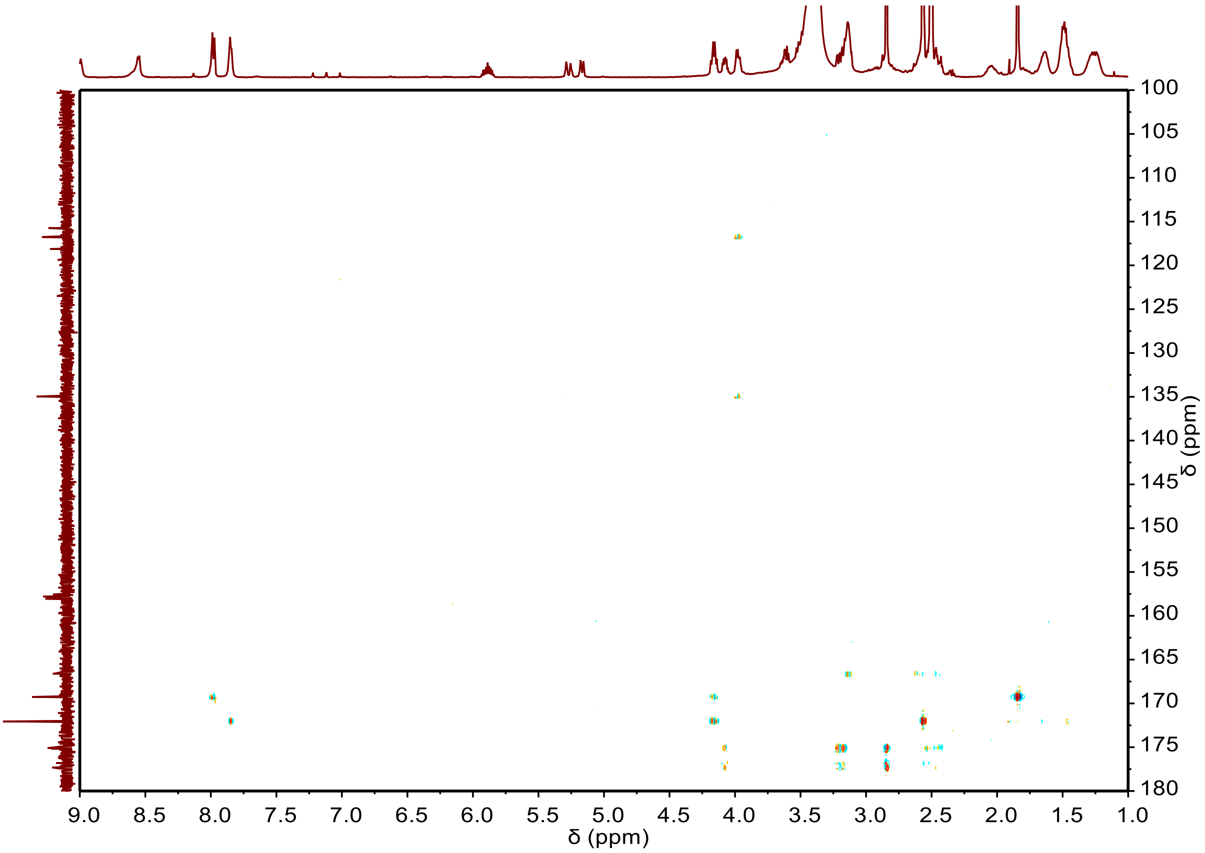

Figure S39. ^{1}H,^{13}C-HMBC NMR spectrum of **4***, magnification 100-180 ppm. Recorded in DMSO-d_6.

1.3.11 (E)-2-((5-((allyloxy)methyl)dihydrothiophen-2(3H)-ylidene)amino)ethan-1-ol (**5**)

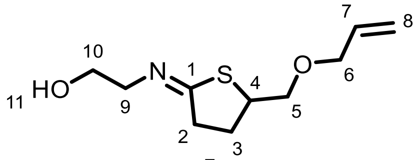

5

Ethanolamine (59.6 µL, 0.988 mmol) was added to a stirred solution of **3b** (205 mg, 0.988 mmol) in PBS (4.94 mL, [**3b**] = 0.2 M) at room temperature. The reaction mixture was stirred at room temperature for 4 h. Next, the solvent was evaporated under reduced pressure to give the crude product. Purification by flash column chromatography on silica gel using CH_2Cl_2 / 5% MeOH as eluent gave product **5** (105 mg, 49%) as a colorless oil. ^{1}H NMR (400 MHz, Deuterium Oxide) δ 6.00 – 5.86 (m, 1H, H7), 5.34 (d, J = 17.2 Hz, 1H, H8), 5.27 (d, J = 10.4 Hz, 1H, H8), 4.12 – 3.99 (m, 3H, H4/6), 3.86 – 3.78 (m, 3H, H5/10), 3.62 (dd, J = 10.5, 8.1 Hz, 1H, H5), 3.38 – 3.32 (m, 2H, H9), 2.85 – 2.68 (m, 2H, H2), 2.31 – 2.20 (m, 1H, H3), 1.98 – 1.84 (m, 1H, H3). ^{13}C NMR (101 MHz, D_2O) δ 178.8 (C1), 133.5 (C7), 118.4 (C8), 72.5 (C5), 71.9 (C6), 61.0 (C10), 58.2 (C9), 49.8 (C4), 37.5 (C2), 28.6 (C3). IR (ATR) 3303, 3089, 2937, 2864, 1643 (C=N), 1425, 1354, 1067, 995, 927. HRMS (ESI) m/z for $C_{10}H_{17}NO_2S$ $(M + H)^+$ 216.1054.

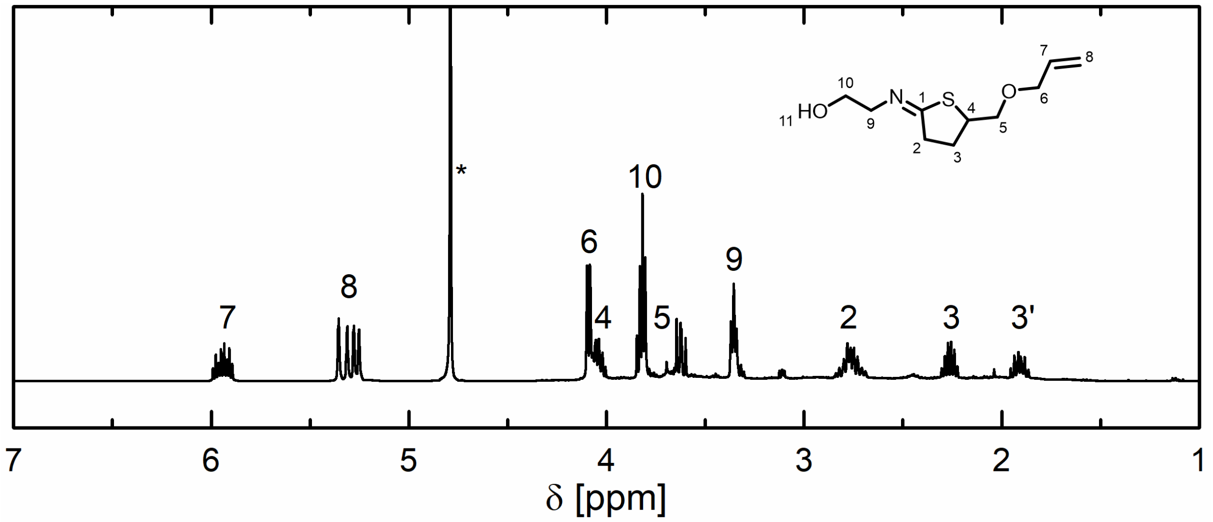

Figure S40. ^{1}H NMR spectrum of ITL adduct **5**. Recorded in D_2O (*).

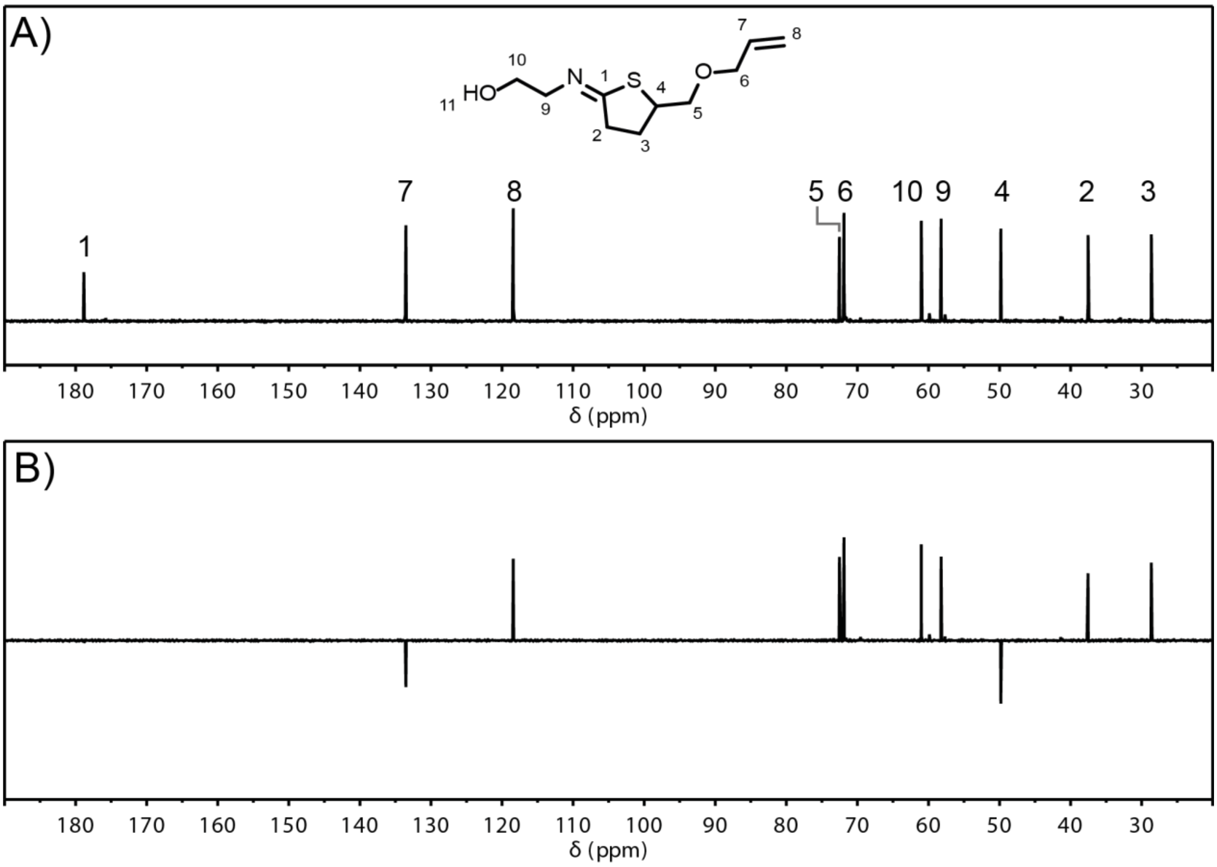

Figure S41. A) ^{13}C NMR and B) DEPT135 spectrum of adduct **5**. Recorded in D_2O.

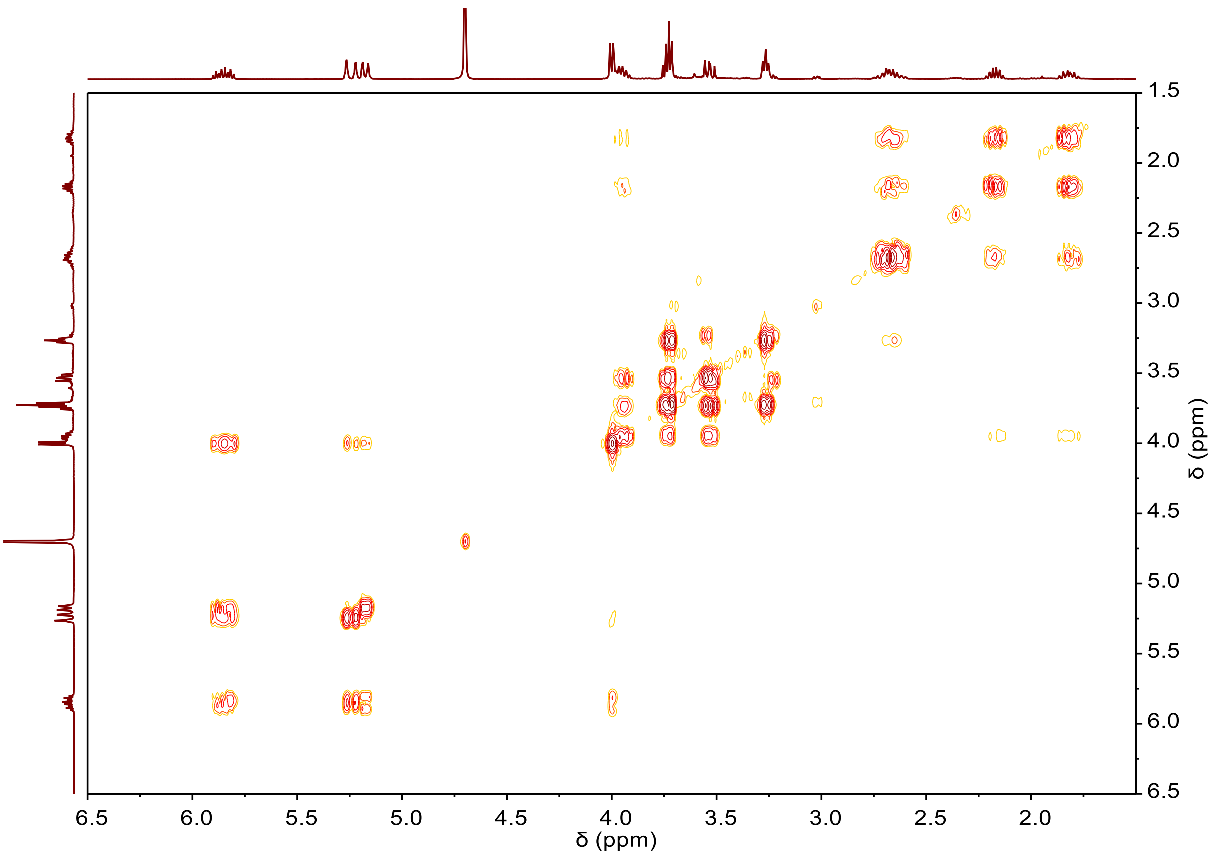

Figure S42. $^{1}H,^{1}H$-COSY NMR spectrum of **5**. Recorded in D_2O.

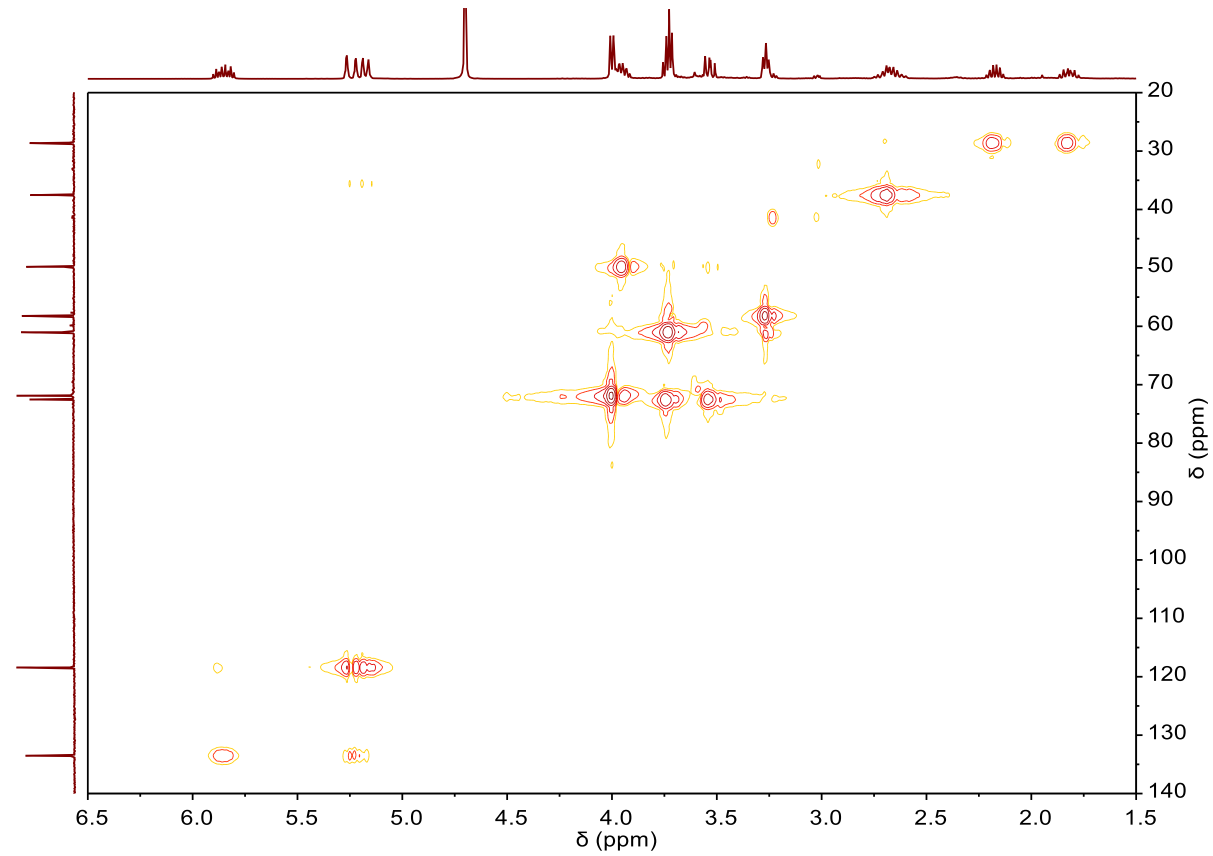

Figure S43. $^{1}H,^{13}C$-HSQC NMR spectrum of **5**. Recorded in D_2O.

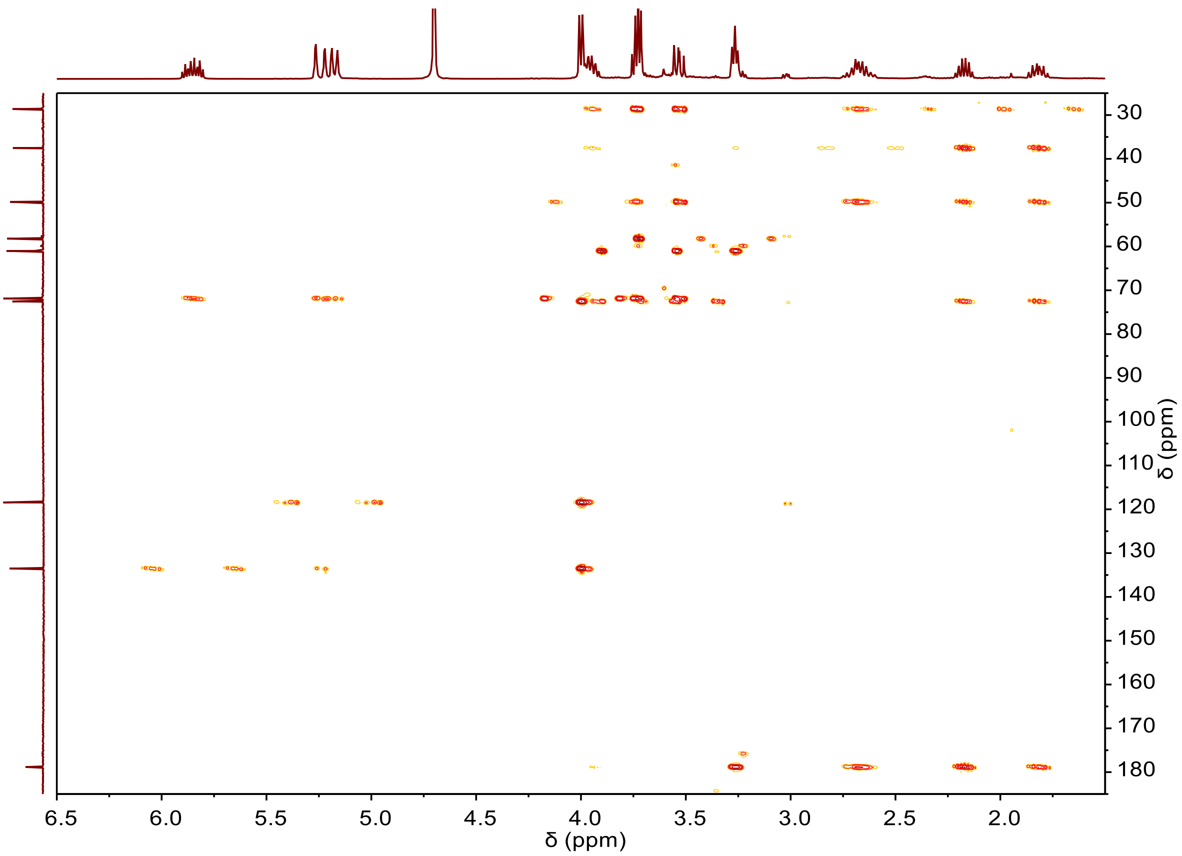

Figure S44. ^{1}H,^{13}C-HMBC NMR spectrum of **5**. Recorded in D_2O.

2 NMR kinetic measurements

2.1 Thiirane syntheses

By ^{1}H NMR spectroscopy the conversion of the glycidol derivative towards the thiirane was monitored. Aliquots of the reaction mixture were taken at chosen reaction times. As thiirane syntheses are unimolecular reactions ($A \rightarrow B$), the conversion could be easily calculated through integration of selected proton signals of the starting material A and product B according to the following equation: $x = \frac{I_B}{I_A + I_B}$

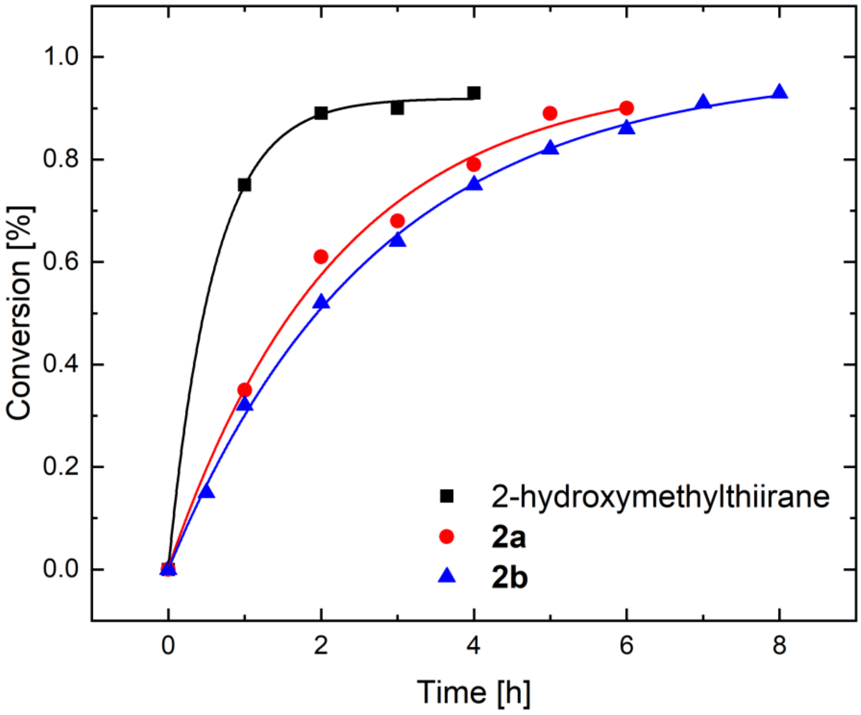

Figure S45. Kinetic data for the synthesis of functional thiiranes from glycidol derivatives. Depending on the glycidol functionality reaction rates vary significantly. Glycidol reacts much faster than corresponding glycidyl ethers, yet various side-products are produced.

2.2 Model reaction kinetics

By ^{1}H NMR spectroscopy the single modification of **3b** with K' and MEA was monitored. K' (11.6 mg, 5.78E-2 mmol) **or** MEA (3.5 µL, 5.78E-2 mmol) was added to a solution of **3b** (12.0 mg, 5.78E-2 mmol) in deuterated PBS (0.578 mL, [**3b**] = 0.1 M) in an NMR tube at room temperature. The sample was shimmed to D_2O at the start of the multizg measurement row. Once the first measurement was started, measurements were conducted in a time interval of 20 minutes for a total time of 24 hours.

2.3 Hydrolysis and pH stability of ITLs

As iminium thiolactones are the more reactive counterparts of thiolactones, they are also more prone to hydrolysis. This hydrolysis may happen according to the following mechanism and

was verified by comparing the side product precipitate by commercially available ammonium chloride.

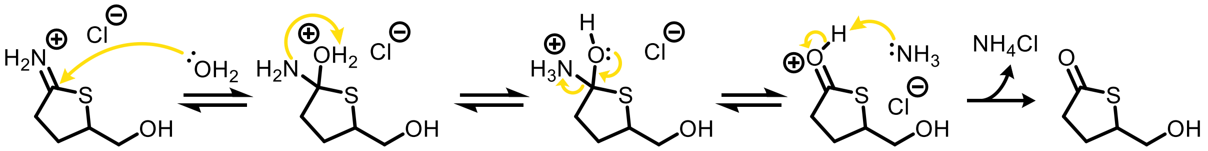

Figure S46. Mechanism for the hydrolysis of hydroxy ITL **3a** resulting in ammonium chloride as by-product.

By ^{1}H NMR spectroscopy the hydrolysis of ITL ring towards the thiolactone was monitored. The samples (c 0.1 M) were shimmed to the appropriate solvent (10 mM PBS/D_2O, pH = 7.4) at the start of the multizg measurement row. Once the first measurement was started, measurements were conducted in a time interval of 20 minutes for a total time of 24 hours. According to the used standard procedure. compound **3b** is obtained as chloride salt. However, when the cyclization step is carried out with hydrobromic acid instead of hydrochloric acid, the bromide salt can easily be obtained. We synthesized a small sample of the hydrobromide salt to assess impact of the counterion towards the hydrolysis rate.

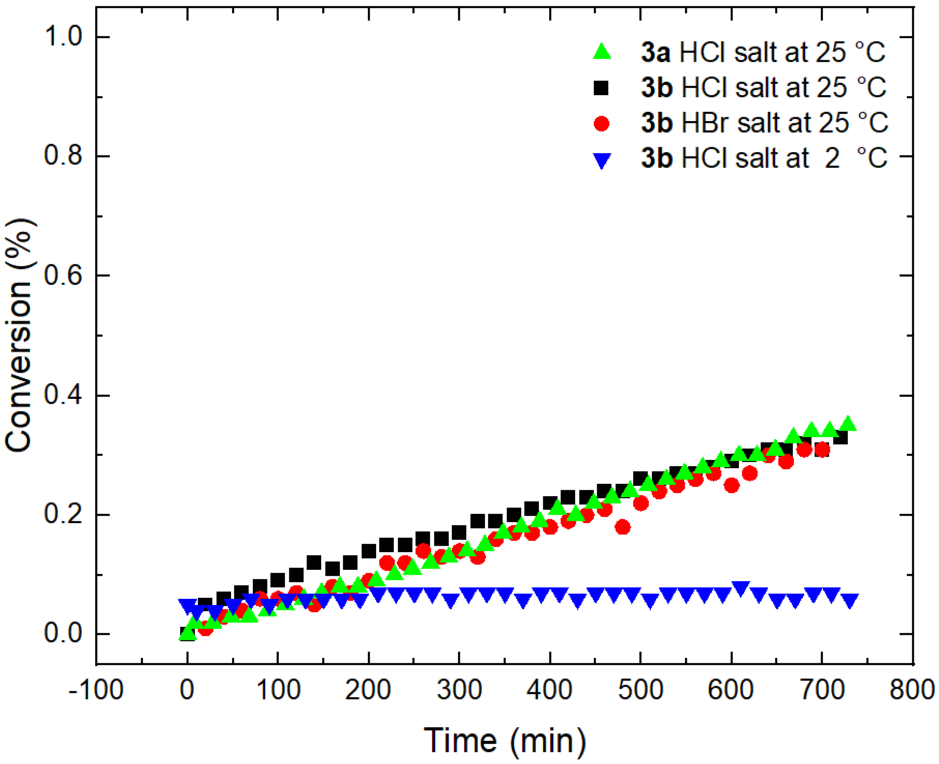

Figure S47. Hydrolysis of the ITL heterocycle to form the corresponding thiolactone under cleavage of ammonium chloride.

Looking at the kinetic data, all ITLs hydrolysed with the same rate, reaching approximately 33% conversion after 12 h. The hydrolysis could be effectively halted by reducing the storage temperature to 2 °C. In another experiment, a small amount of **3b** was left to hydrolyse completely, after which an 1H NMR spectrum was measured (Figure S48).

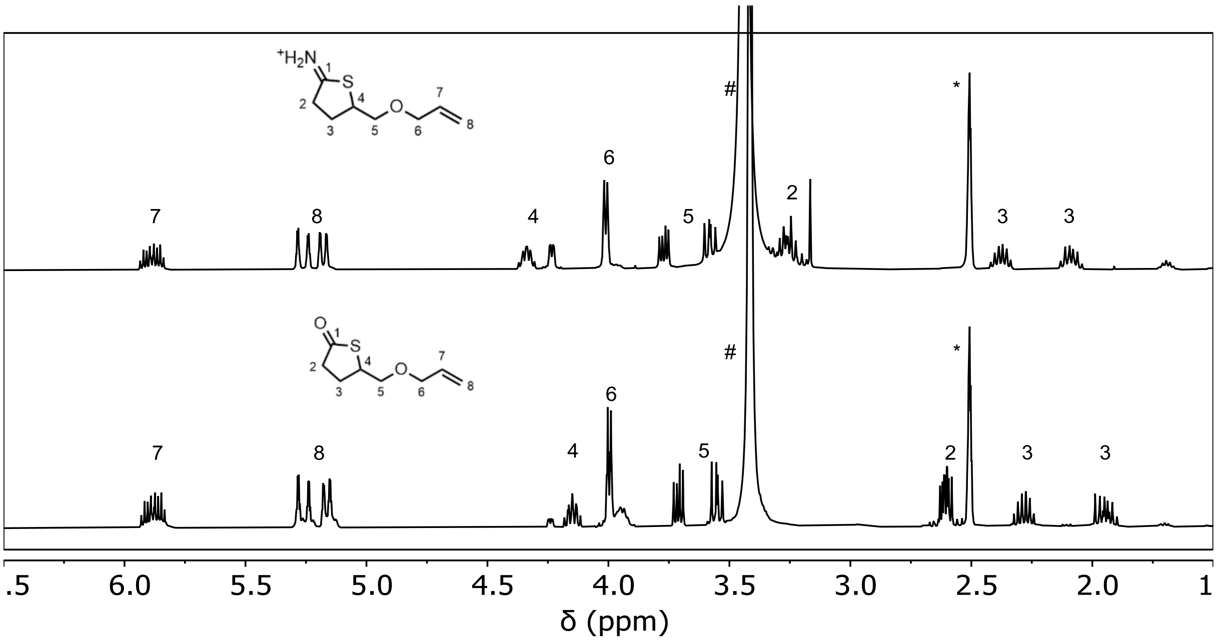

Figure S48. ^{1}H NMR spectra of iminium thiolactone **3b** (top) and the thiolactone product of its (bottom). Residual solvent peaks: DMSO-d_6 (*), D_2O (#).

When **3a** is reacted with strong and nucleophilic bases, such as hydroxide, ring-opening occurs with the formation of the corresponding amide after tautomerisation (Figure S49).

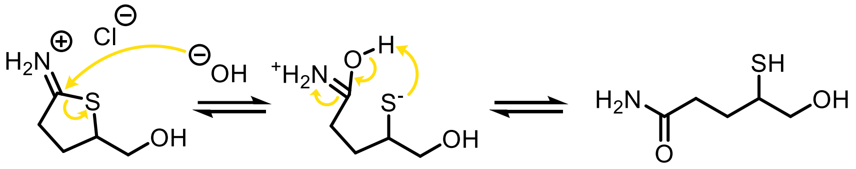

Figure S49. Mechanism for the reaction of hydroxy ITL **3a** with hydroxide resulting in a ring-opened amide.

The pH stability of ITL **3a** was investigated *via* ^{1}H NMR spectroscopy. First, a known amount of **3a** was dissolved in D_2O. The total volume of this sample was fixed at 4.5 mL. One aliquot of 0.5 mL of this solution was filled into an NMR tube, while another aliquot (2 mL) was filled into a 15 mL centrifuge tube. Small volumes of either a 4% NaOD or 4% DCl solution were added to the NMR tube and the respective 4-fold volume of base or acid was added simultaneously to the centrifuge tube. After each addition, a ^{1}H NMR spectrum was measured of the 0.5 mL aliquot, while the pH was measured by immersing the pH electrode into the 2 mL aliquot.

When small amounts of NaOD were added to **3a**, the compound experienced a buffering capacity. When the sample was measured immediately after the addition of NaOD, all signals were shifted upfield. With progressing time, however, the signals shifted back downfield (see Figure S50, 20 min after addition of 2 μL 4% NaOD). This shift occurred alongside the emergence of a new set of signals. When more base was added to this mixture, the residual amount of **3a** was transformed to this new species (Figure S50, spectrum with pH = 10.40). This reaction seemed to be irreversible as adding excess of 4% DCl solution did not affect the

signal shifts of this newly formed compound. The full spectrum of the resulting amide is shown in Figure S51).

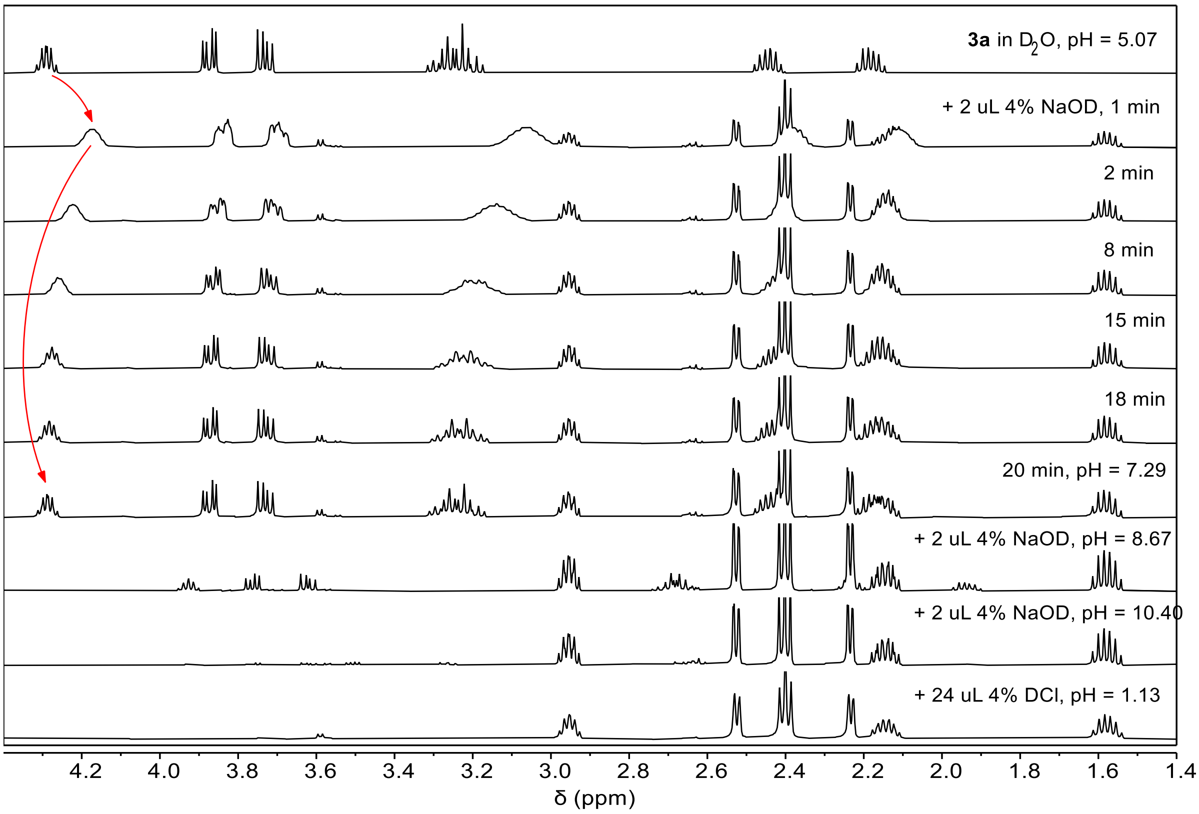

Figure S50. ^{1}H NMR spectra of **3a** upon addition of small amounts of a 4% NaOD solution, recorded in D_2O.

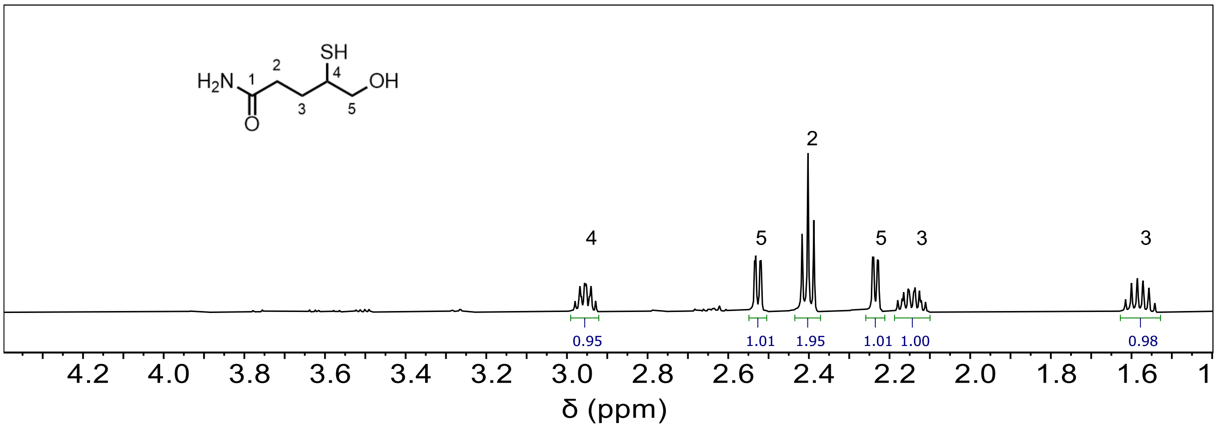

Figure S51. ^{1}H NMR spectrum of the amide that is formed through reaction of **3a** with NaOD, recorded in D_2O. The amide, hydroxy, and thiol signals were not detected due to proton-deuterium exchange with D_2O.

Analogous behaviour was observed when **3b** was reacted with small amounts of a 4% solution of NaOD. The reaction proceeded, however, slower and in the final NMR spectrum a 50:50 product mixture of **3b** and the ring-opened amide was observed (Figure S52).

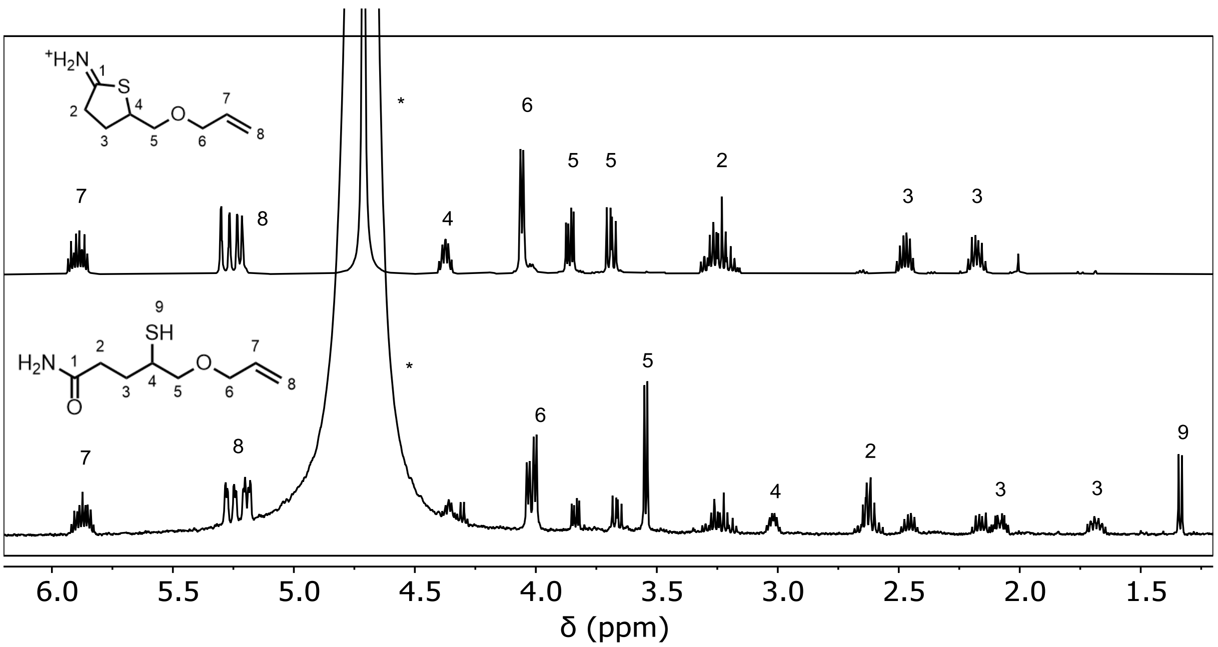

Figure S52. ^{1}H NMR spectra of **3b** (top) and a mixture of **3b** and ring-opened amide (bottom) that is formed through reaction of **3b** with NaOD, recorded in D_2O (*).

3 Crystallographic data of 3a

Suitable crystals for single crystal X-ray diffraction were obtained by recrystallization in MeOH using the vapor diffusion method (diethyl ether) to obtain colourless blocks.

Intensity data were collected on a Bruker D8-QUEST diffractometer with a Photon-100 detector using Cu-Kα radiation (λ = 1.54178 Å) from an Incoatec IμS microsource with multilayer optics. Crystals were mounted on a MiTeGen crystal mount using inert polyfluoroether oil and the analysis was carried out at 180 K under an Oxford Cryosystems open-flow N_2 Cryostream. The control and processing software was Bruker *APEX2*.[4] The diffraction images were integrated using *SAINT* in *APEX4*, and a multi-scan correction was applied using *SADABS*.[5] The final unit-cell parameters were refined against all reflections. Structures were solved using *SHELXT*[6] and refined using *SHELXL*.[7] The H atoms of the NH_2 group were refined freely with isotropic displacement parameters. Other H atoms were placed in geometric positions and allowed to ride during subsequent refinement.

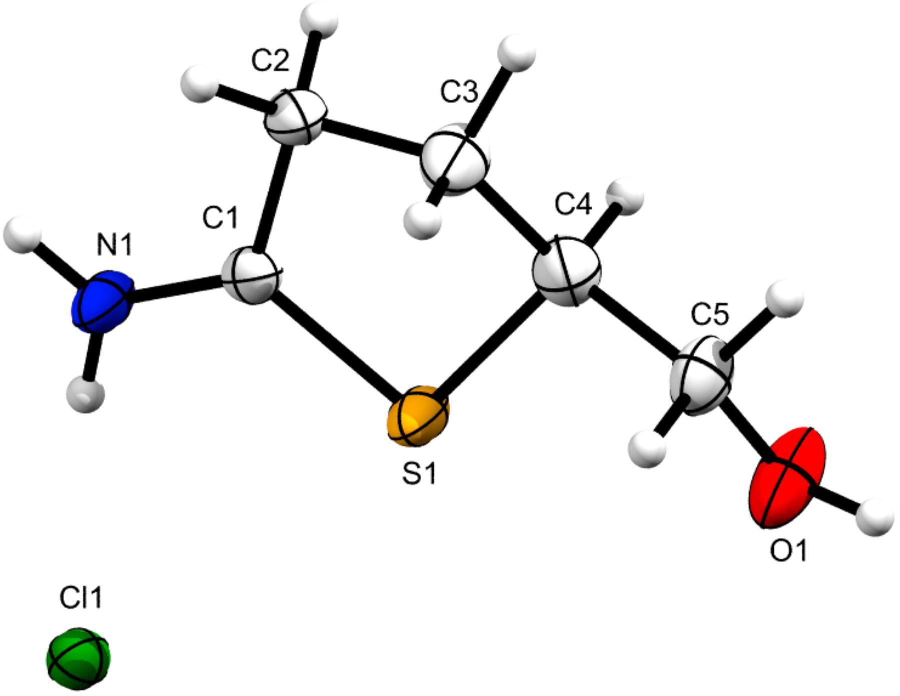

Figure S53. Displacement ellipsoid plot of **3a**.

Table S4. Crystal data and refinement results of compound **3a**.

Chemical formula	$C_5H_{10}ClNOS$
M_r	167.65
Crystal system, space group	orthorhombic, P c a 2_1
T (K)	180(2)
a, *b*, *c* (Å)	15.0306(6), 5.7830(2), 9.0459(3)
α, β, γ (°)	90, 90, 90
V (Å^3)	786.29(5)
Z	4
μ (mm^{-1})	6.177
Flack parameter	0.01(3)
Crystal shape, crystal size (mm)	block, 0.280 x 0.180 x 0.120
Index ranges	$-17 \leq h \leq 14$, $-6 \leq k \leq 6$, $-10 \leq l \leq 10$
Reflections collected	3353
Independent reflections	1239 [R_{int} = 0.0281]
T_{min}, T_{max}	0.5011, 0.7528
Data/ restraint/ parameters	1239 / 1 / 91
Goodness-of-fit on F^2	1.140
Final R indices [I>2σ(I)]	R_1 = 0.0342, wR_2 = 0.0885
R indices (all data)	R_1 = 0.0351, wR_2 = 0.0896
Largest diff. peak and hole (e Å^{-3})	0.461 and -0.249 e Å^{-3}

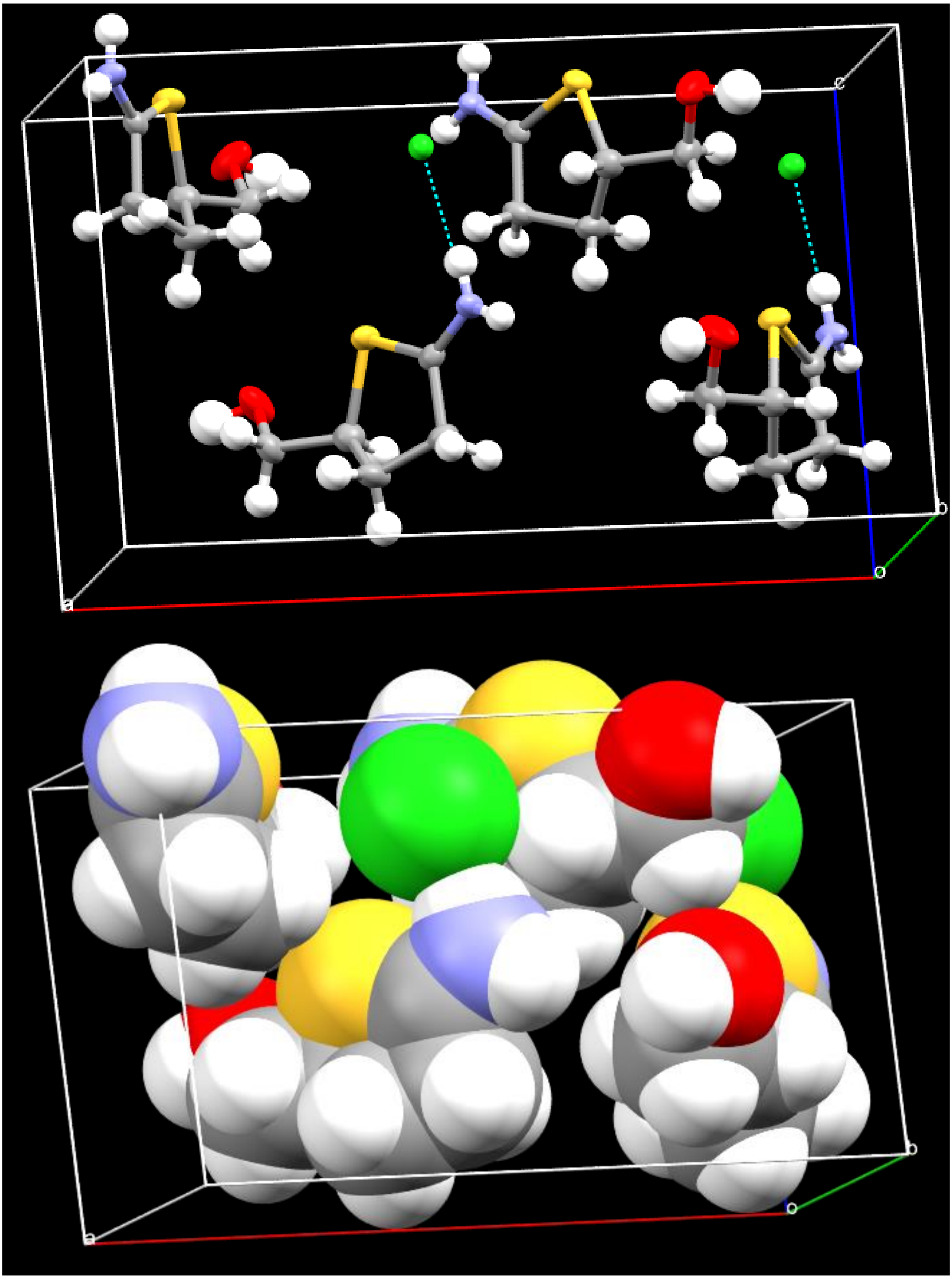

Figure S54. Unit cell of compound **3a** in ball and sticks (top) and space-filling (bottom) view.

4 Calculations

Density functional theory calculations were carried out with the Gaussian ‘09 software package.[8] Becke’s three parameter hybrid exchange functional[9] combined with Lee–Yang–Parr non-local correlation functional[10] abbreviated as B3LYP is used and the 6-311G++(2d,p) basis set was employed. The conductor-like polarizable continuum model (CPCM) was used as solvation method to simulate an aqueous environment. Graphical outputs of the computational results were generated with the GaussView software program (ver. 6.0.16) developed by Semichem, Inc.[11]

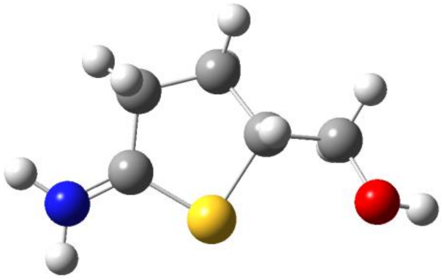

Figure S55. DFT-computed ground-state geometry of γ-hydroxy iminiumthiolactone **3a**.

Imaginary frequencies: none

E = –724.701906 Hartree

ZPE correction: 0.153130 Hartree/Particle

Sum of electronic and thermal Free Energies= –724.582250 Hartree

Center	Atomic	Atomic	Coordinates (Angstrom)		
Number	Number	Type	X	Y	Z
1	6	C	-0.854	0.492	-0.339
2	6	C	0.035	1.634	0.152
3	6	C	1.482	1.276	-0.19
4	1	H	-1.002	0.532	-1.418
5	1	H	-0.072	1.745	1.233
6	1	H	-0.261	2.573	-0.316
7	1	H	1.7	1.478	-1.246
8	1	H	2.216	1.821	0.404
9	6	C	1.626	-0.202	0.004
10	7	N	2.783	-0.776	0.164
11	1	H	2.884	-1.778	0.259
12	1	H	3.632	-0.225	0.193
13	16	S	0.147	-1.061	-0.035
14	6	C	-2.193	0.384	0.356
15	1	H	-2.751	1.309	0.168
16	1	H	-2.055	0.275	1.436
17	8	O	-2.876	-0.744	-0.188
18	7	H	-3.695	-0.879	0.302

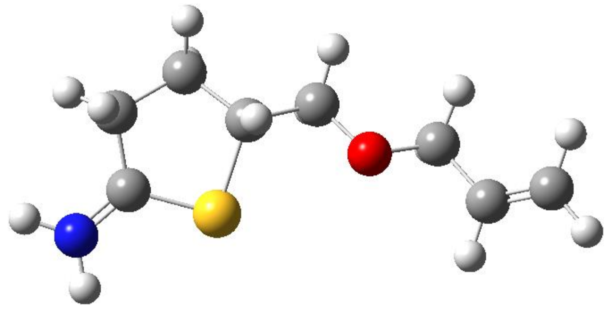

Figure S56. DFT-computed ground-state geometry of γ-allyloxymethyl iminiumthiolactone **3b.**

Imaginary frequencies: none

E = –841.431452 Hartree

ZPE correction: 0.214218 Hartree/Particle

Sum of electronic and thermal Free Energies= –841.257025 Hartree

Center Number	Atomic Number	Atomic Type	Coordinates (Angstrom) X	Y	Z
1	6	C	0.568	0.773	-0.375
2	6	C	1.742	1.663	0.034
3	6	C	3.031	0.918	-0.312
4	1	H	0.402	0.795	-1.452
5	1	H	1.704	1.854	1.108
6	1	H	1.688	2.621	-0.483
7	1	H	3.256	0.993	-1.383
8	1	H	3.903	1.284	0.231
9	6	C	2.789	-0.531	-0.024
10	7	N	3.76	-1.377	0.166
11	1	H	3.599	-2.363	0.328
12	1	H	4.723	-1.066	0.152
13	16	S	1.136	-0.971	0.003
14	6	C	-0.728	1.063	0.35
15	1	H	-1.029	2.094	0.121
16	1	H	-0.592	0.981	1.435
17	6	C	-2.976	0.325	0.539
18	1	H	-3.37	1.321	0.306
19	1	H	-2.837	0.259	1.626
20	8	O	-1.706	0.142	-0.101
21	6	C	-3.912	-0.741	0.07
22	1	H	-3.579	-1.764	0.224
23	6	C	-5.097	-0.5	-0.479
24	1	H	-5.45	0.513	-0.644
25	1	H	-5.758	-1.306	-0.775

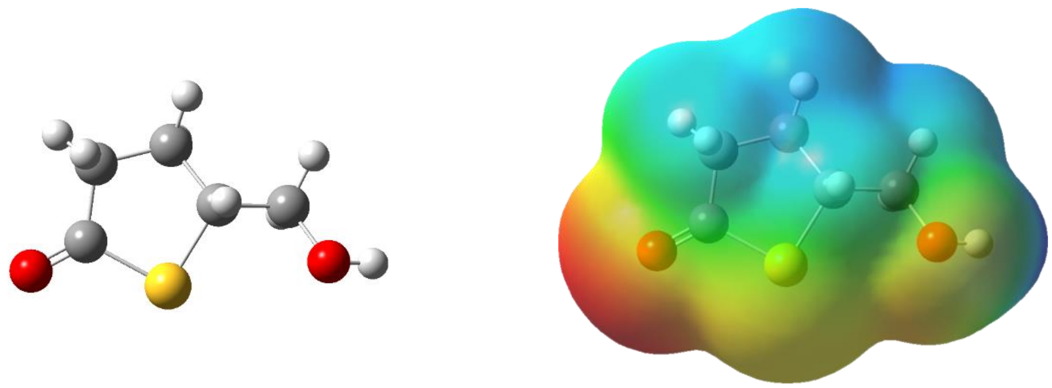

Figure S57. DFT-computed ground-state geometry (left) and electron density mapping (right) of γ-hydroxymethyl thiolactone.

Imaginary frequencies: none

E = –744.144103 Hartree

ZPE correction: 0.127256 Hartree/Particle

Sum of electronic and thermal Free Energies= –744.050176 Hartree

Center Number	Atomic Number	Atomic Type	Coordinates (Angstrom) X	Y	Z
1	6	C	-0.782	0.445	-0.335
2	6	C	0.123	1.587	0.148
3	6	C	1.564	1.233	-0.202
4	1	H	-0.936	0.503	-1.414
5	1	H	0.023	1.692	1.232
6	1	H	-0.186	2.529	-0.308
7	1	H	1.771	1.42	-1.262
8	1	H	2.305	1.784	0.378
9	6	C	1.76	-0.255	0.021
10	16	S	0.181	-1.109	-0.019
11	6	C	-2.127	0.416	0.357
12	1	H	-2.62	1.382	0.196
13	1	H	-1.994	0.271	1.434
14	8	O	-2.907	-0.643	-0.203
15	1	H	-3.753	-0.678	0.258
16	8	O	2.814	-0.822	0.186

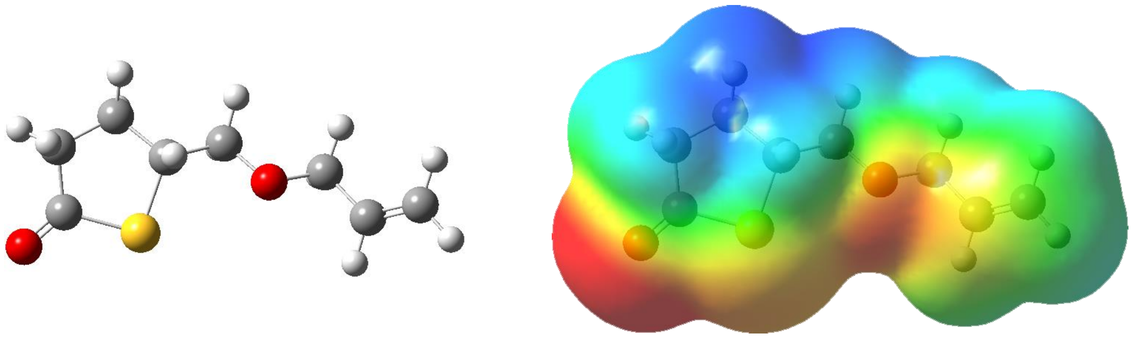

Figure S58. DFT-computed ground-state geometry (left) and electron density mapping (right) of γ-allyloxymethyl thiolactone.

Imaginary frequencies: none

E = –860.873671 Hartree

ZPE correction: 0.188217 Hartree/Particle

Sum of electronic and thermal Free Energies= –860.725106 Hartree

Center Number	Atomic Number	Atomic Type	Coordinates (Angstrom) X	Y	Z
1	6	C	0.621	0.672	-0.366
2	6	C	1.776	1.601	0.032
3	6	C	3.087	0.908	-0.322
4	1	H	0.452	0.705	-1.443
5	1	H	1.737	1.786	1.109
6	1	H	1.676	2.562	-0.475
7	1	H	3.297	0.983	-1.395
8	1	H	3.952	1.306	0.21
9	6	C	2.946	-0.571	-0.011
10	16	S	1.214	-1.04	0.027
11	1	C	-0.674	0.988	0.351
12	1	H	-0.949	2.031	0.14
13	1	H	-0.547	0.885	1.436
14	6	C	-2.948	0.337	0.53
15	1	H	-3.308	1.349	0.306
16	1	H	-2.812	0.259	1.618
17	8	O	-1.689	0.11	-0.112
18	6	C	-3.925	-0.692	0.059
19	1	H	-3.624	-1.727	0.197
20	6	C	-5.107	-0.407	-0.475
21	1	H	-5.429	0.618	-0.626
22	1	H	-5.797	-1.187	-0.772
23	8	O	3.848	-1.354	0.171

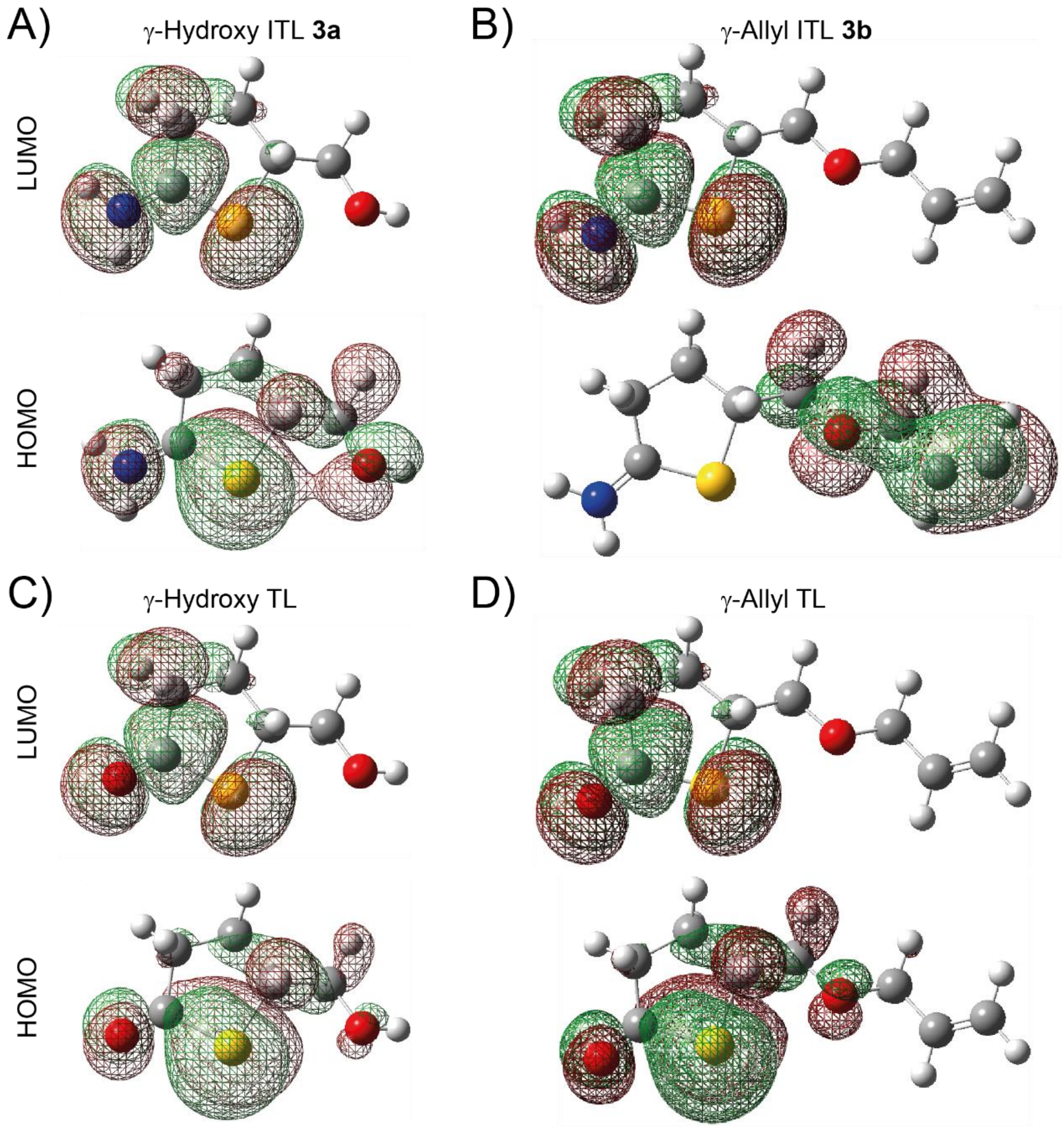

Figure S59. DFT-calculated visualization of the highest occupied molecular orbitals (HOMO) and the lowest unoccupied molecular orbitals (LUMO) of γ-functional ITLs **3a** and **3b**, as well as the respective TL surrogates.

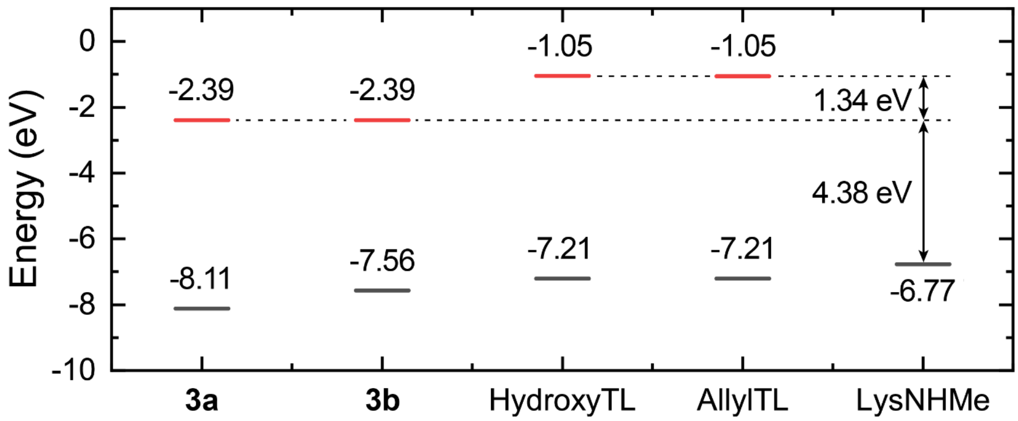

Figure S60. Molecular orbital energy diagram with HOMO-LUMO energy gaps.

5 HPLC kinetic measurements

Kinetic HPLC measurements were carried out on a semi-preparative Varian 940-LC system. A sample volume of 50 μL was injected and the HPLC trace was recorded every 30 minutes. A Varitide RPC column with a 5 micron pore size, a 110 Å particle size and with the dimensions 150 x 21.2 mm. An isocratic composition of 20% acetonitrile 80% water with 0.1% TFA was used as eluent at a flow rate of 1 mL/min over 12 min. A diode array detector (DAD) was used to monitor the consumption of **3b** (λ_{max} = 244 nm) as well as the starting material signals and emerging product signals. Stock solutions were prepared freshly in water, and reactants were diluted to a concentration of 1 mM in a chosen pH buffered medium (10 mM PBS, pH = 7.4 or 20 mM NaPi, pH = 8.0), right before the first injection, and with this the start of the kinetic experiment. An exemplary procedure is outlined below:

Kinetic measurement of **4**:

Stock solution **A** comprised **3b** (5.6 mg/mL in H_2O), and stock solution **B** contained **K'** (5.7 mg/mL in H_2O). Solution **A** (297 μL) was added to a vial filled with 10 mM PBS (7.393 mL, pH = 7.4). To start the reaction, solution **B** (311 μL) was added to the reaction mixture and the final solution (8 mL, [**3b**] = 1 mM, [**K'**] = 1.1 mM) was quickly filtered (0.2 μm Nylon filters) into a fresh HPLC vial prior periodic injection into the HPLC system.

Kinetic measurement of **4***:

The following stock solutions were prepared: **A**) **3b** (8.9 mg/mL in H_2O), **B**) **K'** (7.2 mg/mL in H_2O) and **C**) NMM (7.7 mg/mL in H_2O). Solution **A** (187 μL) and solution **C** (127 μL) were added to a vial filled with 10 mM PBS (7.440 mL, pH = 7.4). To start the reaction, solution **B** (246 μL) was added to the reaction mixture and the final solution (8 mL, [**3b**] = 1 mM, [**K'**] = 1.1 mM, [NMM] = 1.1 mM) was quickly filtered (0.2 μm Nylon filters) into a fresh HPLC vial prior periodic injection into the HPLC system. Reactions with different peptides and/or buffer solutions were carried out analogously (Table S5).

Table S5. Experimental data of the kinetic measurements for the single and double modification of **3a** using various substrates and buffer solutions.

Product	Solution **A**		Solution **B**			Solution **C**		Solvent	
	[mg/mL]	[μL]	Substrate	[mg/mL]	[μL]	[mg/mL]	[μL]	Buffer	[mL]
**4**	5.6	297	**K'**	5.7	311	-	-	PBS	7.393
**4**	5.6	297	**K'**	5.7	311	-	-	NaPi	7.393
**6**	10.0	166	KFRGDS	18.8	491	-	-	PBS	7.342
**6**	10.0	166	KFRGDS	18.8	491	-	-	NaPi	7.342
**7**	6.3	264	GRGDS	12.8	493	-	-	PBS	7.242
**7**	6.3	264	GRGDS	12.8	493	-	-	NaPi	7.242
**8**	6.7	248	KLVFF	7.9	981	-	-	PBS	6.771
**8**	6.7	248	KLVFF	7.9	981	-	-	NaPi	6.771
**4***	8.9	187	**K'**	7.2	246	7.7	127	PBS	7.440
**4***	8.9	187	**K'**	7.2	246	7.7	127	NaPi	7.440
**6***	8.0	208	KFRGDS	18.8	491	7.4	132	PBS	7.169
**6***	8.0	208	KFRGDS	18.8	491	7.4	132	NaPi	7.169
**7***	8.0	208	GRGDS	12.8	493	8.4	116	PBS	7.183
**7***	8.0	208	GRGDS	12.8	493	8.4	116	NaPi	7.183
**8***	5.6	297	KLVFF	7.9	981	8.0	122	PBS	6.600
**8***	5.6	297	KLVFF	7.9	981	8.0	122	NaPi	6.600

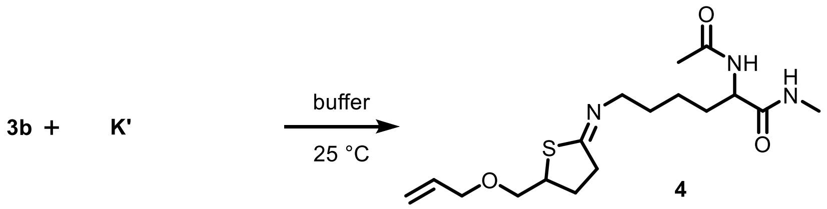

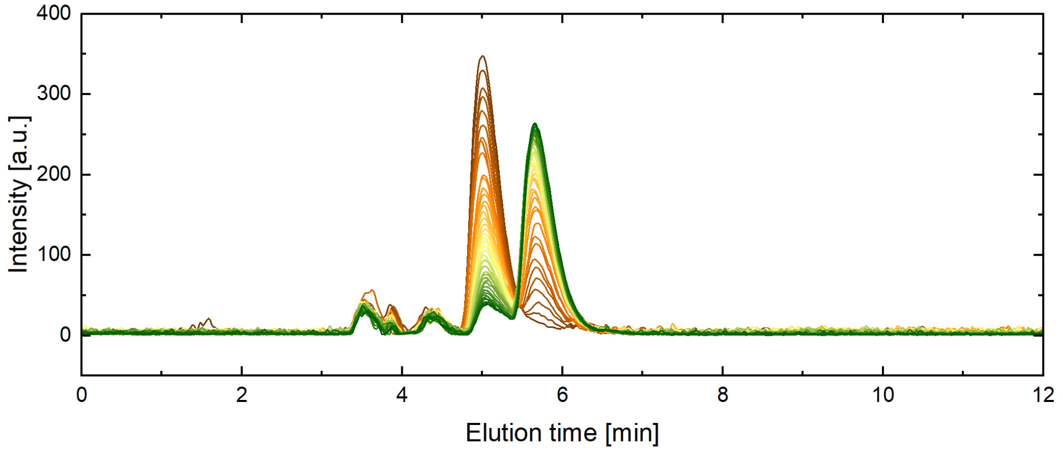

Figure S61. Combined maximum absorption HPLC trace showing the reaction of **3b** (5.63 min) and **K’** (4.38 min) in NaPi (20 mM, pH = 8.0) to give product **4** (5.00 min).

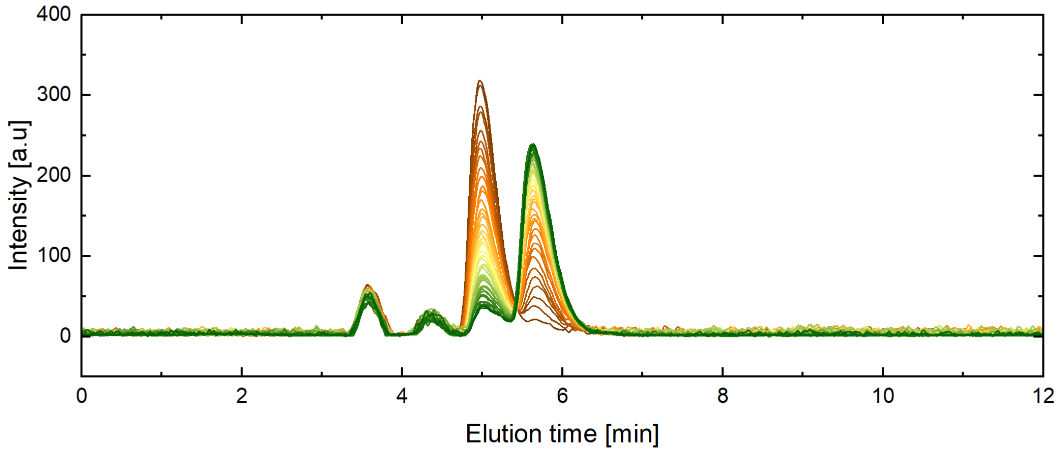

Figure S62. Combined maximum absorption HPLC trace showing the reaction of **3b** (5.63 min) and **K’** (4.38 min) in PBS (10 mM, pH = 7.4) to give product **4** (5.00 min).

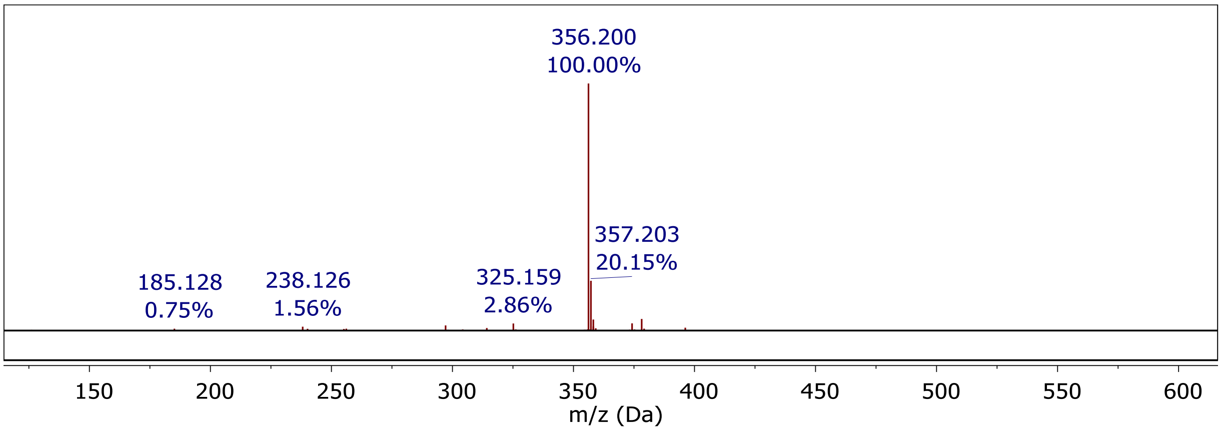

Figure S63. Mass spectrum of **4** showing the singly charged species (**4**+H)$^+$ = 356.200 Da.

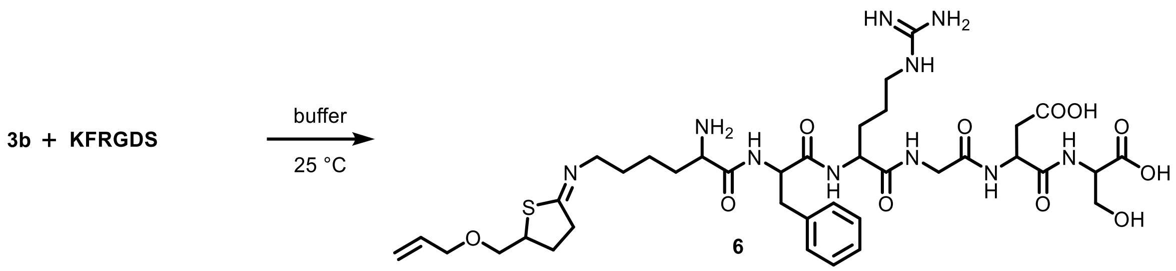

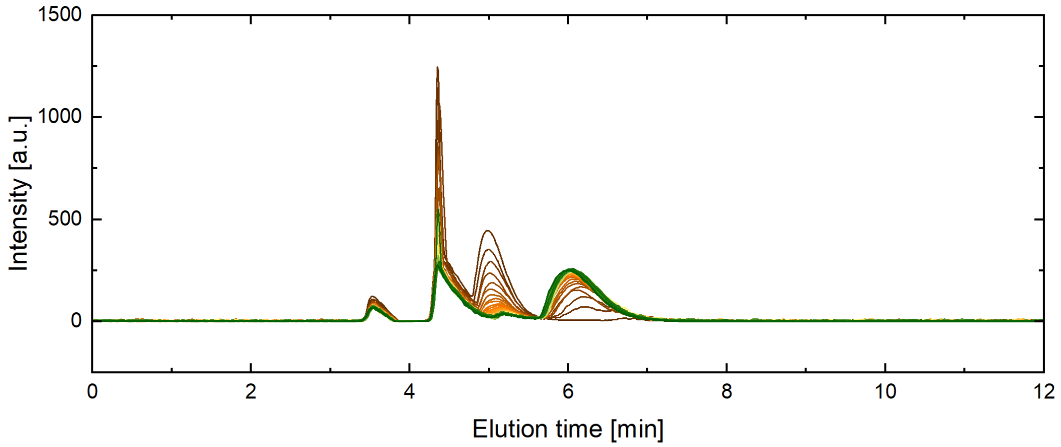

Figure S64. Combined maximum absorption HPLC trace showing the reaction of **3b** (5.00 min) and KFRGDS (4.35 min) in PBS (10 mM, pH = 7.4) to give product **6** (6.02 min).

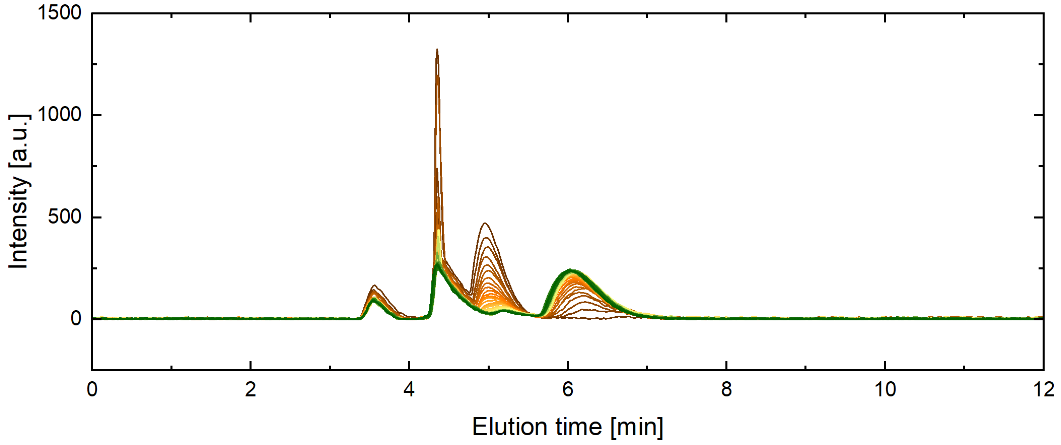

Figure S65. Combined maximum absorption HPLC trace showing the reaction of **3b** (5.00 min) and KFRGDS (4.35 min) in PBS (10 mM, pH = 7.4) to give product **6** (6.02 min).

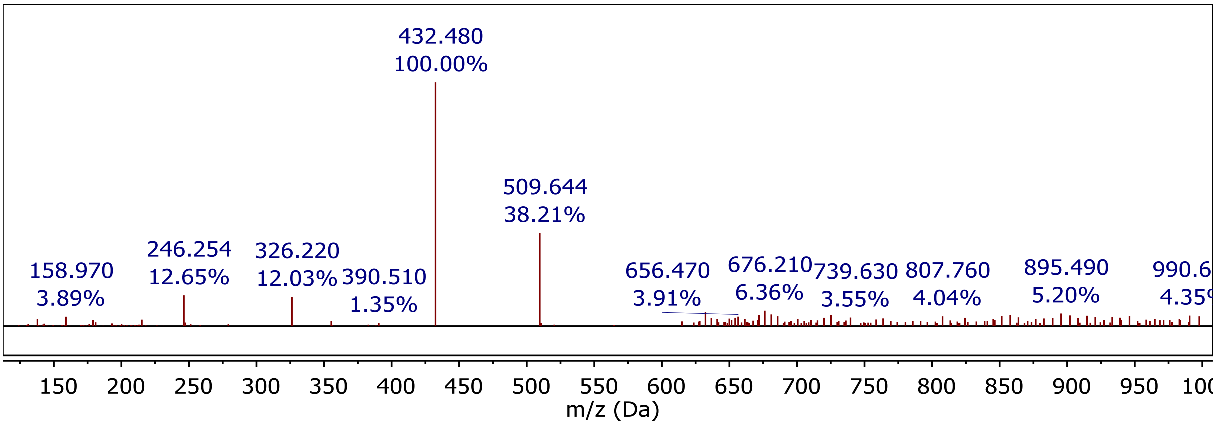

Figure S66. Mass spectrum of **6** with the doubly charged species ($\mathbf{6}$+2H)$^{2+}$ = 432.480 Da.

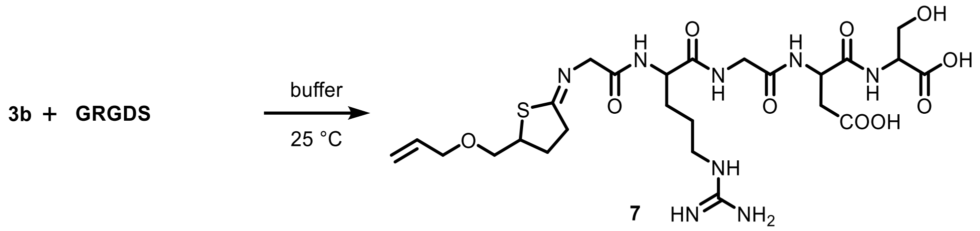

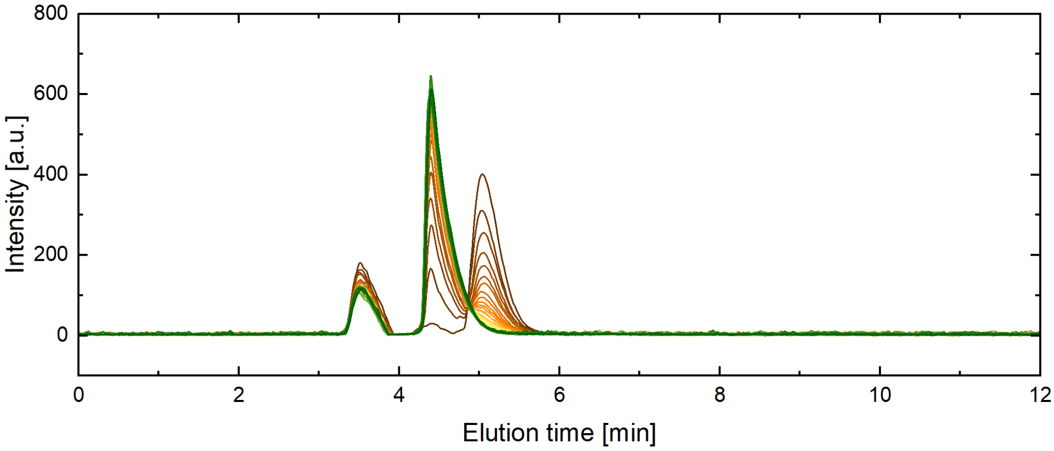

Figure S67. Combined maximum absorption HPLC trace showing the reaction of **3b** (5.04 min) and GRGDS (4.52 min) in NaPi (20 mM, pH = 8.0) to give product **7** (4.04 min).

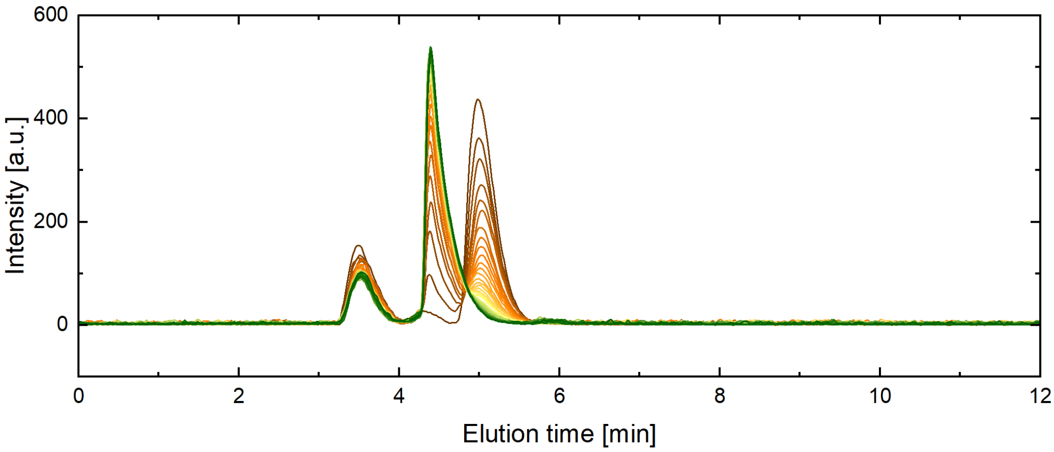

Figure S68. Combined maximum absorption HPLC trace showing the reaction of **3b** (5.04 min) and GRGDS (4.52 min) in PBS (10 mM, pH = 7.4) to give product **7** (4.04 min).

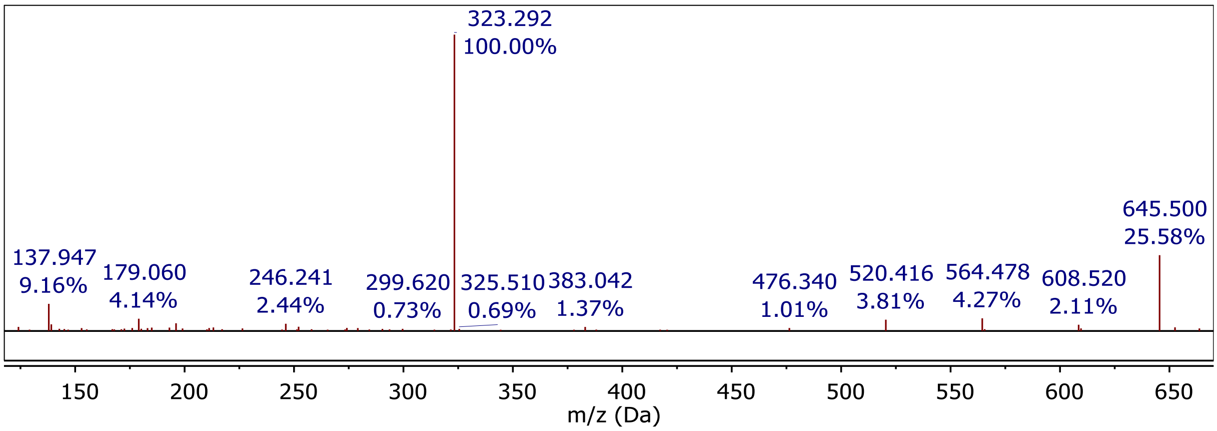

Figure S69. Mass spectrum of **7** showing the singly charged $(\mathbf{7}+H)^+$ = 323.292 Da and the doubly charged species $(\mathbf{7}+2H)^{2+}$ = 645.500 Da.

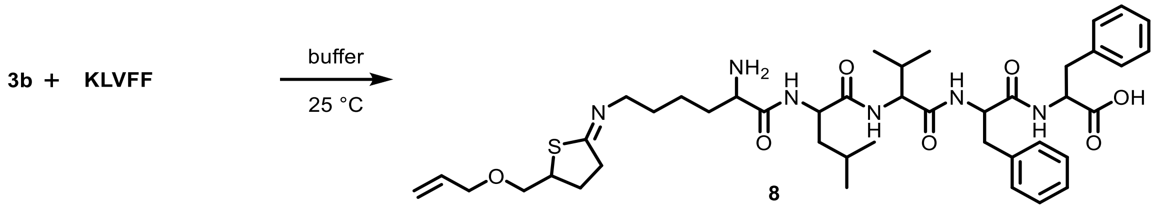

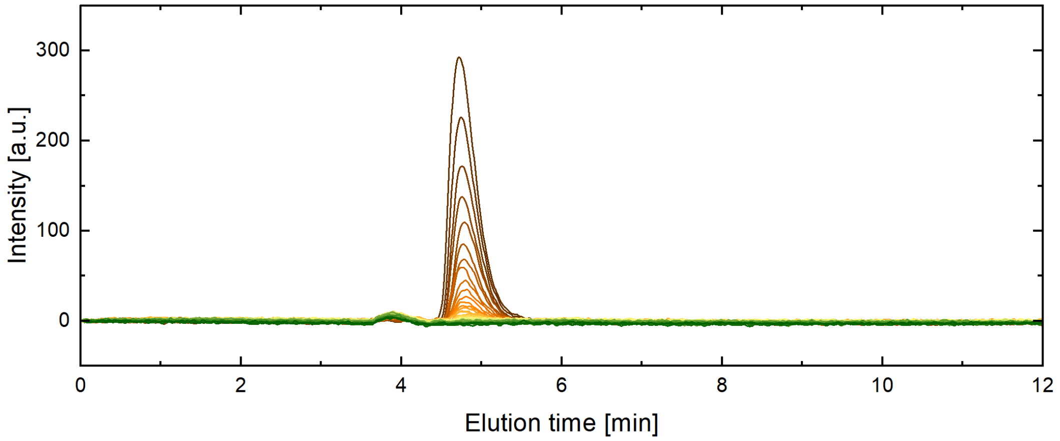

Figure S70. Absorption HPLC trace (λ = 254 nm) showing the conversion of **3b** (4.76 min) in NaPi (20 mM, pH = 8.0) to give product **8**.

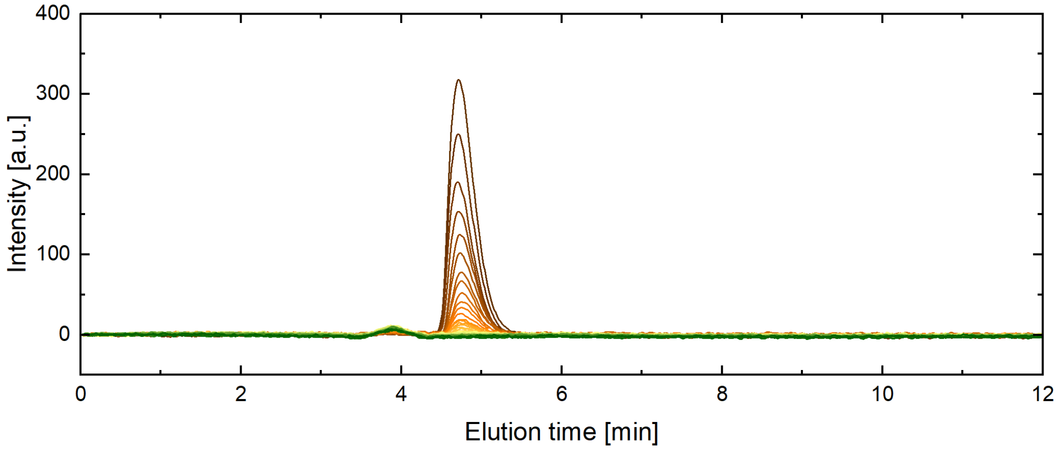

Figure S71. Absorption HPLC trace (λ = 254 nm) showing the conversion of **3b** (4.76 min) in PBS (10 mM, pH = 7.4) to give product **8**.

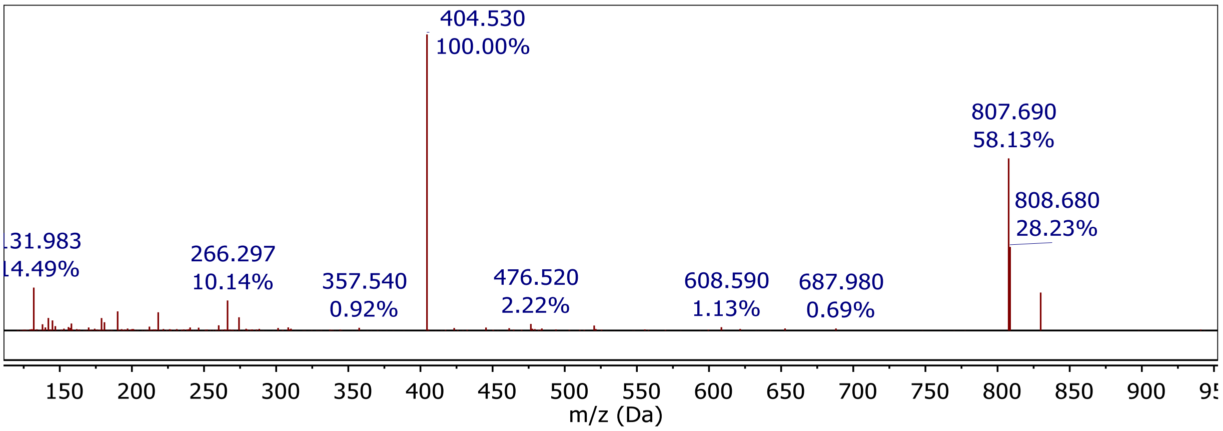

Figure S72. Mass spectrum of **8** showing the singly charged (**8**+H)$^{+}$ = 807.690 Da and the doubly charged species (**8**+2H)$^{2+}$ = 404.530 Da.

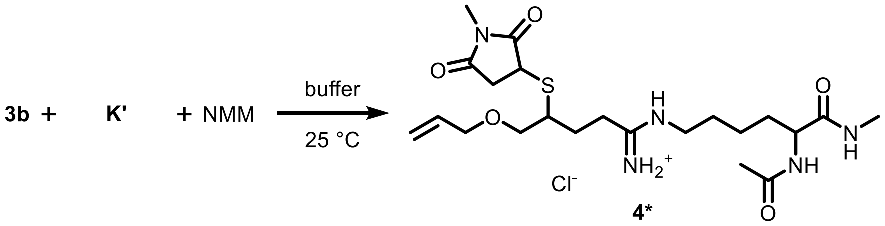

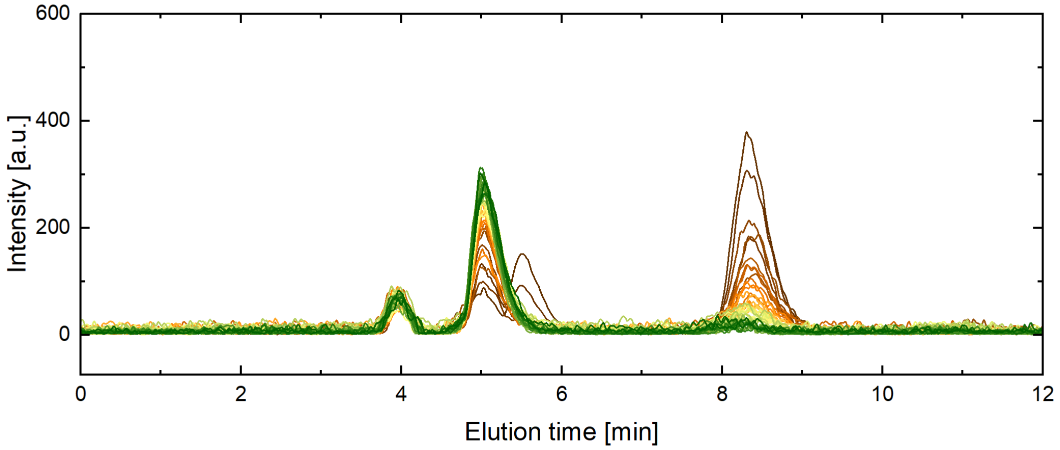

Figure S73. Combined maximum absorption HPLC trace showing the reaction of **3b** (5.50 min), **K'** (4.00 min) and NMM (8.40 min) in NaPi (20 mM, pH = 8.0) to give product **4*** (5.00 min).

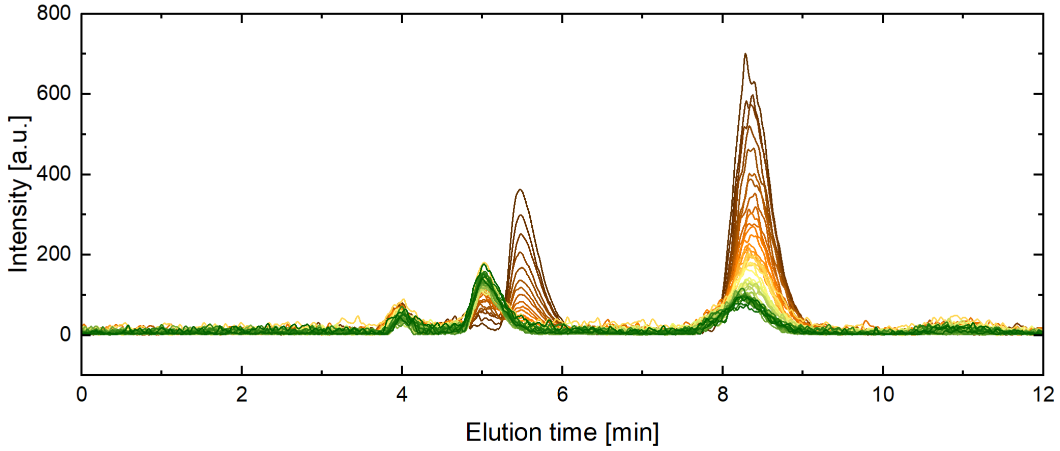

Figure S74. Combined maximum absorption HPLC trace showing the reaction of **3b** (5.50 min), **K'** (4.00 min) and NMM (8.40 min) in PBS (10 mM, pH = 7.4) to give product **4*** (5.00 min).

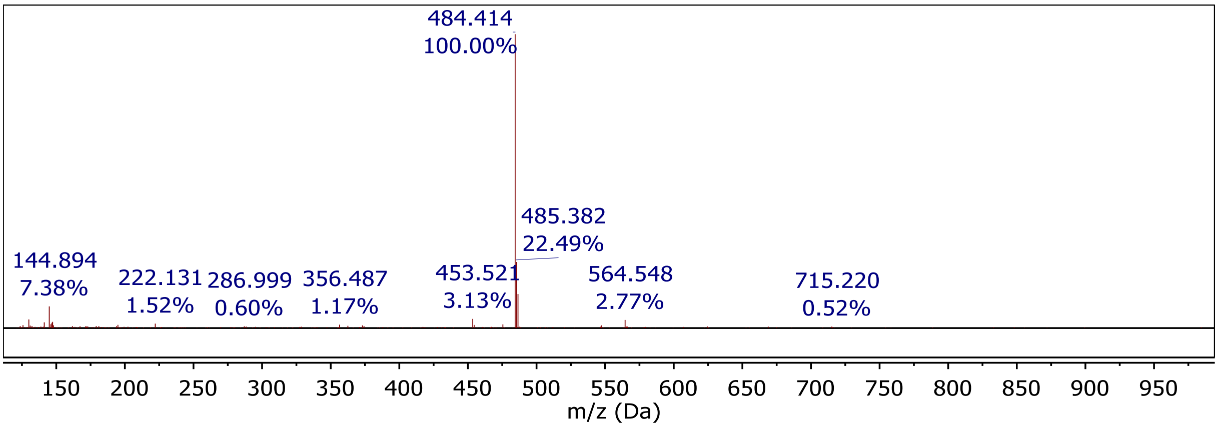

Figure S75. Mass spectrum of **4*** showing the singly charged species (**4***+H)$^{+}$ = 484.414 Da.

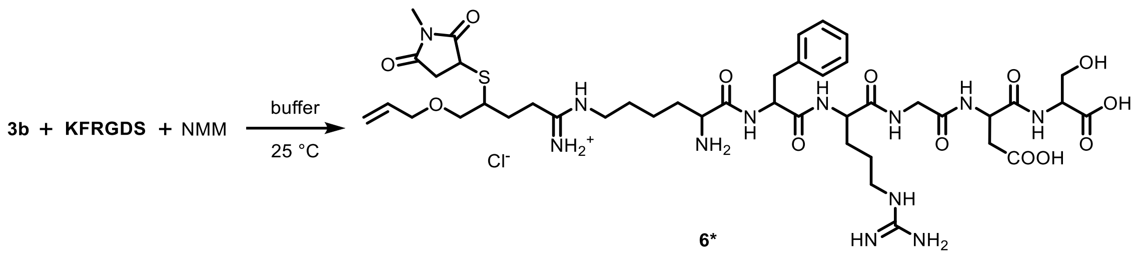

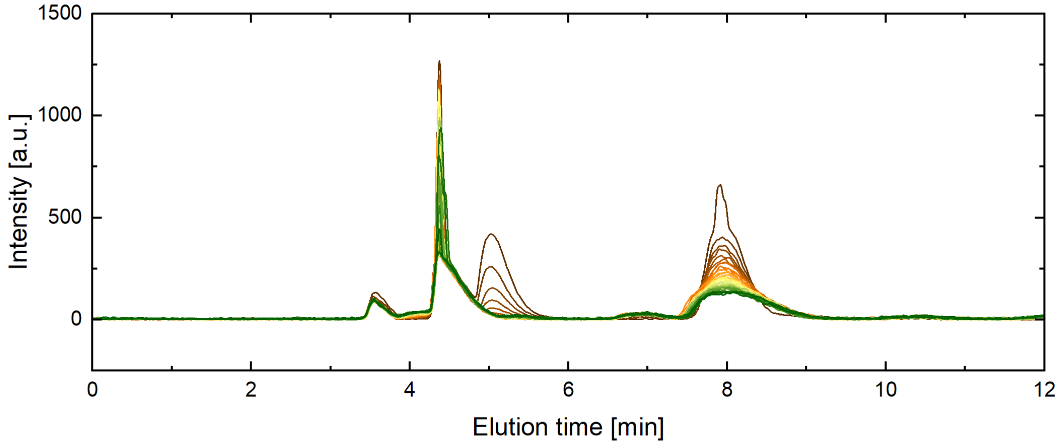

Figure S76. Combined maximum absorption HPLC trace showing the reaction of **3b** (5.00 min), KFRGDS (4.35 min) and NMM (7.88 min) in NaPi (20 mM, pH = 8.0) to give product **6*** (4.57 min).

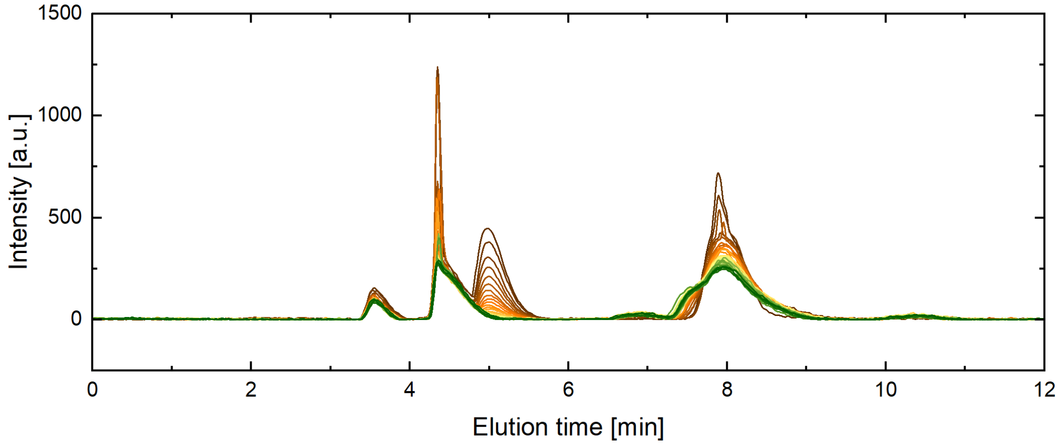

Figure S77. Combined maximum absorption HPLC trace showing the reaction of **3b** (5.00 min), KFRGDS (4.35 min) and NMM (7.88 min) in PBS (10 mM, pH = 7.4) to give product **6*** (4.57 min).

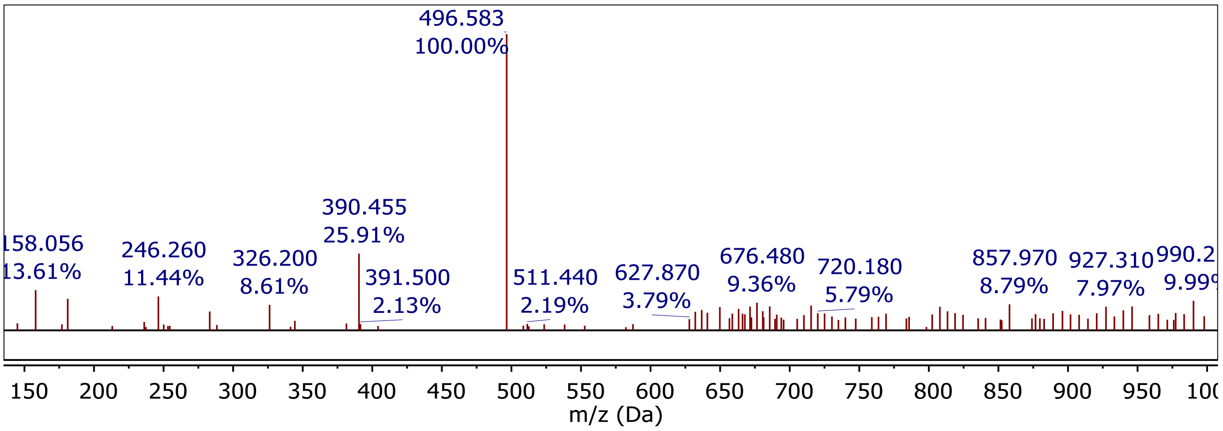

Figure S78. Mass spectrum of **6*** with the doubly charged species (**6***+H)$^{2+}$ = 496.583 Da.

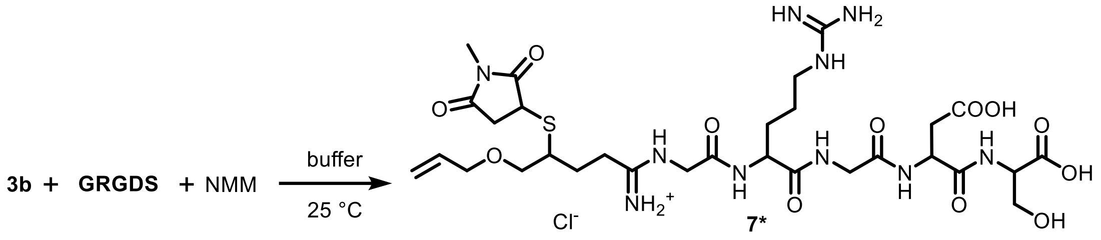

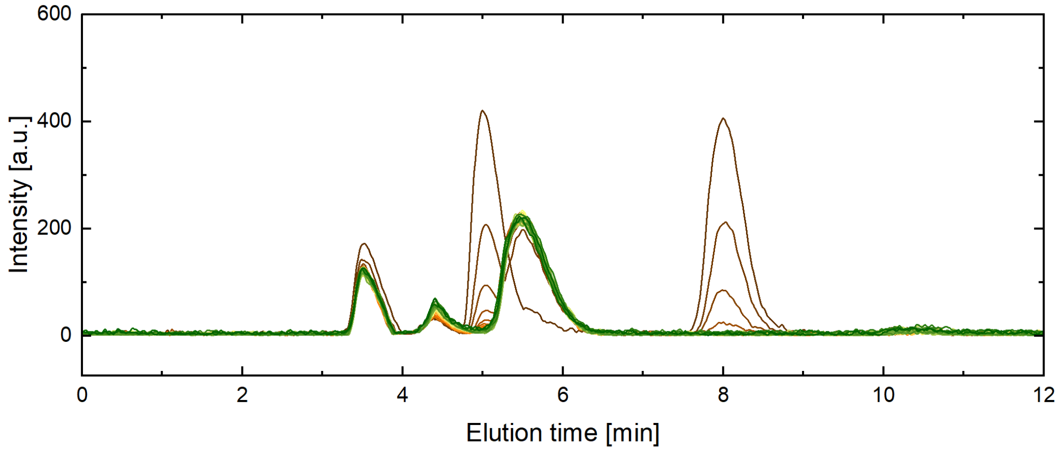

Figure S79. Combined maximum absorption HPLC trace showing the reaction of **3b** (5.00 min), GRGDS (4.35 min) and NMM (8.00 min) in NaPi (20 mM, pH = 8.0) to give product **7*** (5.48 min).

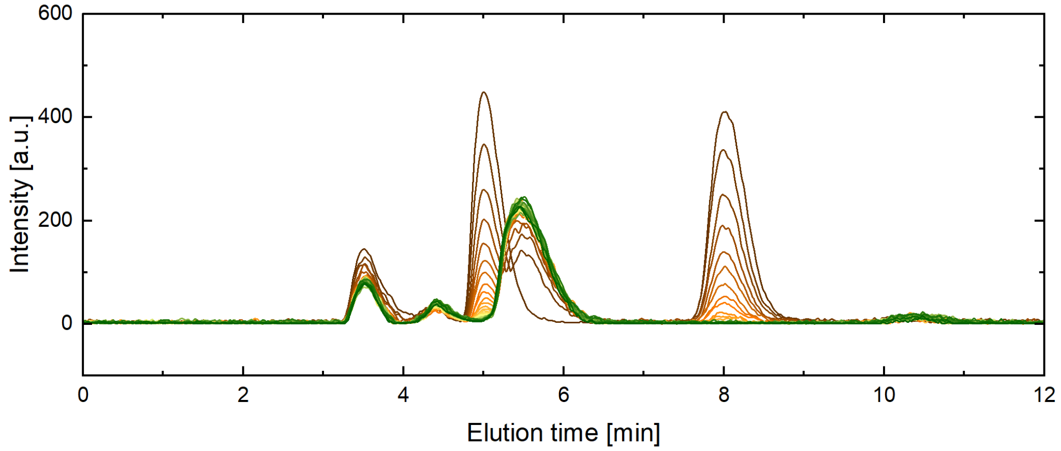

Figure S80. Combined maximum absorption HPLC trace showing the reaction of **3b** (5.00 min), GRGDS (4.35 min) and NMM (8.00 min) in PBS (10 mM, pH = 7.4) to give product **7*** (5.48 min).

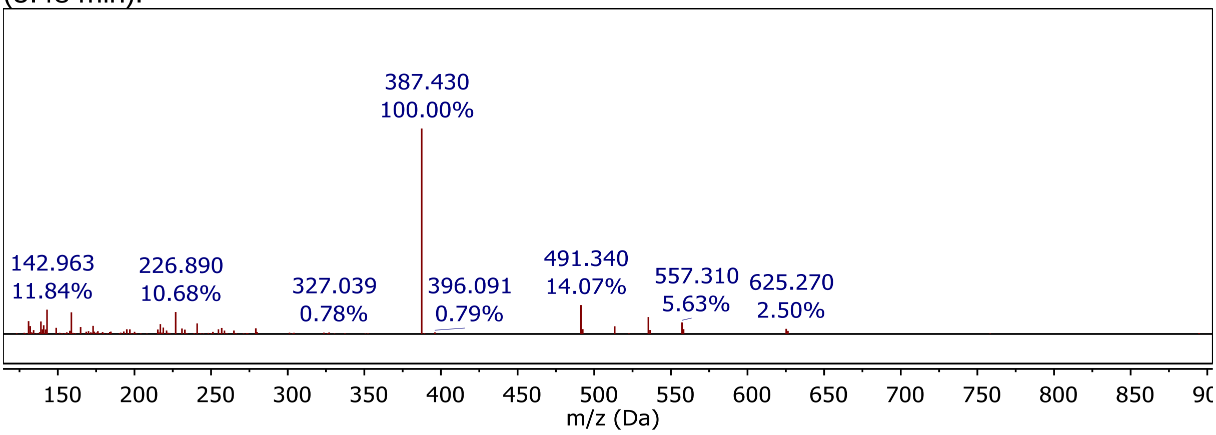

Figure S81. Mass spectrum of **7*** showing the doubly charged species (**7***+H)$^{2+}$ = 387.430 Da.

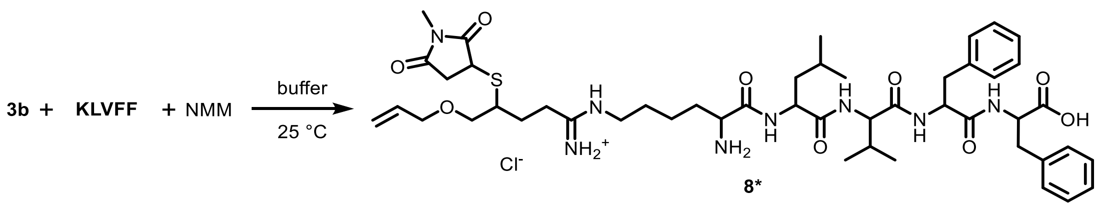

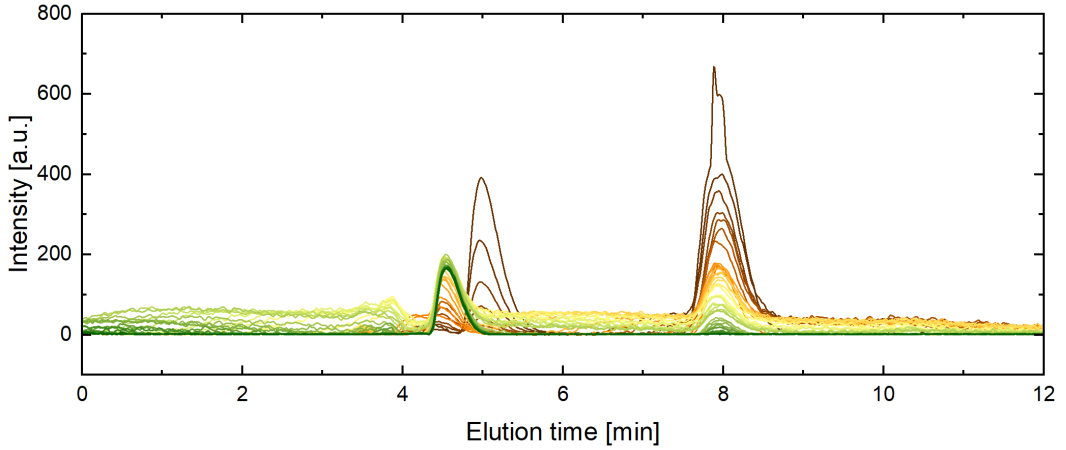

Figure S82. Combined maximum absorption HPLC trace showing the reaction of **3b** (5.00 min), KLVFF and NMM (8.00 min) in NaPi (20 mM, pH = 8.0) to give product **8*** (4.56 min).

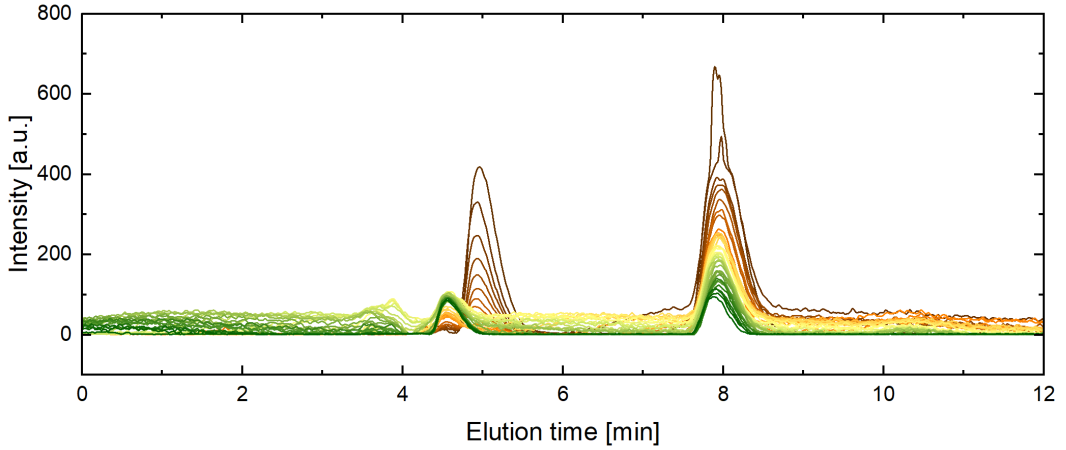

Figure S83. Combined maximum absorption HPLC trace showing the reaction of **3b** (5.00 min), KLVFF and NMM (8.00 min) in PBS (10 mM, pH = 7.4) to give product **8*** (4.56 min).

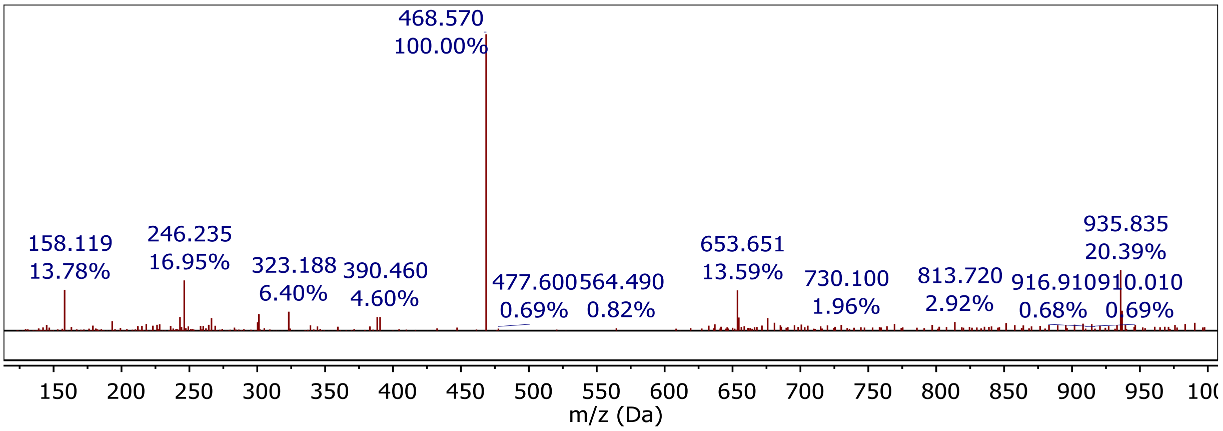

Figure S84. Mass spectrum of **8*** showing the singly charged (**8***)$^{+}$ = 935.835 Da and the doubly charged species (**8***+H)$^{2+}$ = 468.570 Da.

6 Protein modification

All stock solutions were prepared freshly in water.

Single modification:

Stock solution **A** comprised **3b** (8.4 mg/mL in H_2O), and stock solution **B** contained Lysozyme C (10 mg/mL in H_2O). Solution **A** (12 μL) was diluted with 20 mM NaPi buffer (916 μL, pH = 8.0). To start the reaction, solution **B** (72 μL) was added to the reaction mixture to start the protein modification (final solution: 1.0 mL, [**3b**] = 0.5 mM, 10 eq, [Lysozyme C] = 0.05 mM = 50 μM). Volumes of solution **A** were adjusted to correspond to 20, 30 and 50 eq referring to lysozyme C.

Double modification:

The following stock solutions were prepared: **A**) **3b** (8.4 mg/mL in H_2O), **B**) Lysozyme C (10 mg/mL in H_2O) and **C**) NMM (8.4 mg/mL in H_2O). Solution **A** (12 μL) and solution **C** (7 μL) were added to a vial filled with 20 mM NaPi (909 μL, pH = 8.0). To start the reaction, solution **B** (72 μL) was added to the reaction mixture to start the protein modification (final solution: 1.0 mL, [**3b**] = 0.5 mM, [NMM] = 0.5 mM, both 10 eq; [Lysozyme C] = 50 μM). Volumes of solution **A** and **C** were adjusted to correspond to 20, 30 and 50 eq referring to lysozyme C.

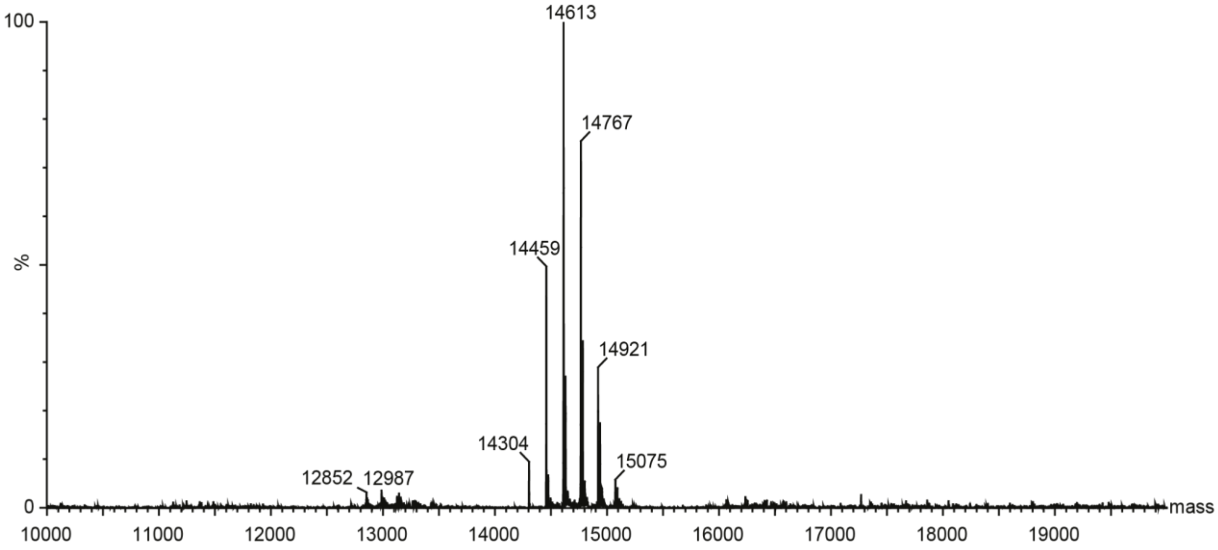

Figure S85. MaxEnt deconvoluted mass spectrum of the single modification with lysozyme C and 10 eq of **3b**. Most dominant species: 2 lysines functionalized (mass = 14613).

Table S6. Mass spectrometry data of lysozyme C subjected to single modification with 10 eq **3b** (Δ = +154.23 g/mol).

Degree of functionalization	*m/z* (calculated)	*m/z* (found)	Intensity (%)
0	14305	14304	9
1	14459.23	14459	50
2	14613.46	14613	100
3	14767.69	14767	75
4	14921.92	14921	28
5	15076.15	15075	6

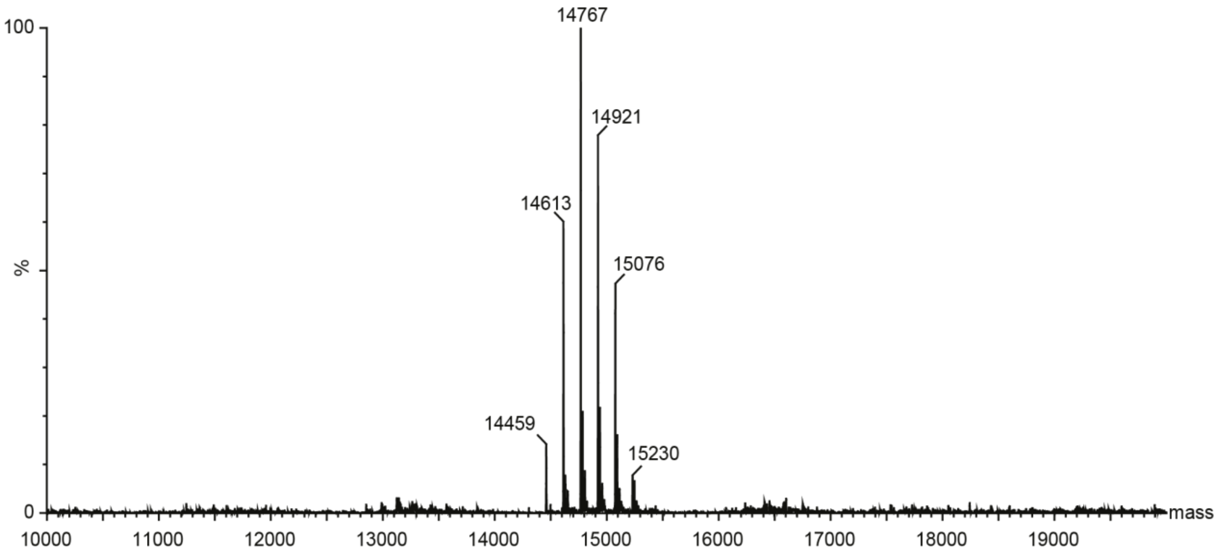

Figure S86. MaxEnt deconvoluted mass spectrum of the single modification with lysozyme C and 20 eq of **3b**. Most dominant species: 3 lysines functionalized (mass = 14767).

Table S7. Mass spectrometry data of lysozyme C subjected to single modification with 20 eq **3b** (Δ = +154.23 g/mol).

Degree of functionalization	*m/z* (calculated)	*m/z* (found)	Intensity (%)
1	14459.23	14459	12
2	14613.46	14613	60
3	14767.69	14767	100
4	14921.92	14921	78
5	15076.15	15076	48
6	15230.38	15230	8

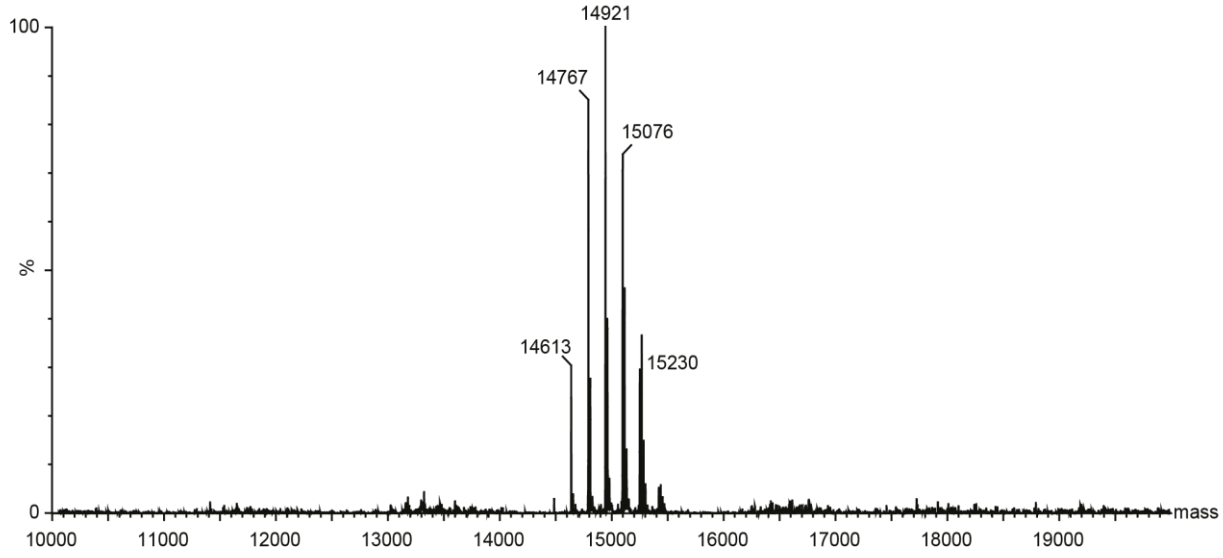

Figure S87. MaxEnt deconvoluted mass spectrum of the single modification with lysozyme C and 30 eq of **3b**. Most dominant species: 4 lysines functionalized (mass = 14921).

Table S8. Mass spectrometry data of lysozyme C subjected to single modification with 30 eq **3b** (Δ = +154.23 g/mol).

Degree of functionalization	*m/z* (calculated)	*m/z* (found)	Intensity (%)
2	14613.46	14613	30
3	14767.69	14767	85
4	14921.92	14921	100
5	15076.15	15076	73
6	15230.38	15230	30

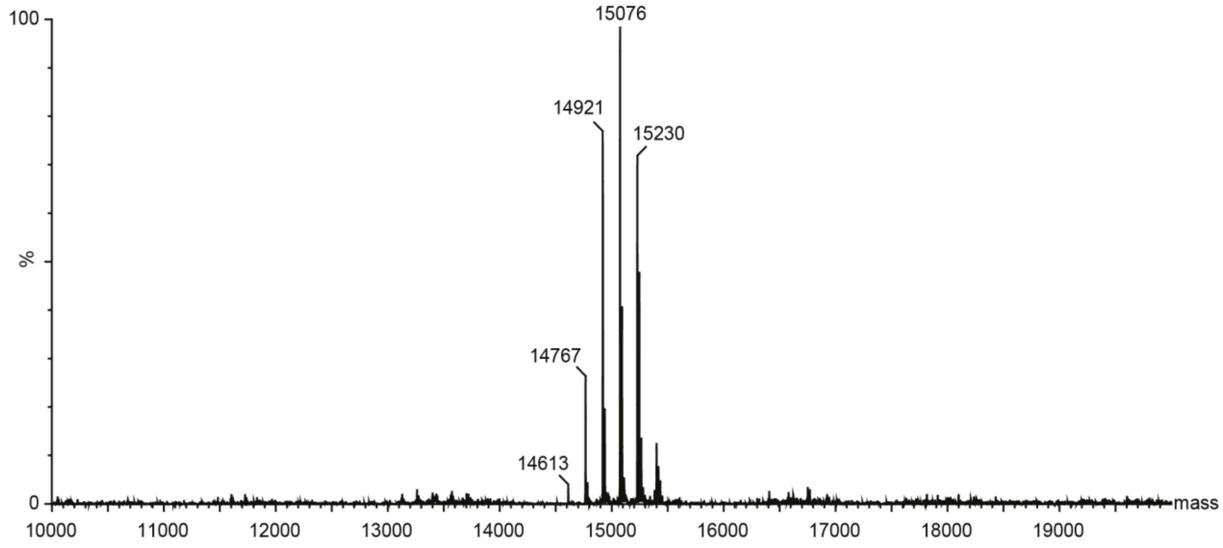

Figure S88. MaxEnt deconvoluted mass spectrum of the single modification with lysozyme C and 50 eq of **3b**. Most dominant species: 5 lysines functionalized (mass = 15076).

Table S9. Mass spectrometry data of lysozyme C subjected to single modification with 50 eq **3b** (Δ = +154.23 g/mol).

Degree of functionalization	*m/z* (calculated)	*m/z* (found)	Intensity (%)
2	14613.46	14613	3
3	14767.69	14767	26
4	14921.92	14921	77
5	15076.15	15076	100
6	15230.38	15230	72

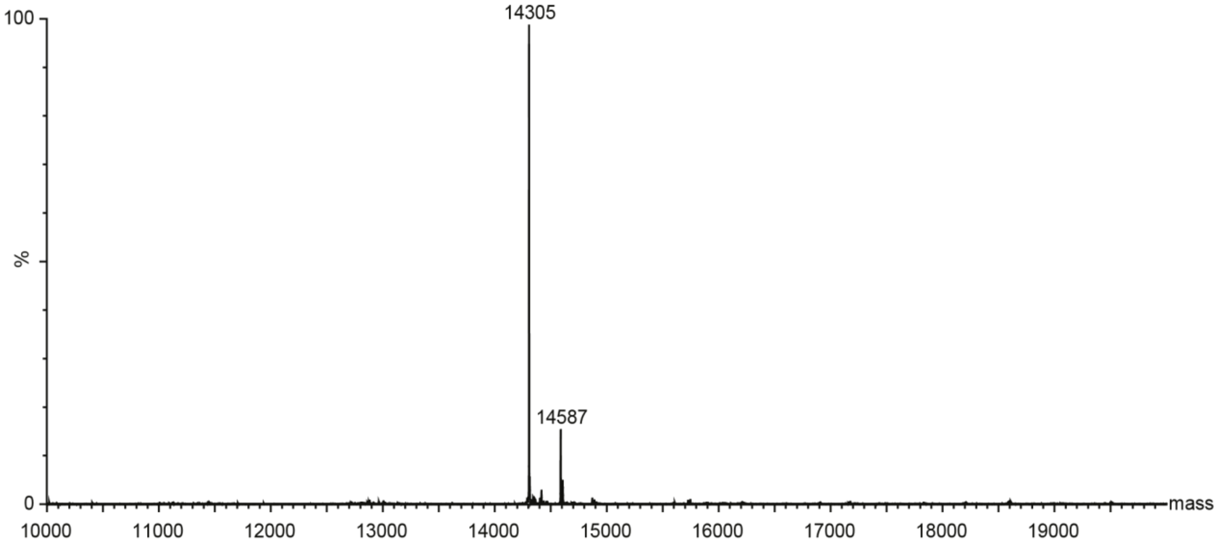

Figure S89. MaxEnt deconvoluted mass spectrum of the double modification with lysozyme C and 10 eq of **3b** and NMM. Most dominant species: lysozyme C (mass = 14305).

Table S10. Mass spectrometry data of lysozyme C subjected to double modification with 10 eq **3b** and 10 eq NMM (Δ = +282.36 g/mol).

Degree of functionalization	*m/z* (calculated)	*m/z* (found)	Intensity (%)
0	14305	14305	100
1	14587.36	14587	15

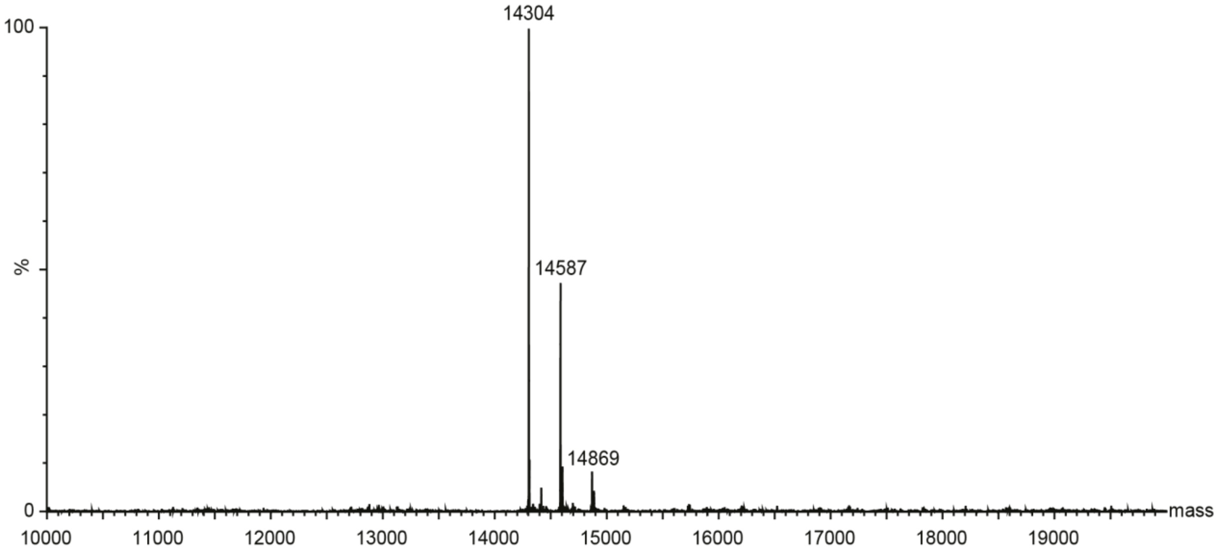

Figure S90. MaxEnt deconvoluted mass spectrum of the double modification with lysozyme C and 20 eq of **3b** and NMM. Most dominant species: lysozyme C (mass = 14304).

Table S11. Mass spectrometry data of lysozyme C subjected to double modification with 20 eq **3b** and 20 eq NMM (Δ = +282.36 g/mol).

Degree of functionalization	*m/z* (calculated)	*m/z* (found)	Intensity (%)
0	14305	14304	100
1	14587.36	14587	48
2	14869.72	14869	8

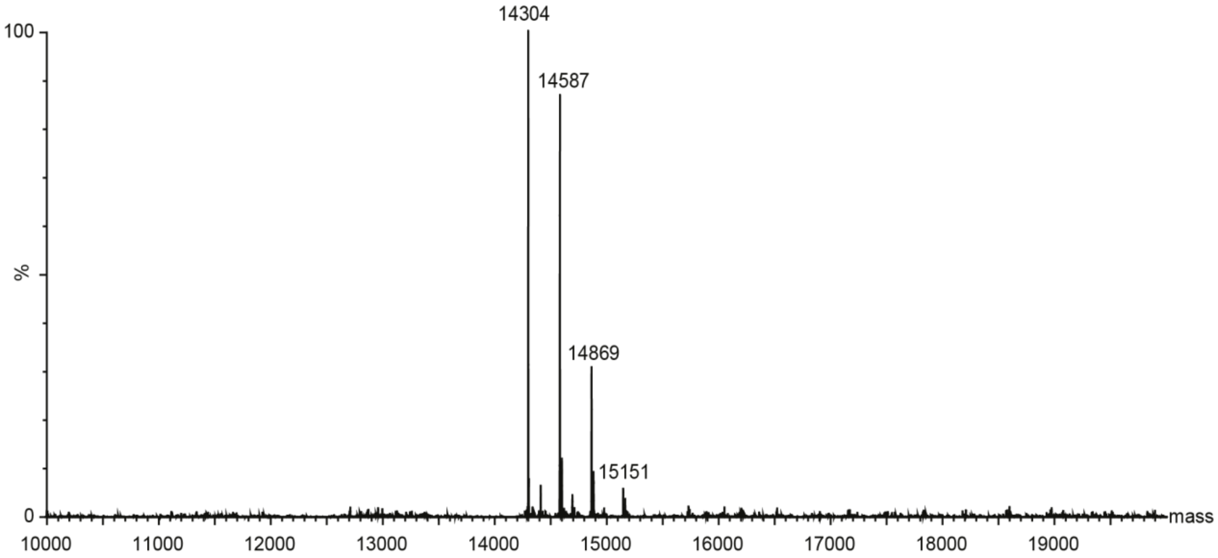

Figure S91. MaxEnt deconvoluted mass spectrum of the double modification with lysozyme C and 30 eq of **3b** and NMM. Most dominant species: lysozyme C (mass = 14304).

Table S12. Mass spectrometry data of lysozyme C subjected to double modification with 30 eq **3b** and 30 eq NMM (Δ = +282.36 g/mol).

Degree of functionalization	*m/z* (calculated)	*m/z* (found)	Intensity (%)
0	14305	14305	100
1	14587.36	14587	88
2	14869.72	14869	31
3	15152.08	15151	6

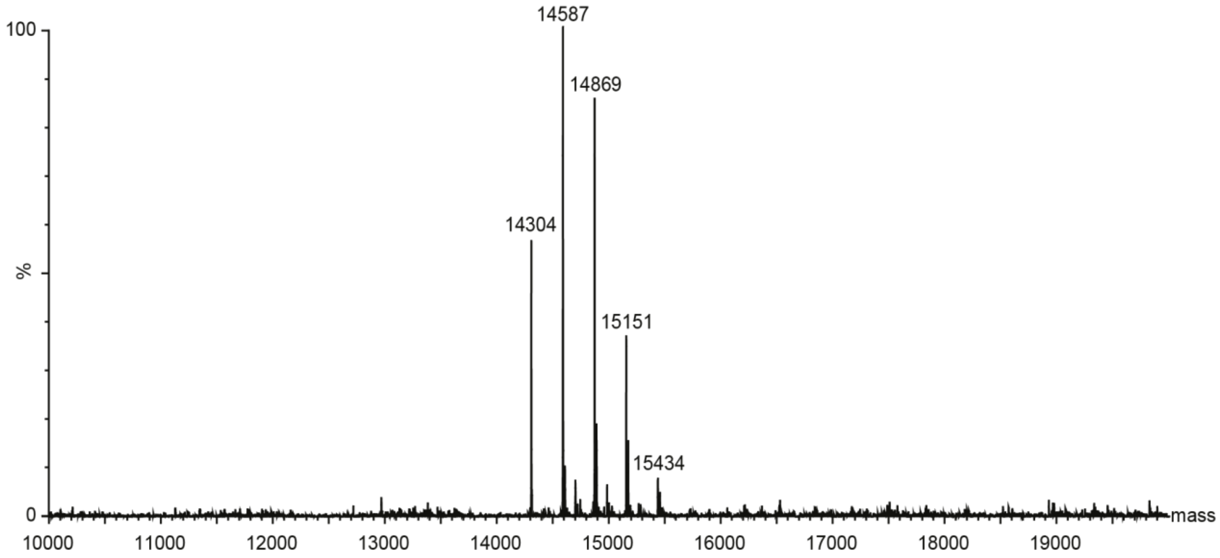

Figure S92. MaxEnt deconvoluted mass spectrum of the double modification with lysozyme C and 50 eq of **3b** and NMM. Most dominant species: 1 lysine functionalized (mass = 14587).

Table S13. Mass spectrometry data of lysozyme C subjected to double modification with 50 eq **3b** and 50 eq NMM (Δ = +282.36 g/mol).

Degree of functionalization	*m/z* (calculated)	*m/z* (found)	Intensity (%)
0	14305	14305	57
1	14587.36	14587	100
2	14869.72	14869	86
3	15152.08	15151	37
4	15434.44	15434	8

7 References

(1) Kazemi, F.; Kiasat, A. R.; Ebrahimi, S. LiBF4: A Mild and Efficient Catalyst for Conversion of Oxiranes to Thiiranes with Thiourea. *Synth. Commun.* **2003**, *33* (4), 595-600.

(2) Fu, C.; Linden, A.; Heimgartner, H. Reaction of Optically Active Oxiranes with Thiofenchone and 1-Methylpyrrolidine-2-thione: Formation of 1,3-Oxathiolanes and Thiiranes. *Helv. Chim. Acta* **2011**, *94* (5), 773-784.

(3) Aoyagi, N.; Endo, T. A Catalyst-Free and Chemoselective Synthesis of Episulfides from Epoxides in 2,3-Butanediol without Formation of Poly(episulfide)s. *ChemistrySelect* **2017**, *2* (16), 4466-4468.

(4) *APEX2*; Bruker AXS Inc.: Madison, WI, USA, 2019.

(5) *SADABS ver. 2014/05*; Bruker AXS Inc.: Madison, WI, USA, 2014.

(6) Sheldrick, G. M. SHELXT - Integrated space-group and crystal-structure determination. *Acta Crystallogr. A.* **2015**, *71* (1), 3-8.

(7) Sheldrick, G. M. Crystal structure refinement with SHELXL. *Acta Crystallogr. C.* **2015**, *71* (1), 3-8.

(8) *Gaussian 09*; Gaussian Inc.: Wallingford, CT, USA, 2016.

(9) Becke, A. D. Density-functional exchange-energy approximation with correct asymptotic behavior. *Phys. Rev. A.* **1988**, *38* (6), 3098–3100.

(10) Lee, C.; Yang, W.; Parr, R. G. Development of the Colle-Salvetti correlation-energy formula into a functional of the electron density. *Phys. Rev. B.* **1988**, *37*, 785–789.

(11) *GaussView*; Semichem Inc.: Shawnee, KS, USA, 2016.
